# Climate‐induced changes in the suitable habitat of cold‐water corals and commercially important deep‐sea fishes in the North Atlantic

**DOI:** 10.1111/gcb.14996

**Published:** 2020-02-20

**Authors:** Telmo Morato, José‐Manuel González‐Irusta, Carlos Dominguez‐Carrió, Chih‐Lin Wei, Andrew Davies, Andrew K. Sweetman, Gerald H. Taranto, Lindsay Beazley, Ana García‐Alegre, Anthony Grehan, Pascal Laffargue, Francisco Javier Murillo, Mar Sacau, Sandrine Vaz, Ellen Kenchington, Sophie Arnaud‐Haond, Oisín Callery, Giovanni Chimienti, Erik Cordes, Hronn Egilsdottir, André Freiwald, Ryan Gasbarro, Cristina Gutiérrez‐Zárate, Matthew Gianni, Kent Gilkinson, Vonda E. Wareham Hayes, Dierk Hebbeln, Kevin Hedges, Lea‐Anne Henry, David Johnson, Mariano Koen‐Alonso, Cam Lirette, Francesco Mastrototaro, Lénaick Menot, Tina Molodtsova, Pablo Durán Muñoz, Covadonga Orejas, Maria Grazia Pennino, Patricia Puerta, Stefán Á. Ragnarsson, Berta Ramiro‐Sánchez, Jake Rice, Jesús Rivera, J. Murray Roberts, Steve W. Ross, José L. Rueda, Íris Sampaio, Paul Snelgrove, David Stirling, Margaret A. Treble, Javier Urra, Johanne Vad, Dick van Oevelen, Les Watling, Wojciech Walkusz, Claudia Wienberg, Mathieu Woillez, Lisa A. Levin, Marina Carreiro‐Silva

**Affiliations:** ^1^ Okeanos Research Centre Departamento de Oceanografia e Pesca Universidade dos Açores Horta Portugal; ^2^ IMAR Instituto do Mar Departamento de Oceanografia e Pesca Universidade dos Açores Horta Portugal; ^3^ Institute of Oceanography National Taiwan University Taipei Taiwan; ^4^ Department of Biological Sciences University of Rhode Island Kingston RI USA; ^5^ Marine Benthic Ecology, Biogeochemistry and In situ Technology Research Group The Lyell Centre for Earth and Marine Science and Technology Heriot‐Watt University Edinburgh UK; ^6^ Fisheries and Oceans Canada Bedford Institute of Oceanography Dartmouth NS Canada; ^7^ Instituto Español de Oceanografía (IEO) Centro Oceanográfico de Vigo Vigo, Pontevedra Spain; ^8^ Earth and Ocean Sciences NUI Galway Galway Ireland; ^9^ IFREMER, Centre Atlantique Nantes France; ^10^ MARBEC University of Montpellier IFREMER CNRS IRD Sète France; ^11^ Department of Biology University of Bari Aldo Moro Bari Italy; ^12^ CoNISMa Rome Italy; ^13^ Department of Biology Temple University Philadelphia PA USA; ^14^ Marine and Freshwater Research Institute Reykjavík Iceland; ^15^ Marine Research Department Senckenberg am Meer Wilhelmshaven Germany; ^16^ Gianni Consultancy Amsterdam The Netherlands; ^17^ Northwest Atlantic Fisheries Centre Fisheries and Ocean Canada St. John’s NL Canada; ^18^ MARUM ‐ Center for Marine Environmental Sciences University of Bremen Bremen Germany; ^19^ Fisheries and Oceans Canada Winnipeg MB Canada; ^20^ Changing Oceans Group School of GeoSciences Grant Institute University of Edinburgh Edinburgh UK; ^21^ Seascape Consultants Ltd Romsey UK; ^22^ IFREMER Centre de Bretagne Plouzané France; ^23^ P.P. Shirshov Institute of Oceanology RAS Moscow Russia; ^24^ Instituto Español de Oceanografía Centro Oceanográfico de Baleares Palma Spain; ^25^ Fisheries and Ocean Canada Ottawa ON Canada; ^26^ Instituto Español de Oceanografía Madrid Spain; ^27^ Center for Marine Science University of North Carolina at Wilmington Wilmington NC USA; ^28^ Instituto Español de Oceanografía Centro Oceanográfico de Málaga Málaga Spain; ^29^ Ocean Sciences Centre Memorial University St. John’s NL Canada; ^30^ Marine Laboratory Marine Scotland Science Aberdeen UK; ^31^ Royal Netherlands Institute for Sea Research (NIOZ) Utrecht University Yerseke The Netherlands; ^32^ Department of Biology University of Hawai‘i at Mānoa Honolulu HI USA; ^33^ Center for Marine Biodiversity and Conservation and Integrative Oceanography Division Scripps Institution of Oceanography UC San Diego La Jolla CA USA

**Keywords:** climate change, cold‐water corals, deep‐sea, fisheries, fishes, habitat suitability modelling, octocorals, scleractinians, species distribution models, vulnerable marine ecosystems

## Abstract

The deep sea plays a critical role in global climate regulation through uptake and storage of heat and carbon dioxide. However, this regulating service causes warming, acidification and deoxygenation of deep waters, leading to decreased food availability at the seafloor. These changes and their projections are likely to affect productivity, biodiversity and distributions of deep‐sea fauna, thereby compromising key ecosystem services. Understanding how climate change can lead to shifts in deep‐sea species distributions is critically important in developing management measures. We used environmental niche modelling along with the best available species occurrence data and environmental parameters to model habitat suitability for key cold‐water coral and commercially important deep‐sea fish species under present‐day (1951–2000) environmental conditions and to project changes under severe, high emissions future (2081–2100) climate projections (RCP8.5 scenario) for the North Atlantic Ocean. Our models projected a decrease of 28%–100% in suitable habitat for cold‐water corals and a shift in suitable habitat for deep‐sea fishes of 2.0°–9.9° towards higher latitudes. The largest reductions in suitable habitat were projected for the scleractinian coral *Lophelia pertusa* and the octocoral *Paragorgia arborea*, with declines of at least 79% and 99% respectively. We projected the expansion of suitable habitat by 2100 only for the fishes *Helicolenus dactylopterus* and *Sebastes mentella *(20%–30%), mostly through northern latitudinal range expansion. Our results projected limited climate refugia locations in the North Atlantic by 2100 for scleractinian corals (30%–42% of present‐day suitable habitat), even smaller refugia locations for the octocorals *Acanella arbuscula* and *Acanthogorgia armata* (6%–14%), and almost no refugia for *P. arborea*. Our results emphasize the need to understand how anticipated climate change will affect the distribution of deep‐sea species including commercially important fishes and foundation species, and highlight the importance of identifying and preserving climate refugia for a range of area‐based planning and management tools.

## Graphical abstract

We used environmental niche modelling to predict the habitat suitability for key cold‐water coral and commercially important deep‐sea fish species under present‐day environmental conditions and to forecast changes under future climate projections (RCP8.5) for the North Atlantic Ocean. Our models forecasted a significant decrease in suitable habitat for cold‐water corals and poleward expansion in suitable habitat for deep‐sea fishes in response to climate change. Our results emphasize the need to understand how climate change will affect the distribution of deep‐sea species and highlight the importance of identifying and preserving climate refugia for a range of area‐based planning and management tools.

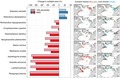

## 1 INTRODUCTION

The deep sea represents at least 95% of the ocean and plays a critical role in climate regulation through uptake and storage of heat and carbon dioxide (Purkey & Johnson, 2010; Sabine et al., 2004). However, changes linked to these regulating services have consequences for the health of the ocean including warming, acidification, and deoxygenation of deep waters, leading to decrease in food availability at the seafloor (Bindoff et al., 2019; Chen et al., 2017; Gehlen et al., 2014; Mora et al., 2013; Perez et al., 2018; Sulpis et al., 2018; Sweetman et al., 2017). Recent projections of deep water mass properties suggested that portions of the seafloor will experience average temperature increases in excess of 1°C, pH decreases greater than 0.3 units, dissolved oxygen decreases up to 3.7%, and a 40%–55% decrease in particulate organic matter flux to the seafloor by 2100 (Gehlen et al., 2014; Sweetman et al., 2017). These projected changes may severely affect productivity, biodiversity, and distribution of deep‐sea fauna, including species that underpin vulnerable marine ecosystems (VMEs) as well as commercially important deep‐sea fishes, thereby compromising key ecosystem services (Johnson, Ferreira, & Kenchington, 2018; Jones et al., 2014; Levin & Le Bris, 2015; Pecl et al., 2017; Thurber et al., 2014).

Among deep‐sea VME indicators, cold‐water corals that form important biogenic habitats are known to be vulnerable to anthropogenic climate change, particularly to ocean acidification (FAO, 2019; Guinotte et al., 2006; Orr et al., 2005; Perez et al., 2018; Roberts et al., 2016; Tittensor, Baco, Hall‐Spencer, Orr, & Rogers, 2010). This vulnerability exists because most cold‐water corals with carbonate skeletons occur in waters supersaturated in carbonate that enable coral skeleton biocalcification. Although several experimental studies demonstrate high resilience of reef‐building scleractinian to ocean acidification (Büscher, Form, & Riebesell, 2017; Form & Riebesell, 2012; Hennige et al., 2014, 2015; Maier et al., 2013; Maier, Watremez, Taviani, Weinbauer, & Gattuso, 2012; Maier, Weinbauer, & Gattuso, 2019; Movilla et al., 2014), the projected shoaling of the calcite and aragonite saturation horizons along with warming is expected to lead to the loss of suitable habitat (Davies & Guinotte, 2011; Perez et al., 2018; Sulpis et al., 2018; Tittensor et al., 2010; Yesson et al., 2012), weakening of the reef frameworks that may result in structural collapse of slow‐growing scleractinian corals (Büscher et al., 2019; Gomez, Wickes, Deegan, Etnoyer, & Cordes, 2019; Hennige et al., 2015), and increased mortality of octocorals that form coral gardens (Cerrano et al., 2013; Gugliotti, DeLorenzo, & Etnoyer, 2019). Notwithstanding genotypic variability in cold‐water corals’ response to ocean acidification (Kurman, Gómez, Georgian, Lunden, & Cordes, 2017; Lunden, McNicholl, Sears, Morrison, & Cordes, 2014), these changes may result in the loss of biodiversity and provision of ecosystem services associated with these ecosystems (Cordes et al., 2016).

Along with climate regulation, provisioning of food from fish stocks is one of the most critical ecosystem services provided by the deep sea (Thurber et al., 2014); these stocks are increasingly important to global food security (Victorero, Watling, Palomares, & Nouvian, 2018; Watson & Morato, 2013). However, warming and deoxygenation will simultaneously affect fishes by increasing metabolic rates and oxygen demand while limiting supply of oxygen to their tissues to meet increased demand for oxygen in low‐oxygen environments (Holt & Jørgensen, 2015; Pörtner, Bock, & Mark, 2017; Pörtner & Knust, 2007). Decreasing food availability will indirectly exacerbate stress imposed by increased metabolism in warmer waters (Woodworth‐Jefcoats, Polovina, & Drazen, 2017). Although, many gaps remain in understanding the underlying physiological mechanisms that influence potential responses of fishes to climate change (Lefevre, McKenzie, & Nilsson, 2017), multiple lines of evidence project that climate change will reduce the fish size and growth, abundance and survival, and will shift the spatial distributions of bottom fishes and fisheries (Baudron, Needle, Rijnsdorp, & Marshall, 2014; Bryndum‐Buchholz et al., 2019; Cheung et al., 2010; Dulvy et al., 2008; Pecl et al., 2017; Perry, Low, Ellis, & Reynolds, 2005; Pörtner & Knust, 2007), potentially driving unforeseen and undocumented trophic cascade effects (Frank, Petrie, Choi, & Leggett, 2005). Previous studies demonstrate the importance of local stocks with adaptive diversity to long‐term sustainability of fish stocks, fisheries, and ecosystems (Bradbury et al., 2010).

Improved projections of how climate change can lead to shifts in the distribution of deep‐sea species is critically important in developing effective management measures that account for such changes, especially those spatial measures that aim to preserve refugia areas or local fish stocks, to aid conservation of VMEs, or secure food, income and livelihoods from fisheries (Bates et al., 2019; Cheung et al., 2017, 2010; Gaines et al., 2018; Thresher, Guinotte, Matear, & Hobday, 2015; Tittensor et al., 2010). Such improved projections can also inform the designation of ‘other effective area‐based conservation measures’ (OECMs; CBD, 2018a; IUCN WCPA, 2019). Environmental niche modelling, also known as species distribution modelling, habitat suitability modelling (HSM) or climate envelope modelling, represents a powerful tool for predicting the distribution of species over wide geographic regions and projecting changes under future climate scenarios (Hattab et al., 2014; Hijmans & Graham, 2006; Pearson & Dawson, 2003; Wiens, Stralberg, Jongsomjit, Howell, & Snyder, 2009). While acknowledging that species genetic variability, phenotypic plasticity, evolutionary changes and acclimation could limit the accuracy of such models (Austin & Van Niel, 2011; Elith & Leathwick, 2009; Fillinger & Richter, 2013; Kurman et al., 2017; Pearson & Dawson, 2003; Sandblom, Gräns, Axelsson, & Seth, 2014), researchers have widely applied these approaches to terrestrial (e.g. Fordham et al., 2012; Iverson & Prasad, 1998) and marine species and habitats (e.g. benthic macrofauna, Singer, Millat, Staneva, & Kroncke, 2017; seagrass, Chefaoui, Duarte, & Serrão, 2018; and fish, Morley et al., 2018). With few exceptions (Tittensor et al., 2010), the lack of reliable projections of future environmental conditions close to the seabed has constrained efforts to project shifts in distributions of deep‐sea bottom‐dwelling species.

The recent modelling of global‐scale scenarios for future deep ocean environmental conditions (e.g. Sweetman et al., 2017), in tandem with increased understanding of the ecology and distribution of key deep‐sea benthic species (e.g. Orejas & Jiménez, 2019; Priede, 2017; Rossi, Bramanti, Gori, & Orejas, 2017), enabled projections of distributional changes in deep‐sea species (FAO, 2019). Utilizing the best available curated species occurrence data obtained from multiple public and restricted sources, along with a set of static (depth, slope, among others) and near‐bottom dynamic environmental parameters (particulate organic carbon flux to the seabed, near seafloor pH, dissolved oxygen concentration and temperature, and near seafloor aragonite and calcite saturation state), we modelled habitat suitability for six cold‐water coral and six deep‐sea fish species under current conditions and projected changes under future projected high emission climate conditions for the whole North Atlantic Ocean. With this study, we asked how much suitable habitat we expect will be lost, gained or sustained as refugia areas under the business‐as‐usual emissions trajectory RCP8.5for indicators of VMEs and commercially important deep‐sea fishes at an ocean basin scale, to support climate change adaptive management.

## 2 MATERIALS AND METHODS

### 2.1 Study area

Habitat suitability models of VME indicator taxa and commercially important deep‐sea fish species were developed for the deep waters of the North Atlantic, from 18°N to 76°N and 36°E to 98°W. This region encompasses one of the best‐studied deep‐water regions in the world with respect to species distribution, environmental conditions and deep‐sea species responses to environmental variability. Additionally, the North Atlantic Ocean contains two well‐established Regional Fisheries Management Organisations, increasing the relevance of these analyses for fisheries management, for conserving and protecting VMEs, and for the designation of OECMs. This basin‐scale focus also enhances model performance because it accounts for a wide range of environmental variability and species’ ecological niches.

### 2.2 Species selection and presence data

Six VME indicator taxa and six commercially important deep‐sea fish species representative of both Eastern and Western North Atlantic deep‐sea habitats were selected based on their wide spatial distribution, ecological significance or catch relevance in deep‐sea fisheries, and on the availability and spatial coverage of existing occurrence records (Table 1). The VME indicator taxa included three scleractinian corals that form aragonite skeletons (*Lophelia pertusa*,1Recently synonymized to *Desmophyllum pertusum* (Addamo et al., 2016).
*Madrepora oculata* and *Desmophyllum dianthus*) and three octocorals forming calcitic axial skeletons (*Acanella arbuscula*), and with sclerites in their axis or coenenchyme and polyps (*Acanthogorgia armata*, and *Paragorgia arborea*). Despite the widespread occurrence of these two groups of VME indicators in the North Atlantic (FAO, 2019), they are expected to respond differently to future water mass conditions properties. The six deep‐sea fish species selected were the commercially harvested round nose grenadier (*Coryphaenoides rupestris*), Atlantic cod (*Gadus morhua*), blackbelly rosefish (*Helicolenus dactylopterus*), American plaice (*Hippoglossoides platessoides*), Greenland halibut (*Reinhardtius hippoglossoides*) and beaked redfish (*Sebastes mentella*). Although many of these fishes are not strictly considered deep‐sea species, they all occur beyond 200 m depth (Table 1) and are relevant to deep‐sea fisheries including in areas beyond national jurisdictions.

**Table 1:** Number of grid cells with occurrence data obtained from multiple sources for the six cold‐water corals and six deep‐sea fishes used to model the suitable habitat in the North Atlantic. Depth ranges of occurrence records, and mean and standard deviation (±) for slope, Bathymetric Position Index (BPI), temperature at seafloor (Temp), POC flux to seafloor, and Aragonite (Ωar) and Calcite (Ωcal) saturation state at seafloor are also shown. Depth ranges for scleractinian corals and octocorals are shown for reference purposes only since depth was not considered for these species

Group	Species	No. cells	Depth range (m)	Slope (°)	BPI	Temp (°C)	POC flux (mg C m^−2^ day^−1^)	Ωar	Ωcal
Scleractinian corals	*Lophelia pertusa*	1,311	20–2,840	1.55 ± 2.01	0.73 ± 1.06	8.24 ± 2.57	14.78 ± 8.31	1.99 ± 0.30	–
	*Madrepora oculata*	418	100–2,120	2.23 ± 2.41	0.88 ± 1.79	9.41 ± 2.93	9.94 ± 6.48	1.96 ± 0.45	–
	*Desmophyllum dianthus*	312	50–3,250	3.67 ± 3.32	0.80 ± 1.79	8.28 ± 3.17	9.18 ± 5.84	1.81 ± 0.49	–
Octocorals	*Acanthogorgia armata*	324	30–2,600	2.81 ± 2.18	0.78 ± 0.99	4.92 ± 2.21	15.24 ± 6.17	–	2.59 ± 0.30
	*Acanella arbuscula*	852	50–4,810	2.52 ± 2.41	0.75 ± 1.44	4.71 ± 1.99	12.00 ± 5.54	–	2.54 ± 0.28
	*Paragorgia arborea*	434	40–2,170	1.26 ± 1.78	0.52 ± 0.71	3.87 ± 2.26	21.35 ± 8.36	–	2.69 ± 0.21
Deep‐water fish	*Helicolenus dactylopterus*	4,508	20–1,790	1.49 ± 2.23	1.08 ± 1.19	8.59 ± 1.79	24.08 ± 16.35	–	–
	*Sebastes mentella*	15,476	10–1,630	0.80 ± 1.15	0.44 ± 0.74	3.71 ± 1.70	21.08 ± 7.08	–	–
	*Gadus morhua*	52,463	10–990	0.29 ± 0.57	0.22 ± 0.52	5.64 ± 2.75	34.12 ± 14.97	–	–
	*Hippoglossoides platessoides*	56,734	10–1,490	0.34 ± 0.63	0.23 ± 0.54	5.21 ± 2.69	32.19 ± 14.66	–	–
	*Reinhardtius hippoglossoides*	23,491	10–1,690	0.74 ± 1.09	0.30 ± 0.67	3.58 ± 1.89	23.43 ± 9.84	–	–
	*Coryphaenoides rupestris*	3,009	70–1,800	2.23 ± 1.60	0.44 ± 0.88	4.55 ± 1.53	15.16 ± 5.73	–	–

Georeferenced presence‐only records were obtained from institutional databases of partners participating in this work (see Supporting Information Appendix S1, Table S1) as well as from public databases (Table 1) such as the Ocean Biogeographic Information System portal2
https://obis.org/
 (OBIS), the NOAA Deep Sea Coral Data Portal,3
https://deepseacoraldata.noaa.gov/
 and the ICES Vulnerable Marine Ecosystems data portal.4
http://www.ices.dk/marine-data/data-portals/Pages/vulnerable-marine-ecosystems.aspx
 In order to reduce potential errors in the spatial position of the occurrence records, we compared the depth values given by OBIS and NOAA with depth values extracted from the depth raster layer, excluding any occurrence records with no depth information or with depths that differed more than 30% and more than 50 m in absolute depth. In the case of the ICES VMEs database, those records with a position accuracy lower than 5000 m of linear distance were excluded. Species occurrence provided directly by co‐authors was cross‐checked for accuracy of reported depth prior to submission of the data and were considered accurate, and took priority over OBIS data from the same institutional sources. Maps of the presence records used in the models are provided as supporting information (Figure S1).

Most HSM approaches require information on the location of both species presence and absence. However, existing biological datasets rarely include information on species absence, despite its importance for model performance and precision (Iturbide, Bedia, & Gutiérrez, 2018; Iturbide et al., 2015; Wisz & Guisan, 2009). To overcome this obstacle, we generated pseudo‐absence data (a.k.a. background points) by adapting the methodology in Iturbide et al. (2015) to our specific data. In the first step, we used the function *OCSVMprofiling* from the R package MOPA (Iturbide et al., 2018) to limit the geographic region for pseudo‐absence data generation using environmental profiling based on presence data. In the second step, pseudo‐absence data were randomly generated in the region defined above using the function *pseudoAbsences* from the MOPA package, but excluding a buffer distance to presence records of 6 km. The number of pseudo‐absence data points generated differed between cold‐water corals (10,000) and deep‐sea fishes (100,000) because of the different numbers of presence records. Finally, the pseudo‐absence data were randomly stratified subsampled by depth strata to match the proportion of the presence records distribution by depth of all cold‐water corals and fish species in public databases.

### 2.3 Environmental layers

A set of terrain (static in time; Wilson, O’Connell, Brown, Guinan, & Grehan, 2007) and environmental (dynamic in time) variables were used as candidate predictors of present‐day (1951–2000) distribution and to project future (2081–2100) changes. All predictor variables were projected with the Albers equal‐area conical projection centred in the middle of the study area, and were rescaled to a final grid cell resolution of 3x3 km; comprising about 3.8 million cells. The terrain variable depth was extracted from a bathymetry grid built from two data sources: the EMODnet Digital Terrain Model (EMODnet, 2018) and the General Bathymetric Chart of the Oceans (GEBCO 2014 described in Weatherall et al., 2015). The original resolution from EMODNET (0.002°) was rescaled to match the GEBCO resolution (0.008°) using a bilinear interpolation. The bathymetry layer consisted of EMODnet data, where available, merged with GEBCO 2014 and rescaled to the final resolution of 3x3 km using bilinear interpolation. Rescaled EMODnet is considered higher in accuracy than GEBCO 2014 at the same resolution because it contains the most recent multibeam coverage for the Northeast Atlantic (EMODnet, 2018; Schmitt, Schaap, Spoelstra, Loubrieu, & Poncelet, 2019). Slope (in degrees) was derived from the final bathymetry grid using the Raster package in R (Hijmans, 2016) and the Bathymetric Position Index (BPI) was computed using the Benthic Terrain Model 3.0 tool (Walbridge, Slocum, Pobuda, & Wright, 2018) in ArcGIS 10.1 with an inner radius of 3 and an outer radius of 25 grid cells. In order to avoid extreme values, BPI was standardized using the *scale* function from the Raster package.

Environmental variables of present‐day and future conditions, including particulate organic carbon (POC) flux at 100 m depth (*epc100*, mg C m^−2^ day^−1^), bottom water dissolved oxygen concentration (µmol/kg), pH and potential temperature (°C) were downloaded from the Earth System Grid Federation (ESGF) Peer‐to‐Peer (P2P) enterprise system.5
https://esgf-node.llnl.gov
 The *epc100* was converted to export POC flux at the seafloor using the Martin curve (Martin, Knauer, Karl, & Broenkow, 1987) following the equation: *epc* = *epc100* ×* *(water depth/export depth)^−0.858^, and setting the export depth to 100 m. Near seafloor aragonite (Ωar) and calcite (Ωcal) saturation were also used as candidate predictors for habitat suitability of cold‐water coral species. These saturation states were computed by dividing the bottom water carbonate ion concentration (mol/m^3^) by the bottom water carbonate ion concentration (mol/m^3^) for seawater in equilibrium with pure aragonite and calcite. Yearly means of these parameters were calculated for the periods 1951–2000 (historical simulation) and 2081–2100 (RCP8.5 or business‐as‐usual scenario) using the average values obtained from the Geophysical Fluid Dynamics Laboratory's ESM 2G model (GFDL‐ESM‐2G; Dunne et al., 2012), the Institut Pierre Simon Laplace's CM6‐MR model (IPSL‐CM5A‐MR; Dufresne et al., 2013) and Max Planck Institute's ESM‐MR model (MPI‐ESM‐MR; Giorgetta et al., 2013) within the Coupled Models Intercomparison Project Phase 5 (CMIP5) for each grid cell of the present study area. CMIP5 environmental variables were available at a 0.5° resolution and rescaled to match the 3 *× *3 km cell size using universal kriging and depth as a covariate. In order to evaluate the performance of the 1951–2000 seabed environmental layer, we compared modelled data against field observations. In general, modelled values correlated highly significantly with observed data, yielding adjusted *R *> .70 and low values of RMSE (Figure S2). While these results indicate appropriate environmental layers in this study, environmental model projections contain a degree of uncertainty likely associated with the spatiotemporal scales used. Environmental layers used in this study are available to download https://doi.org/10.1594/PANGAEA.911117 and provided as Supporting Information (Figure S3).

Collinearity between all candidate predictor variables was evaluated using Spearman's coefficient of correlation and the variation inflation factor (VIF, Zuur, Ieno, & Elphick, 2010). The final selection of predictor variables was based on the inferred ecological relevance of each variable for each group of species modelled (Fourcade, Besnard, & Secondi, 2018), and therefore differed among scleractinians, octocorals and fish species. Because scleractinian and octocoral species differ in environmental requirements based on how they incorporate calcium carbonate into their skeletons (Freiwald, 2002; Lewis, Barnowski, & Telesnicki, 1992), aragonite saturation was exclusively selected for scleractinians, whereas calcite saturation was selected for octocorals. In order to avoid collinearity, we retained the most ecologically relevant of those variables with Spearman coefficient of correlation >0.85 or with VIF values >10 (Dormann et al., 2013; Elith et al., 2006). After the collinearity analyses, depth (highly correlated with aragonite and calcite saturation), dissolved oxygen concentrations and pH were excluded from the cold‐water coral model development, while excluding only pH from the deep‐water fish model developments. Hence, the variables used for modelling the distribution of scleractinian and octocorals were slope, BPI, POC flux to the seafloor, and bottom water temperature and aragonite or calcite saturation state, whereas fish species distribution modelling included depth, slope, BPI, POC flux to the seafloor, and bottom water dissolved oxygen concentration and temperature.

### 2.4 Modelling approach

We used an ensemble modelling approach to predict habitat suitability under present‐day (1951–2000) conditions and to project changes under future (2081–2100) climate projections (RCP8.5 scenario). An ensemble projecting approach was selected because past studies considered it appropriate to assess uncertainties and enhance reliability in determining suitable habitats under projected climate change scenarios (Araújo & New, 2007; Buisson, Thuiller, Casajus, Lek, & Grenouillet, 2010). We employed three widely used modelling methods (González‐Irusta et al., 2015) capable of dealing with presence‐only data using pseudo‐absences: the maximum entropy model (Maxent, Phillips, Anderson, Dudík, Schapire, & Blair, 2017; Phillips, Anderson, & Schapire, 2006), generalized additive models (GAMs, Hastie & Tibshirani, 1986) and the random forest machine learning algorithms (RF, Breiman, 2001). Maxent models were developed using the function *maxent* from the R package Dismo (Hijmans, Phillips, Leathwick, & Elith, 2017), with prevalence set as the proportion of presences over the pseudo‐absences generated. GAMs were fitted with the function *gam* from the R package mgcv (Wood, 2018) using a binomial error distribution with logit link function, constraining the smooth curves to four knots to avoid overfitting; we used three knots for temperature and aragonite in cold‐water coral models. Finally, we computed RF models using the function *randomForest* from the R package of the same name (Liaw & Wiener, 2002).

Variable selection in GAMs used the Akaike information criterion and the function *dredge* from the R package MuMIn (Barton, 2018), whereas the other two models (Maxent and Random Forest) were fitted with the original set of variables. To assess the contribution of each variable to the final predictions, we used a randomization procedure adapted from Thuiller, Lafourcade, Engler, and Araújo (2009), which estimates the importance of each variable independently of the modelling technique, enabling direct comparisons among models. This methodology computes the Pearson correlation between the original predictions and predictions where the variable under evaluation was randomly permutated. This operation was repeated 10 times for each combination of model, variable and species. We report results as 1‐Pearson correlation in order to provide intuitive values of variable importance, where high values indicate high variable importance. The relationship between the environmental predictors and predicted habitat suitability was analysed using response curves, described by Elith et al. (2006).

Performance for the present‐day model was evaluated using a cross‐validation method based on a random ‘block’ selection of training and testing data (Guinotte & Davies, 2014). Past work suggests this method of partitioning a unique dataset provides the best spatial independence between training and testing datasets (Fourcade et al., 2018). We implemented this methodology with the *get.block* function from the EnMEVAL package in R (Muscarella et al., 2014), dividing the study area into four subareas containing similar numbers of occurrence data points. Selection of three of these four subareas provided training datasets, leaving the fourth area as the test dataset. This operation was repeated 10 times with a random selection of evaluation and training areas to compute the mean and the standard deviation of each metric. From each training and test dataset, we randomly selected 80% of the data in each iteration to avoid repeating the exact same selection in different iterations. Analysis of the ability of the training data to predict the test data in each iteration used the evaluation data and five different statistical metrics: area under the curve (AUC) of the receiver operating characteristic, kappa statistic, specificity (a.k.a. true negative rate), sensitivity (a.k.a. true positive rate, or probability of detection) and true skill statistic (TSS). From these analyses, we considered the overall accuracy of the model prediction good (AUC > 0.8; TSS > 0.6), moderate (0.7 ≤ AUC≤0.8; 0.2 ≤ TSS≤0.6) or poor (AUC < 0.7; TSS < 0.2; Mandrekar, 2010). Fielding and Bell (1997) provide a complete description of these statistics.

Each model was then used to predict a relative index of habitat suitability (HSI) across the study area under present‐day (1951–2000) conditions and to project the HSI for the period 2081–2100 by projecting the present‐day niche onto the environmental layers of projected future conditions. The modelled logistic outputs consisted of an HSI that ranked grid cells according to their predicted suitability for a particular species, rather than the probability of presence (Elith et al., 2011; Greathead et al., 2015). Applying threshold values of HSI into predicted/projected suitable and unsuitable areas for both current and future scenarios produced binary habitat suitability maps for each species, employing two thresholds: 10 percentile training presence logistic threshold and maximum sensitivity and specificity. We computed the uncertainty for each model prediction by bootstrapping the data with replacement (Anderson et al., 2016) using the function boot from the R package boot (Canty & Ripley, 2017), fitting new models using the bootstrapped data and predicting HSI values for the whole study area. Repeating this process 100 times for each model yielded 100 estimates of HSI for each cell. Finally, we computed the coefficient of variation (CV) of the bootstrap output for each species, modelling approach, and the present‐day predictions and future projections.

Finally, ensemble HSI and uncertainty were computed for all species and for the two study periods by calculating the average of these two indexes by cell after weighting the three families of model outputs with the evaluation metrics AUC and TSS, using the same approach as Rowden et al. (2017). We estimated the importance of each predictor variable to the ensemble habitat suitability model predictions as the average of the variable importance in the individual models weighted by the models' evaluation metrics. Ensemble model performance statistic calculations used the same methods as the individual models.

The binary maps were used to calculate the suitable habitat area, along with the median latitude and depth for all species in the North Atlantic, excluding the Mediterranean Sea. We inferred refugia areas (sensu Keppel & Wardell‐Johnson, 2012) for VMEs and local fish stocks of commercially important deep‐sea species in the North Atlantic Ocean from those suitable areas predicted under present‐day and projected under future conditions.

## 3 RESULTS

In general, the three families of modelling approaches (Table S2) and the ensemble model predictions (Table 2) achieved good accuracy for most species (AUC > 0.80 and TSS > 0.60), reasonably matching known species occurrences (sensitivity > 0.80). Ensemble models for all cold‐water coral species also achieved good accuracy, although models for scleractinian species performed slightly better than those for octocoral species. Of those corals examined, the predicted distribution of *D. dianthus* (AUC = 0.95; TSS > 0.74) and *P. arborea* (AUC = 0.95; TSS > 0.76) were most accurate, with lowest accuracy in *A. arbuscula* (AUC = 0.88; TSS < 0.67). Deep‐sea fish model prediction accuracies were slightly better compared to cold‐water corals but related inversely to sample size (Table 2). The predicted distribution of *C. rupestris* (AUC = 0.99; TSS > 0.88; *n* = 3,009) and *H. dactylopterus* (AUC = 0.97; TSS > 0.81; *n* = 4,508) were highest in accuracy among all fishes, in contrast to comparatively low accuracy in *G. morhua* (AUC = 0.94; TSS < 0.81; *n* = 52,463) and *H. platessoides* (AUC = 0.93; TSS < 0.81, *n* = 54,725), and the lowest accuracy in *R. hippoglossoides* (AUC = 0.87; TSS < 0.61, *n* = 23,491). The uncertainty associated with the habitat suitability ensemble model predictions under present‐day and projections under future environmental conditions were generally low for both cold‐water corals and deep‐sea fishes, but generally lower for fishes (Figure S4a,b). Supporting information provided the predicted habitat suitability indices and coefficients of variance for all three families of models and species (Figure S5). Model outputs are available for download from https://doi.org/10.1594/PANGAEA.910319.

**Table 2:** Model performance statistics generated using an ensemble modelling approach. The ability of the training data to predict the probability of presence was tested with different statistical metrics: area under the curve (AUC) of the receiver operating characteristic, kappa statistic, sensitivity (% true positives), specificity (% true negatives) and true skill statistic (TSS). Statistics were calculated using two thresholds: 10 percentile training presence logistic threshold (10th) and maximum sensitivity and specificity (MSS)

Group	Species	AUC	Kappa		Sensitivity		Specificity		TSS		Thresholds	
			10th	MSS	10th	MSS	10th	MSS	10th	MSS	10th	MSS
Scleractinian corals	*Lophelia pertusa*	0.91 ± 0.08	0.51 ± 0.31	0.57 ± 0.25	0.90	0.88 ± 0.06	0.78 ± 0.18	0.85 ± 0.11	0.68 ± 0.18	0.72 ± 0.17	0.34	0.24
	*Madrepora oculata*	0.92 ± 0.06	0.31 ± 0.22	0.41 ± 0.19	0.90	0.88 ± 0.09	0.77 ± 0.16	0.87 ± 0.07	0.66 ± 0.16	0.75 ± 0.14	0.31	0.20
	*Desmophyllum dianthus*	0.95 ± 0.03	0.34 ± 0.14	0.39 ± 0.16	0.90	0.92 ± 0.07	0.85 ± 0.08	0.86 ± 0.09	0.74 ± 0.08	0.79 ± 0.08	0.33	0.22
Octocorals	*Acanthogorgia armata*	0.92 ± 0.05	0.35 ± 0.29	0.43 ± 0.21	0.90	0.88 ± 0.06	0.77 ± 0.20	0.89 ± 0.07	0.66 ± 0.20	0.77 ± 0.12	0.26	0.18
	*Acanella arbuscula*	0.88 ± 0.03	0.22 ± 0.20	0.49 ± 0.20	0.90	0.81 ± 0.07	0.60 ± 0.15	0.86 ± 0.10	0.50 ± 0.15	0.67 ± 0.10	0.32	0.19
	*Paragorgia arborea*	0.95 ± 0.05	0.44 ± 0.18	0.50 ± 0.17	0.90	0.90 ± 0.06	0.86 ± 0.13	0.90 ± 0.09	0.76 ± 0.13	0.79 ± 0.12	0.36	0.23
Deep‐water fish	*Helicolenus dactylopterus*	0.97 ± 0.03	0.81 ± 0.08	0.84 ± 0.08	0.90	0.96 ± 0.02	0.91 ± 0.08	0.88 ± 0.08	0.81 ± 0.08	0.84 ± 0.08	0.54	0.33
	*Sebastes mentella*	0.94 ± 0.06	0.68 ± 0.22	0.67 ± 0.21	0.90	0.95 ± 0.03	0.85 ± 0.15	0.82 ± 0.15	0.75 ± 0.15	0.78 ± 0.13	0.63	0.50
	*Gadus morhua*	0.94 ± 0.02	0.75 ± 0.01	0.79 ± 0.01	0.90	0.99 ± 0.01	0.86 ± 0.01	0.82 ± 0.02	0.76 ± 0.01	0.81 ± 0.01	0.74	0.60
	*Hippoglossoides platessoides*	0.93 ± 0.01	0.75 ± 0.00	0.80 ± 0.03	0.90	0.97 ± 0.04	0.86 ± 0.00	0.84 ± 0.01	0.76 ± 0.00	0.81 ± 0.04	0.73	0.59
	*Reinhardtius hippoglossoides*	0.87 ± 0.01	0.48 ± 0.05	0.52 ± 0.01	0.90	0.90 ± 0.08	0.68 ± 0.05	0.71 ± 0.06	0.58 ± 0.05	0.61 ± 0.02	0.61	0.54
	*Coryphaenoides rupestris*	0.99 ± 0.01	0.88 ± 0.01	0.93 ± 0.03	0.90	0.97 ± 0.02	0.98 ± 0.01	0.96 ± 0.01	0.88 ± 0.01	0.93 ± 0.03	0.63	0.26

The habitat suitability models developed here included seven different predictors which contributed differently to the different modelled species (Table 3; Table S4). In general, POC flux to the seafloor, bottom water temperature, and aragonite or calcite saturation were the most important predictors for scleractinians and octocorals, in contrast to depth, POC flux, and temperature for deep‐sea fishes (Table 3). We also note, however, the importance of slope in predicting the suitable habitat for *D. dianthus*, *A. armata*, and *M. oculata*. Supporting information provides the response curves derived from all modelling approaches (Figures S6 and S7).

**Table 3:** Importance of each predictor variable to the habitat suitability model predictions, measured as 1‐Pearson correlation, for six cold‐water corals and six deep‐sea fishes in the North Atlantic Ocean. Predictor variables were depth, slope, Bathymetric Position Index (BPI), temperature at seafloor (Temp), particulate organic carbon flux to seafloor (POC), aragonite (Ωar) and calcite (Ωcal) saturation state at seafloor, and dissolved oxygen (DO) concentration at seafloor

Group	Species	Relative importance							
		Depth	Slope	BPI	Temp	POC	Ωar	Ωcal	DO
Scleractinian corals	*Lophelia pertusa*	–	0.06 ± 0.04	0.05 ± 0.06	0.41 ± 0.13	0.26 ± 0.10	0.36 ± 0.04	–	
	*Madrepora oculata*	–	0.16 ± 0.09	0.07 ± 0.05	0.47 ± 0.14	0.40 ± 0.06	0.14 ± 0.09	–	
	*Desmophyllum dianthus*	–	0.27 ± 0.13	0.05 ± 0.04	0.44 ± 0.15	0.48 ± 0.20	0.11 ± 0.13	–	
Octocorals	*Acanthogorgia armata*	–	0.19 ± 0.12	0.05 ± 0.05	0.15 ± 0.05	0.29 ± 0.20	–	0.35 ± 0.12	
	*Acanella arbuscula*	–	0.09 ± 0.06	0.03 ± 0.04	0.17 ± 0.08	0.21 ± 0.15	–	0.41 ± 0.06	
	*Paragorgia arborea*	–	0.06 ± 0.05	0.08 ± 0.05	0.15 ± 0.09	0.35 ± 0.27	–	0.58 ± 0.05	
Deep‐water fish	*Helicolenus dactylopterus*	0.64 ± 0.26	0.17 ± 0.22	0.19 ± 0.21	0.45 ± 0.03	0.24 ± 0.17	–	–	0.16 ± 0.16
	*Sebastes mentella*	0.67 ± 0.22	0.17 ± 0.23	0.17 ± 0.22	0.38 ± 0.11	0.43 ± 0.17	–	–	0.13 ± 0.16
	*Gadus morhua*	0.70 ± 0.15	0.15 ± 0.17	0.14 ± 0.17	0.27 ± 0.13	0.40 ± 0.09	–	–	0.31 ± 0.25
	*Hippoglossoides platessoides*	0.64 ± 0.11	0.12 ± 0.14	0.11 ± 0.13	0.29 ± 0.12	0.41 ± 0.12	–	–	0.35 ± 0.13
	*Reinhardtius hippoglossoides*	0.64 ± 0.26	0.22 ± 0.30	0.23 ± 0.30	0.48 ± 0.14	0.50 ± 0.16	–	–	0.27 ± 0.17
	*Coryphaenoides rupestris*	0.67 ± 0.16	0.21 ± 0.19	0.16 ± 0.22	0.20 ± 0.20	0.41 ± 0.10	–	–	0.25 ± 0.17

**Figure 1:**
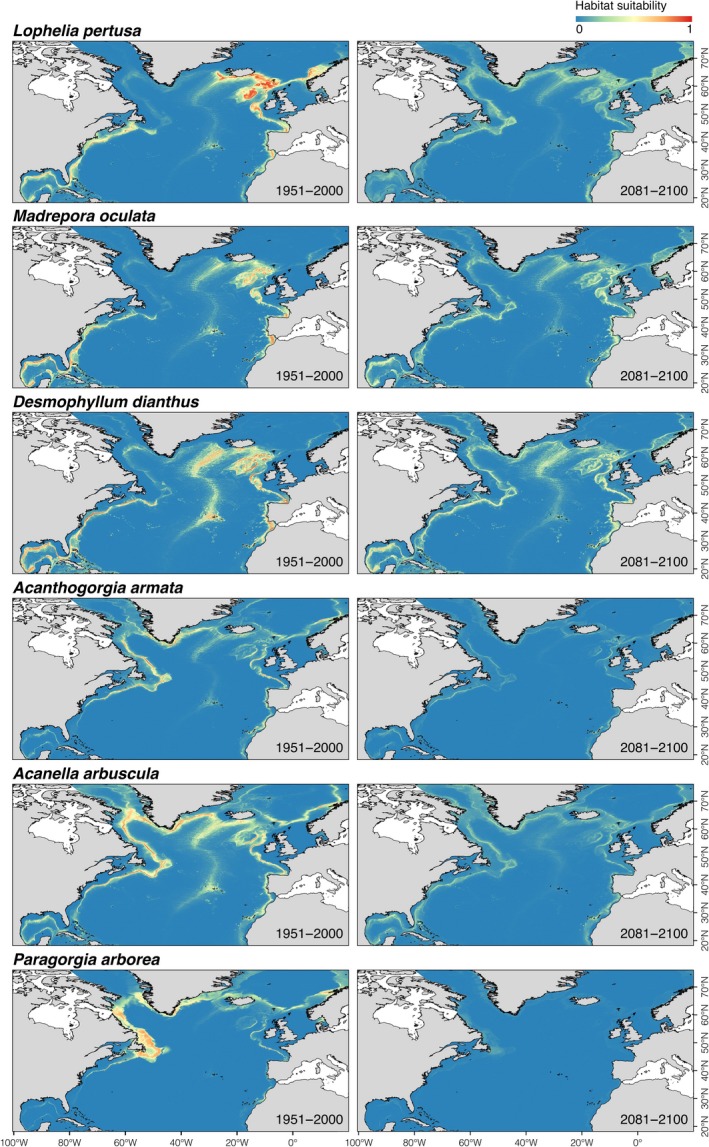
Habitat suitability index predicted under present‐day (1951–2000) and projected under future (2081–2100; RCP8.5 or business‐as‐usual scenario) environmental conditions for cold‐water corals in the North Atlantic Ocean using an ensemble modelling approach

**Figure 2:**
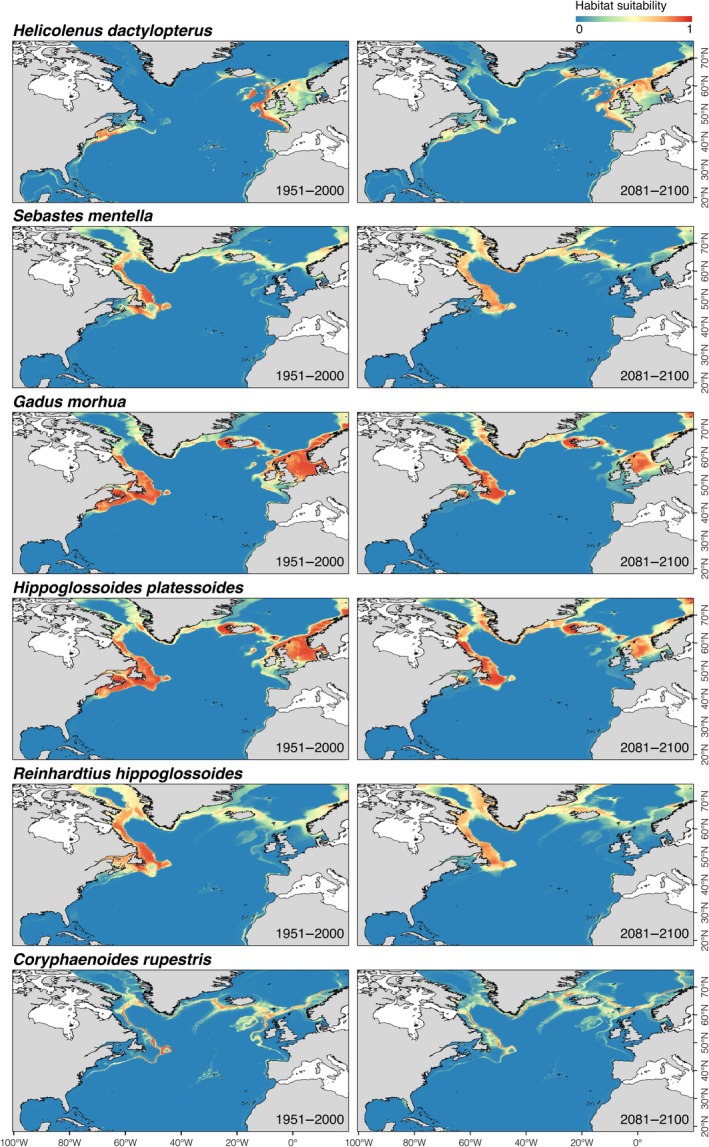
Habitat suitability index predicted under present‐day (1951–2000) and projected under future (2081–2100; RCP8.5 or business‐as‐usual scenario) environmental conditions for commercially important deep‐sea fishes in the North Atlantic Ocean, using an ensemble modelling approach

Patterns in suitable habitat under future climate conditions differed for the different VME indicator species (Figure 1) and deep‐sea fish species evaluated (Figure 2). In general, the model outputs for scleractinian corals and octocorals decreased markedly in the suitable habitat towards the year 2100, whereas deep‐sea fishes clearly shifted towards higher latitudes.

Potential habitat for scleractinian corals under present‐day conditions showed higher suitability indices in the eastern North Atlantic and the Mid‐Atlantic Ridge including the Azores, but also in the Gulf of Mexico, in contrast to higher suitability indices for octocorals in the western North Atlantic and south of Greenland (Figure 1). Most modelled deep‐sea fishes spanned a wide geographic range, with suitable habitats on the continental shelf and slope on both sides of the North Atlantic and along the coast of Iceland and Greenland (Figure 2). However, the predicted suitable habitat for *H. dactylopterus* was limited to areas south of Nova Scotia, south of Iceland, the Azores and around the British Isles, in contrast to deeper areas along the continental slope on both sides of the North Atlantic for *C. rupestris*.

**Figure 3:**
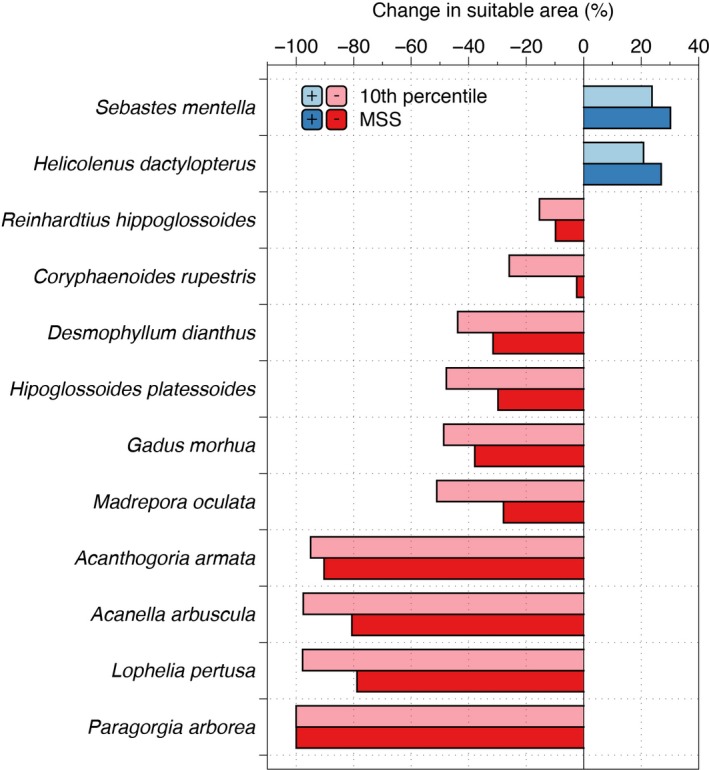
Climate‐induced projected changes (RCP8.5 or business‐as‐usual scenario) in the suitable habitat for cold water corals and deep‐sea fishes in the North Atlantic Ocean with an ensemble modelling approach. The extension of the habitat was calculated from binary maps built with two thresholds: 10 percentile training presence logistic (10th percentile) and maximum sensitivity and specificity (MSS)

**Figure 4:**
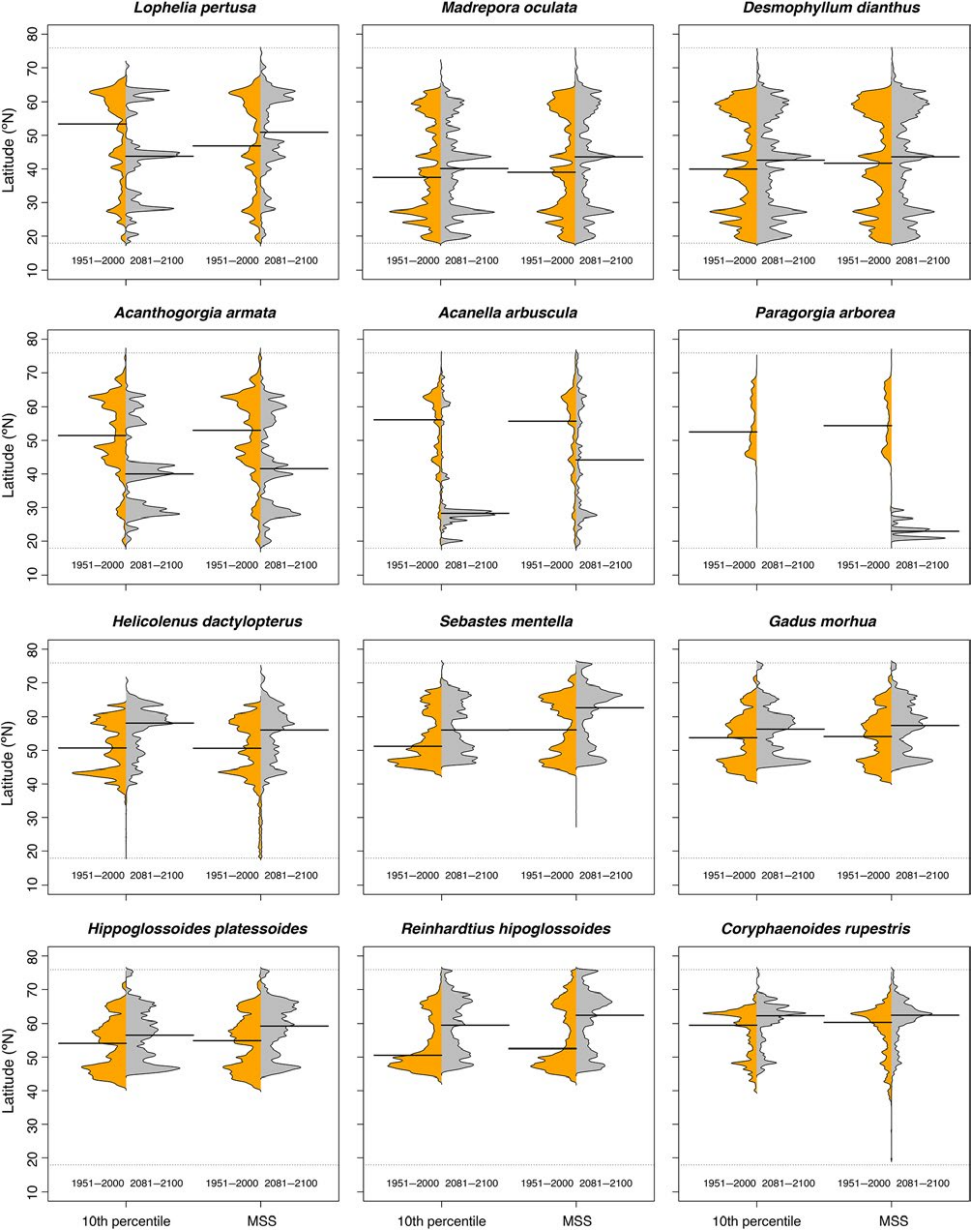
Climate‐induced projected changes (RCP8.5 or business‐as‐usual scenario) in the latitudinal distribution of cold water corals and deep‐sea fishes in the North Atlantic Ocean with an ensemble modelling approach. The extension of the habitat was calculated from binary maps built with two thresholds: 10 percentile training presence logistic (10th percentile) and maximum sensitivity and specificity (MSS). The black line indicates the median latitudinal

**Figure 5:**
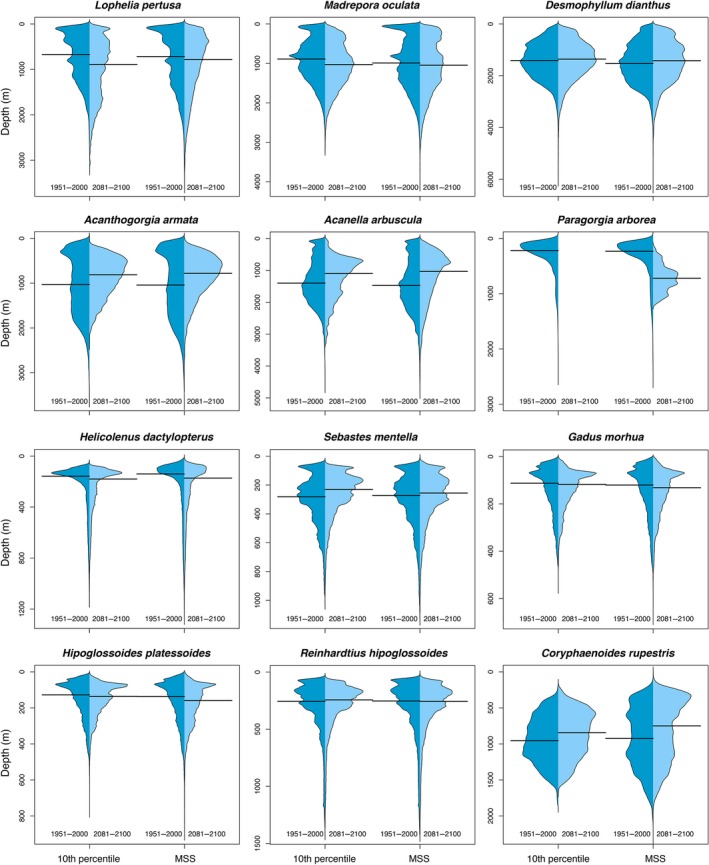
Climate‐induced projected changes (RCP8.5 or business‐as‐usual scenario) in the depth distribution of cold water corals and deep‐sea fishes in the North Atlantic Ocean with an ensemble modelling approach. The extension of the habitat was calculated from binary maps built with two thresholds: 10 percentile training presence logistic (10th percentile) and maximum sensitivity and specificity (MSS). The black line indicates the median depth

**Figure 6:**
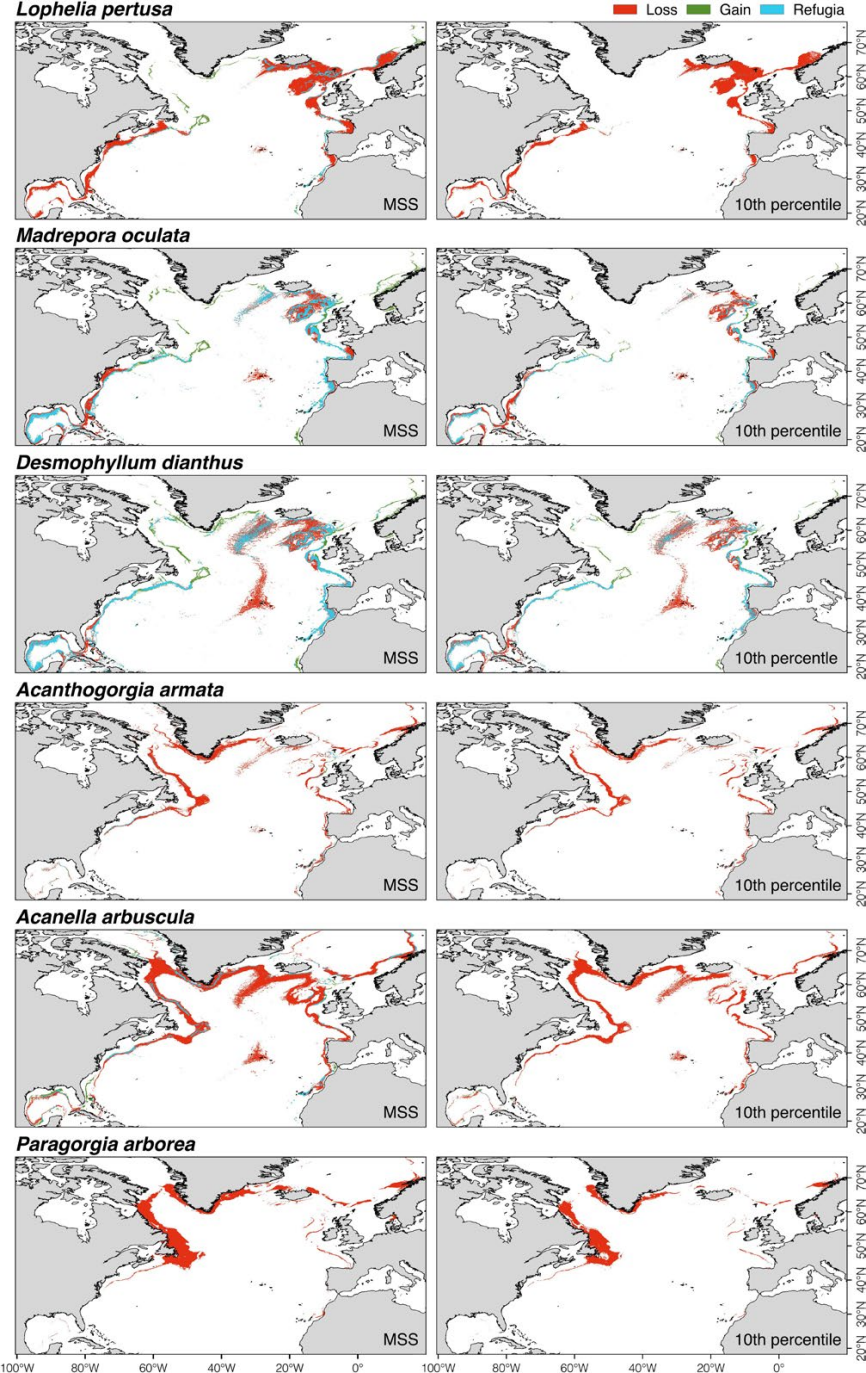
Projected present‐day suitable habitat loss, gain, and acting as climate refugia areas (sensu Keppel & Wardell‐Johnson, 2012) under future (2081–2100; RCP8.5 or business‐as‐usual scenario) environmental conditions for cold‐water corals in the North Atlantic Ocean. Areas were identified from binary maps built with an ensemble modelling approach and two thresholds: 10 percentile training presence logistic threshold (10th) and maximum sensitivity and specificity (MSS)

Based on the ensemble model outputs, the scleractinian *L. pertusa* may experience a major reduction in suitable habitat of over 79% (Figure 3; Figure S8a,b). We observed no clear shift in the median latitudinal distribution of this coral species by 2100 (Figure 4) but we projected a shift in the median depth distribution towards deeper waters (Figure 5) resulting from loss of suitable habitat at shallower depths. The other two scleractinians (*M. oculata* and *D. dianthus*) may experience moderate reduction in suitable habitat of about 30%–55% (Figure 3; Figure S8a,b), with a northern shift in median latitudinal distributions (ranging from 1.9° to 4.6° in latitude; Figure 4) and a shift of *M. oculata* median suitable depths towards deeper waters (Figure 5). The models projected increased suitable habitat for all scleractinian corals not only in the Davis Strait and Labrador Sea, with corresponding decreases in most southern parts of the North Atlantic from the Gulf of Mexico to Nova Scotia, but also in the Mid‐Atlantic Ridge, Bay of Biscay, and in the Rockall and Faroe Shetland areas (Figure 6; Figure S8a,b). Projected climate refugia for scleractinian corals averaged 30%–42% of North Atlantic present‐day habitat on average, depending on the threshold considered (Table 4). However, refugia for *L. pertusa* estimated with the 10th percentile threshold was only about 1.5% of the North Atlantic present‐day habitat (Table 4). Projections indicated climate refugia for scleractinian corals on both sides of the North Atlantic (Figure 6).

**Table 4:** Proportion of the present‐day suitable habitat acting as refugia areas (Keppel & Wardell, 2012) under future (2081–2100; RCP8.5 or business‐as‐usual scenario) environmental conditions for cold‐water corals and commercially important deep‐sea fishes in the North Atlantic Ocean. Refugia areas were identified from binary maps built with an ensemble modelling approach and two thresholds: 10 percentile training presence logistic threshold (10th) and maximum sensitivity and specificity (MSS). Refugia areas estimated by the different models used to calculate the ensemble outputs are also shown: generalized additive model (GAM), maximum entropy model (Maxent) and random forest (RF)

Group	Species	Refugia areas (% present‐day habitat)							
		Ensemble		GAM		Maxent		RF	
		10th	MSS	10th	MSS	10th	MSS	10th	MSS
Scleractinian corals	*Lophelia pertusa*	1.54	13.71	0.25	0.27	41.20	54.93	2.80	16.81
	*Madrepora oculata*	38.98	53.97	27.95	42.30	61.50	73.52	12.81	45.95
	*Desmophyllum dianthus*	45.55	53.58	48.48	47.80	60.80	68.72	17.92	37.95
Octocorals	*Acanthogorgia armata*	4.02	8.13	9.16	12.07	4.76	6.90	4.96	12.67
	*Acanella arbuscula*	1.30	13.85	0.36	1.80	11.14	25.65	11.94	31.52
	*Paragorgia arborea*	0.00	0.00	0.00	0.00	0.11	0.23	0.00	9.99
Deep‐water fish	*Helicolenus dactylopterus*	67.95	84.22	72.72	89.38	85.15	91.64	46.16	63.82
	*Sebastes mentella*	55.92	64.78	51.05	61.59	53.05	70.24	48.04	64.22
	*Gadus morhua*	42.62	51.73	52.10	56.45	12.33	45.84	39.77	47.96
	*Hippoglossoides platessoides*	42.61	55.79	54.97	59.75	13.83	53.70	37.84	48.22
	*Reinhardtius hippoglossoides*	51.54	60.86	50.68	55.68	50.76	57.79	43.81	50.20
	*Coryphaenoides rupestris*	43.48	62.45	52.55	59.63	44.47	68.65	28.52	57.88

Climate change impacts may threaten the persistence of all three octocorals (*A. arbuscula*, *A. armata* and *P. arborea*) in the North Atlantic. The ensemble model outputs suggest a reduction in suitable habitat of over 80% in all regions, irrespective of modelling approach or threshold (Figure 3; Figure S8a,b). The large reduction in suitable habitat resulted in a projected marked southern shift in median latitudinal distribution (−11.3° to −27.8°; Figure 4) and a shift towards shallower depths for *A. arbuscula* and *A. armata *(Figure 5). Our models projected new suitable habitat becoming available for these two species by 2100 in the shallower waters of the northernmost of the North Atlantic and Gulf of Mexico (Figure 6). Extremely small refugia may remain in the North Atlantic for octocorals *A. arbuscula* and *A. armata* (averaging 6%–14% of present‐day suitable habitat), whereas the ensemble model projects no climate refugia for *P. arborea* by 2100 (Table 4; Figure 6).

**Figure 7:**
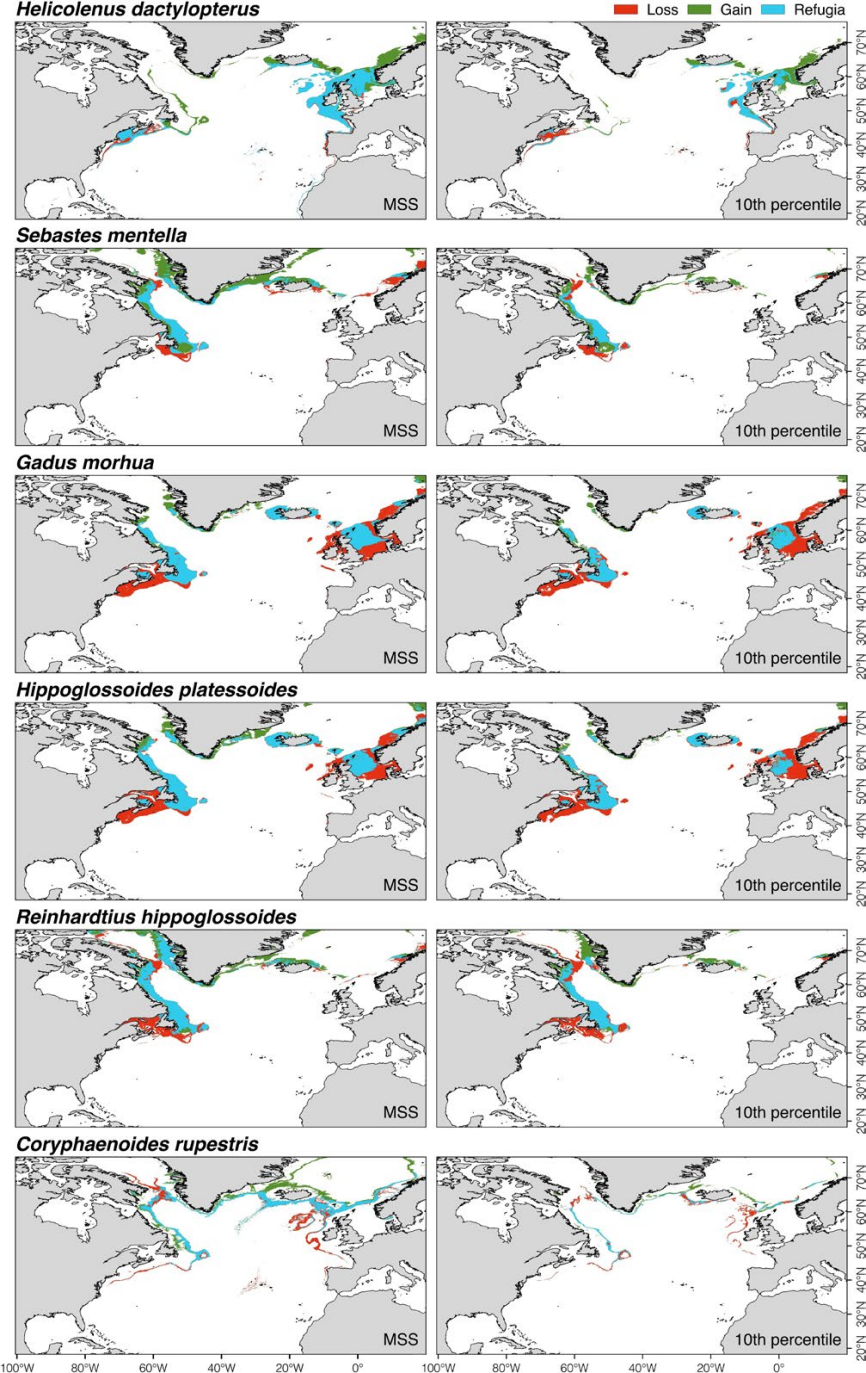
Projected present‐day suitable habitat loss, gain, and acting as climate refugia areas (sensu Keppel & Wardell‐Johnson, 2012) under future (2081–2100; RCP8.5 or business‐as‐usual scenario) environmental conditions for commercially important deep‐sea fishes in the North Atlantic Ocean. Areas were identified from binary maps built with an ensemble modelling approach and two thresholds: 10 percentile training presence logistic threshold (10th) and maximum sensitivity and specificity (MSS)

The ensemble model projected a 30%–50% reduction of suitable habitat for the fish species *G. morhua* and *Hipoglossoides platessoides*, between 10% and 15% for *R. hippoglossoides*, and between 2% and 25% for *C. rupestris,* mostly in their lower latitudinal limit (Figure 3; Figure S9a,b). The decrease in suitable habitat for *G. morhua* included important shallower water fishing grounds, such as Georges Bank, the Irish Sea, the Norwegian Sea and the southern North Sea (Figure S9a,b). Of those species examined, only *H. dactylopterus* and *S. mentella* increased in projected total suitable habitat by 2100 (by about 20%–30%), mostly by expanding their northern latitudinal limit (Figure 3; Figure S9a,b). Therefore, we observed a clear northern shift in the median latitude of suitable habitat for most fishes by 2100 (from 2.0° to 9.9°; Figure 4), but with no clear trend in depth distribution (Figure 5). Projected climate refugia for deep‐water fishes were comparatively large compared to corals, averaging between 51% and 63% of present‐day habitat, depending on the threshold used (Table 4). These projected climate refugia occur mostly on both sides of the northern part of the North Atlantic (Figure 7).

Our results strongly suggest that warming, acidification, and decreasing food availability (Figure S3) will act additively or synergistically to alter the availability of suitable habitat for deep‐sea species (Figures 6 and 7). Our analyses link marked loss of suitable habitat for cold‐water corals to the shoaling of aragonite and calcite saturation horizons as a consequence of ocean acidification in the NE (the Rockall and Faroe Shetland areas) and SE regions of the North Atlantic. This effect will act synergistically with a strong decrease in food availability on the Mid‐Atlantic Ridge. In the NW region of the North Atlantic (Davis Strait and Labrador Sea), reduced suitable habitat for octocorals in deeper waters was linked with the shoaling of the calcite saturation horizon as a consequence of ocean acidification. In the SW region from the Florida Strait to Georges Bank, loss of suitable habitat for scleractinian corals was linked to projected warming of deeper waters, whereas changes for octocorals were linked to shoaling of the calcite saturation horizon. In the Gulf of Mexico, the projected decreases in the suitable habitat of cold‐water corals were associated with anticipated warming of deeper waters. Increasing water temperature in most regions of the North Atlantic and decreasing food availability on the MAR contributed to the marked loss of suitable habitat for deep‐sea fishes. In contrast, warming of shallow waters in Davis Strait and the Labrador Sea and in the NE region resulted in projected suitable habitat gain for both cold‐water corals and fishes, along with suitable habitat gains for corals from the warming of deeper waters in North Atlantic lower latitudes.

## 4 DISCUSSION

Our model predictions and projections showed that North Atlantic deep‐sea species with the best‐studied distributions could experience a significant reduction in suitable habitat by 2100 as a result of climate change. Indeed, our results suggest that the suitable habitat of scleractinian corals in the North Atlantic may be reduced by more than 50%, potentially extirpating all three octocorals studied (*A. arbuscula*, *A. armata* and *P. arborea*). This reduction could be of particular concern for *A. armata*, a species limited in distribution to the North Atlantic Ocean. Our projection also suggested a northward shift of suitable habitat for many commercially important deep‐sea fishes, a finding consistent with the hypothesis of a poleward expansion in response to climate change (Jones et al., 2013; Perry et al., 2005; Poloczanska et al., 2013). Our study projected very limited, discrete climate‐change refugia for cold‐water corals, and especially octocorals, highlighting the need for accurate fine‐scale climate grids and methodologies to properly identify climate refugia (Ashcroft, 2010; Kavousi & Keppel, 2018; Keppel et al., 2012; Valdimarsson, Astthorsson, & Palsson, 2012). Occupancy of the future suitable habitats will depend on connectivity pathways and will differ greatly between deep‐sea fishes which have juvenile and adult mobility and the cold‐water corals which can only disperse as larvae (Andrello, Mouillot, Somot, Thuiller, & Manel, 2015; Baco et al., 2016; Hilário et al., 2015). The latter will be much more dependent on downstream connectivity paths that have not been evaluated in our projections but which are likely to further impose fragmentation of populations through loss of source populations (Fox, Henry, Corne, and Roberts (2016). Fox et al. (2016) have shown that past changes in the NAO significantly altered network connectivity and source–sink dynamics for *L. pertusa* in the northeast Atlantic, and there is every reason to anticipate similar impacts associated with the future climate scenarios shown here.

Previous studies suggested some of the general changes in distribution patterns identified here for both cold‐water corals and deep‐sea fishes, based on inferences about changes in distribution resulting from the likely effects of climate change on the marine environment. Specifically, several studies projected significant loss of suitable habitat for cold‐water coral reefs globally (Guinotte et al., 2006; Tittensor et al., 2010; Zheng & Cao, 2014) or in UK waters (Jackson, Davies, Howell, Kershaw, & Hall‐Spencer, 2014) as a result of ocean acidification. Other studies projected the loss of suitable habitat for Atlantic cod on Georges Bank, and in the Celtic and Irish Seas, a reduction in the southern North Sea and a northward expansion along Greenland and the Davis Strait and the Arctic Ocean (Drinkwater, 2005; Fogarty, Incze, Hayhoe, Mountain, & Manning, 2008; Fossheim et al., 2015).

Furthermore, field surveys already allude to pattern shifts, such as the collapse of Atlantic cod fisheries in the Gulf of Maine as a result of warming (Pershing et al., 2015). Other studies documented the northward movement of blackbelly rosefish and Atlantic cod in the North Sea (Beare et al., 2004; Engelhard, Righton, & Pinnegar, 2014; Perry et al., 2005), increased abundance of blackbelly rosefish in Icelandic waters (Sólmundsson et al., 2019), the northward movement of blackbelly rosefish and a reduction in suitable habitat for American plaice in the western North Atlantic (Nye, Link, Hare, & Overholtz, 2009) and reduced abundance of Greenland halibut in the Barents Sea (Fossheim et al., 2015). Such northward shifting of ranges is already being documented for some western North Atlantic shelf and slope fishes (Møller et al., 2010; Nye et al., 2009), and although Ross, Rhode, Viada, and Mather (2016) also reported northward range extensions, they cautioned that lack of historical baseline surveys limits interpretation of distributional data. Our study presents another example of the potential value of environmental niche modelling for projecting changes in suitable habitat for deep‐sea species under future climate scenarios, and extends the more localized or species‐specific studies already reported above, to much larger spatial scales and more consistent and rigorous analytical methods.

With climate change potentially affecting all species in the ecosystem its influence on community assembly processes remains a major knowledge gap. Field surveys suggest an association between some deep‐sea fishes (e.g. *H. dactylopterus*) and live cold‐water corals reefs (e.g. *L. pertusa*; D'Onghia et al., 2012; Pham et al., 2015). Fish species use such habitats as both spawning and nursery grounds (Corbera et al., 2019), calling into question whether the projected range expansion of the blackbelly rosefish is likely to occur given projected declines of a species forming one of its prime habitats, for example, *L. pertusa*. However, insufficient evidence exists to infer that blackbelly rosefish can occupy transitional and noncoral habitats in many regions (Biber et al., 2014; Milligan, Spence, Roberts, & Bailey, 2016; Ross & Quattrini, 2007), suggesting that declines in scleractinian suitable habitat may not translate into loss of habitat for this deep‐sea fish species.

Temperature and depth were important predictors of habitat suitability for cold‐water coral and deep‐sea fishes respectively. This result corresponds to similar studies on distributions of other deep‐sea corals (e.g. Buhl‐Mortensen, Olafsdottir, Buhl‐Mortensen, Burgos, & Ragnarsson, 2015; Chimienti, Taviani, & Mastrototaro, 2019; Davies & Guinotte, 2011; Georgian, Shedd, & Cordes, 2014; Guinotte & Davies, 2014; Lauria et al., 2017; Tittensor et al., 2010) and deep‐sea fishes (Gomez et al., 2015; Parra et al., 2017; Ross & Quattrini, 2007). However, the strong autocorrelation between depth and temperature and significant correlation with other environmental and biological factors complicates efforts to elucidate the environmental parameters primarily responsible for the observed patterns. Slope and other terrain attributes also help shape distributions of some cold‐water coral species (Rengstorf, Yesson, Brown, & Grehan, 2013) and are linked to the higher suitability of high geomorphological relief habitats that promote stronger near‐bed currents and enhanced food supply (Genin, Dayton, Lonsdale, & Spiess, 1986; Hebbeln, Van Rooij, & Wienberg, 2016; Soetaert Mohn, Rengstorf, Grehan, & Van Oevelen, 2016). Habitat slope and rugosity are also important elements influencing distributions of some deep‐sea fishes (Quattrini, Ross, Carlson, & Nizinski, 2012; Ross & Quattrini, 2009; Ross, Rhode, & Quattrini, 2015). For those fishes intimately tied to complex habitat, loss of corals may tend to disperse (as they search for remaining habitat) or concentrate (as they utilize shrinking habitats) fish communities.

Multiple studies document the importance of aragonite and calcite saturation state in determining cold‐water coral habitat suitability (Davies & Guinotte, 2011; Thresher et al., 2015; Tittensor et al., 2010; Yesson et al., 2012), because waters supersaturated in carbonate enable coral skeleton bio‐calcification. The chemical dissolution and biological erosion of the unprotected skeleton exposed to corrosive waters will impair the long‐term survival of cold‐water coral reefs (Hennige et al., 2015; Schönberg, Fang, Carreiro‐Silva, Tribollet, & Wisshak, 2017; Thresher, Tilbrook, Fallon, Wilson, & Adkins, 2011). However, cold‐water corals may occur in undersaturated waters of high productivity, leading to the hypothesis that increased food supply may compensate to some degree for undersaturation by providing the additional energy necessary to survive (Baco et al., 2017; Ross, unpublished data; Thresher et al., 2011). Elevated food supply may also compensate for low dissolved oxygen concentrations (Hanz et al., 2019). However, in an environment with consistently scarce food or low oxygen concentration, the metabolic costs of calcifying in extremely low carbonate conditions may become prohibitively expensive, thus compromising coral survival (Carreiro‐Silva et al., 2014; Hennige et al., 2015; Maier et al., 2016).

In fact, food availability measured as POC flux to the seafloor was also an important predictor of suitable habitat for most cold‐water corals and deep‐sea fishes in our study. This finding corroborates reports of abundant *L. pertusa* in regions of elevated POC flux, both in recent times (Davies & Guinotte, 2011; White, Mohn, Stigter, & Mottram, 2005) and since the last glacial events (Boavida et al., 2019; Henry et al., 2014; Matos et al., 2015; Wienberg et al., 2010). Indeed, multiple studies link reduced food availability to reduced physiological performance (e.g. calcification and respiratory metabolism) and condition of cold‐water corals (Büscher et al., 2017; Larsson, Lundälv, & van Oevelen, 2013; Naumann, Orejas, Wild, & Ferrier‐Pagès, 2011), as well as their ability to cope with ocean change (Büscher et al., 2017; Georgian et al., 2016; Gomez et al., 2019; Maier et al., 2016; Wood, Spicer, & Widdicombe, 2008). In contrast, the direct link between POC flux and deep‐sea fish abundances has proven difficult to demonstrate (Bailey, Ruhl, & Smith, 2006), despite some evidence that increased surface production may fuel key fish prey taxa such as benthic invertebrates (Bailey et al., 2006; Drazen, Bailey, Ruhl, & Smith, 2012; Ruhl & Smith, 2004). Therefore, the projected decrease in food availability by 2100 in the North Atlantic (Gehlen et al., 2014; Gomez et al., 2019; Sweetman et al., 2017) may exacerbate the likely negative effects of other environmental changes.

Inferring the capacity of deep‐sea species, and corals in particular, to adapt to changes in water chemistry projected by climatic models is challenging. For example, experimental (Keller & Os'kina, 2008) and palaeoecological studies (Wienberg et al., 2010) suggest that some *M. oculata* populations can tolerate elevated seawater temperature; this tolerance may explain their prevalence at shallower depths (180–360 m) in the Mediterranean Sea (Chimienti, Bo, & Mastrototaro, 2018; Chimienti et al., 2019; Freiwald et al., 2009; Gori et al., 2013). *L. pertusa* also may occur in regions (e.g. beneath the Florida Current, Gulf Stream) that experience periodic high temperatures (12–15°C) and rapid water property fluctuations (Brooke, Ross, Bane, Seim, & Young, 2013), but the impact of these conditions is unclear. However, *D. dianthus*, which may also tolerate high temperatures (Naumann, Orejas, & Ferrier‐Pagès, 2013) and survive in waters undersaturated in aragonite (Jantzen et al., 2013; Rodolfo‐Metalpa et al., 2015; Thresher et al., 2011), may experience reduced metabolism that compromise survival when exposed to the combined effects of increased temperature and reduced aragonite saturation (Gori et al., 2016). Additionally, although other scleractinians and octocorals may calcify and grow under low or undersaturated conditions (Büscher et al., 2017; Form & Riebesell, 2012; Hennige et al., 2014, 2015; Maier et al., 2012, 2013; Movilla et al., 2014; Thresher et al., 2011), their capacity to sustain calcification and other physiological processes under unfavourable conditions remains unclear, given studies that show effects of low carbonate concentrations on coral metabolism (Hennige et al., 2014) and increased energy demand required to maintain pH homeostasis at calcification sites (McCulloch et al., 2012; Raybaud et al., 2017). Despite the many uncertainties regarding potential acclimation and adaptation of cold‐water coral species to changes in climate, along with interspecific genetic variability (Kurman et al., 2017) and potential for local adaptation (Georgian et al., 2016), growing evidence points to limited long‐term capacity for adaptation to multiple stressors associated with climate change (Kurman et al., 2017).

Habitat suitability modelling approaches come with some caveats, and we, therefore, acknowledge multiple common and well‐known limitations that may be particularly pronounced when modelling deep‐sea taxa. For example, cold‐water coral and fish distributions respond to small‐scale variation in terrain, such as substrate type and seabed rugosity, as well as local oceanographic conditions such as food availability (Bennecke & Metaxas, 2017; De Clippele et al., 2017; Drazen et al., 2012; Rengstorf et al., 2013; Ross et al., 2015; White et al., 2005). We also recognize some limitations from the quantity, quality and spatial coverage of occurrence data, availability of absence records as well as some uncertainty in deep‐sea species identification (mostly for cold‐water corals). For example, two new species of *Acanella* were recently described from the Gulf of Mexico and Norfolk Canyon off the coast of eastern United States (Saucier, Sajjadi, & France, 2017). We, therefore, cannot state whether previous records from several databases include some of these new species. The deep sea remains one of the least studied and sampled areas on the planet, with many undescribed species and unresolved taxonomy that constrain determination of the full spatial distributions of many species. Extensive exploration of the deep‐sea environment may eventually reduce this uncertainty, but it will take time. Further integration of species‐level biogeochemical and physical data, as well as results of the ecophysiological performance of deep‐sea organisms from ex situ experimental work, will improve suitability and distribution mapping, but noting the need for additional mechanistic (experimentally derived) understanding of how climate drivers elicit ecological responses.

Finally, future climate scenarios always hinge on assumptions; indeed, the RCP8.5 or business‐as‐usual scenario projections for 2081–2100 assume specific future greenhouse gas emissions, world population growth and technology development (Riahi et al., 2011; Van Vuuren et al., 2011), and also encompass climate model errors shared by all scenarios. Consequently, our projected future models for cold‐water coral and fish species potentially represent worst‐case scenarios, with a high degree of uncertainty. Furthermore, future climate projections may not capture localized effects that may influence benthic organisms. Nevertheless, our projections of distribution changes in key species across the North Atlantic offer critical, best available information for decision makers to develop long‐term sustainable management plans (Rheuban, Doney, Cooley, & Hart, 2018), highlighting the utility of enhanced international dialogue on basin‐scale management.

Following sufficient ground‐truthing, habitat suitability models can become valuable tools to inform environmental management and conservation policy (Robinson, Nelson, Costello, Sutherland, & Lundquist, 2017) and provide a basis for taking climate change into consideration (Johnson et al., 2018; Johnson & Kenchington, 2019), as demonstrated here. In practice, these approaches can help put climate change aspects into area‐based management decisions such as those aimed to preserve VMEs (UNGA, 2006), Areas of Particular Environmental Interest designated by the International Seabed Authority, Ecologically and Biologically Significant Areas designated under the Convention on Biological Diversity (CBD), OECMs specified in CBD COP Decision 14/8 (CBD, 2018b; Garcia, Rice, Friedman, & Himes‐Cornell, in press; IUCN WCPA, 2019), or in the new legally binding international instrument for the conservation and sustainable use of biodiversity in areas beyond national jurisdiction (see Wright et al., 2019) which is currently under discussion. Knowledge of changing species distributions can also inform the United Nations Framework Convention on Climate Change.

Habitat suitability projections can help in developing research agendas that confirm and advance the model outputs and clarify the roles of predictor variables in determining species distributions. Incorporating these needs into the Deep Ocean Observing Strategy can help fill data gaps, and prioritize spatial locations for the collection of key physical and biogeochemical data (Canonico et al., 2019; Levin et al., 2019). As forward‐looking international entities, the United Nations’ Decade for Ocean Science, the Global Ocean Observing System, and the Regular Process for Global Reporting and Assessment of the State of the Marine Environment (a.k.a. World Ocean Assessment) can help set such science agendas.

In summary, we have shown that despite all the caveats, habitat suitability models can produce potentially useful projections of future changes in the distribution of deep‐water fish and invertebrate species, and areas where foundation species could be impacted by climate change and may be used to inform management decisions. This application is especially relevant for dramatic changes such as those projected here. Although ocean‐basin scale models provide useful coarse, directional information regarding climate change impacts on deep‐sea fauna, regional models could help to resolve changes of distribution and identification of refugia for monitoring purposes. We hope our study offers a suitable template for, and will stimulate, similar analyses on other taxa or regions.

## Data Availability

The bathymetry data supporting the analyses are publicly available from the EMODnet Digital Terrain Model and the General Bathymetric Chart of the Oceans portals (https://portal.emodnet-bathymetry.eu/ and https://www.gebco.net/data_and_products/gridded_bathymetry_data/, respectively). The environmental data used in this study are publicly available from the Earth System Grid Federation (ESGF) Peer‐to‐Peer (P2P) enterprise system (https://esgf.llnl.gov/). All analyses were conducted using publicly available packages from the Comprehensive R Archive Network (https://cran.r-project.org/), namely the R packages MOPA, Raster, Dismo, mgcv, *randomForest*, MuMIn, EnMEVAL, and boot. The Benthic Terrain Model 3.0 tool is available from the ESRI Oceans GitHub repository (https://github.com/EsriOceans/btm). The processed environmental data layers used in this study are publicly available through the PANGAEA data publisher portal (https://doi.org/10.1594/PANGAEA.911117). The model outputs are also available for download from PANGAEA (https://doi.org/10.1594/PANGAEA.910319).
