# Supplementary material for: Climate‐induced changes in the suitable habitat of cold‐water corals and commercially important deep‐sea fishes in the North Atlantic
Source: Glob Chang Biol. 2020 Feb 20;26(4):2181–202. doi: 10.1111/gcb.14996 (PMC7154791; doi:10.1111/gcb.14996)
Supplement: Supplementary file 1 [file GCB-26-2181-s001.docx]

**Supporting Information**

**Title:** Climate-induced changes in the habitat suitability of cold-water corals and commercially important deep-sea fishes in the North Atlantic

**Authors:** Telmo Morato^1,2^, José-Manuel González-Irusta^1,2^, Carlos Dominguez-Carrió^1,2^, Chih-Lin Wei^3^, Andrew Davies^4^, Andrew K. Sweetman^5^, Gerald H. Taranto^1,2^, Lindsay Beazley^6^, Ana García-Alegre^7^, Anthony Grehan^8^, Pascal Laffargue^9^, Francisco Javier Murillo^6^, Mar Sacau^7^, Sandrine Vaz^10^, Ellen Kenchington^6^, Sophie Arnaud-Haond^10^, Oisín Callery^8^, Giovanni Chimienti^11,12^, Erik Cordes^13^, Hronn Egilsdottir^14^; André Freiwald^15^, Ryan Gasbarro^13^, Cristina Gutierrez-Zárate^1,2^; Matt Gianni^16^, Kent Gilkinson^17^, Vonda E. Wareham Hayes^17^, Dierk Hebbeln^18^, Kevin Hedges^19^, Lea-Anne Henry^20^, David Johnson^21^, Mariano Koen-Alonso^17^, Cam Lirette^6^, Francesco Mastrototaro^11,12^, Lénaick Menot^22^, Tina Molodtsova^23^, Pablo Durán Muñoz^7^, Covadonga Orejas^24^, Maria Grazia Pennino^7^, Patricia Puerta^24^, Stefán Á. Ragnarsson^14^, Berta Ramiro-Sánchez^20^, Jake Rice^25^, Jesús Rivera^26^, Murray Roberts^20^, Steve W. Ross^27^, José L. Rueda^28^, Íris Sampaio^1,2^, Paul Snelgrove^29^, David Stirling^30^, Margaret A. Treble^19^, Javier Urra^28^, Johanne Vad^20^, Dick van Oevelen^31^, Les Watling^32^, Wojciech Walkusz^19^, Claudia Wienberg^18^, Mathieu Woillez^22^, Lisa A. Levin^33^, Marina Carreiro-Silva^1,2^

**Institutional affiliations**:

1. Okeanos Research Centre, Universidade dos Açores, Departamento de Oceanografia e Pesca, 9901-862 Horta, Portugal
2. IMAR Instituto do Mar, Universidade dos Açores, Departamento de Oceanografia e Pesca, 9901-862 Horta, Portugal
3. Institute of Oceanography, National Taiwan University, Taipei, Taiwan
4. Department of Biological Sciences, University of Rhode Island, Kingston, Rhode Island, USA
5. Marine Benthic Ecology, Biogeochemistry and In situ Technology Research Group, The Lyell Centre for Earth and Marine Science and Technology, Heriot-Watt University, Edinburgh, United Kingdom
6. Bedford Institute of Oceanography, Fisheries and Oceans Canada, Dartmouth, NS, Canada
7. Instituto Español de Oceanografía (IEO), Centro Oceanográfico de Vigo, Vigo, Pontevedra, Spain.
8. Earth and Ocean Sciences, NUI Galway, Ireland
9. IFREMER, Centre Atlantique, Nantes, France
10. MARBEC, IFREMER, Univ. Montpellier, IRD, CNRS, France
11. Department of Biology, University of Bari Aldo Moro, Bari, Italy
12. CoNISMa, Rome, Italy
13. Department of Biology, Temple University, Philadelphia, USA
14. Marine and Freshwater Research Institute, Reykjavík, Iceland
15. Senckenberg am Meer, Marine Research Department, Wilhelmshaven, Germany
16. Gianni Consultancy, Amsterdam, Netherlands
17. Northwest Atlantic Fisheries Centre, Fisheries and Ocean Canada, St. John’s, NL, Canada
18. MARUM - Center for Marine Environmental Sciences, University of Bremen, Germany
19. Fisheries and Oceans Canada, Winnipeg, MB, Canada
20. School of GeoSciences, Grant Institute, University of Edinburgh, United Kingdom
21. Seascape Consultants Ltd, Romsey, United Kingdom
22. IFREMER, Centre de Bretagne, Plouzané, France
23. P.P. Shirshov Institute of Oceanology RAS, Moscow, Russia
24. Instituto Español de Oceanografía, Centro Oceanográfico de Baleares, Palma, Spain
25. Fisheries and Ocean Canada, Ottawa, ON, Canada
26. Instituto Español de Oceanografía, Madrid, Spain
27. Center for Marine Science, University of North Carolina at Wilmington, Wilmington, NC, USA
28. Instituto Español de Oceanografía, Centro Oceanográfico de Málaga, Málaga, Spain
29. Ocean Sciences Centre, Memorial University, St. John’s, NL, Canada
30. Marine Laboratory, Marine Scotland Science, Aberdeen, Scotland, UK
31. Royal Netherlands Institute for Sea Research, Yerseke, The Netherlands
32. Department of Biology, University of Hawai‘i at Mānoa, Honolulu, HI, USA
33. Center for Marine Biodiversity and Conservation and Integrative Oceanography Division, Scripps Institution of Oceanography, UC San Diego, La Jolla, CA, USA

**Corresponding author**: +351 292 200 400, [t.morato@gmail.com](mailto:t.morato@gmail.com)

**Supporting Information Appendix S1**

Within the scope the H2020 ATLAS project WP3 (Biodiversity and biogeography), a data call was launched to compile an update geo-referenced dataset for twelve species of cold-water corals and fish to perform the ocean basin scale Habitat Suitability Modelling (Table S1). The VME indicator taxa selected included three scleractinian corals that form aragonite skeletons (*Lophelia pertusa^[[1]](#footnote-1)^*, *Madrepora oculata*, and *Desmophyllum dianthus*), and three octocorals that form calcite skeletons (*Acanella arbuscula*, *Acanthogorgia armata*, and *Paragorgia arborea*). The six deep-sea fish species selected were the commercially harvested roundnose grenadier (*Coryphaenoides rupestris*), Atlantic cod (*Gadus morhua*), blackbelly rosefish (*Helicolenus dactylopterus*), American plaice (*Hippoglossoides platessoides*), Greenland halibut (*Reinhardtius hippoglossoides*), and beaked redfish (*Sebastes mentella*). Partners were asked to share the best available geo-referenced data, including confidential data, and provide with specific acknowledgments to funders, colleagues or others. In order to facilitate the data call, a simple reporting table with the basic information required was prepared. Therefore, georeferenced presence-only records were obtained for twelve species from institutional databases of partners participating in this work (a.k.a the H2020 ATLAS project Database). Here we summarised the occurrence data provided by the different partners and that was used in habitat suitability models.

**Supporting Table S1. Geo-referenced data of key benthic species** provided by partners in this study used in the basin scale habitat suitability models.

| **Data provider** | **VME indicator** | **Fish** | **Total** |
| --- | --- | --- | --- |
| Fisheries and Ocean, Canada (DFO) | 11,911 | 93,359 | 105,270 |
| Marine and Freshwater Research Institute (MFRI) |  | 44,198 | 44,198 |
| Temple University, USA (TU) | 9,873 |  | 9,873 |
| Insituto do Mar, University of the Azores, Portugal (IMAR-UAz) | 184 | 5,447 | 5,631 |
| L'Institut Français de Recherche pour l'Exploitation de la Mer, France (IFREMER) | 3,576 | 1,828 | 5,404 |
| School of GeoSciences, University of Edinburgh, UK (UEDIN) | 3,947 |  | 3,947 |
| Marine Scotland Science, Scotland, UK (MSS) | 715 | 3,047 | 3,762 |
| Instituto Español de Oceanografía, Centro Oceanográfico de Baleares, Spain (IEO – Maiorca) | 1,492 | 235 | 1,727 |
| Instituto Español de Oceanografía, Centro Oceanográfico de Vigo, Spain (IEO – Vigo) | 520 |  | 520 |
| Instituto Español de Oceanografía, Centro Oceanográfico de Málaga, Spain (IEO – Malaga) | 136 | 45 | 181 |
| Department of Biology, University of Bari Aldo Moro, Italy (UOB) | 116 |  | 116 |
| Earth and Ocean Sciences, National University of Ireland, Galway, Ireland (NUIG) | 53 |  | 53 |
| MARUM, Center for Marine Environmental Sciences, University of Bremen, Germany (UNI-HB) | 39 | 14 | 53 |
| Center for Marine Science, University of North Carolina, Wilmington, USA (UNC-W) | 38 | 8 | 46 |
| P.P. Shirshov Institute of Oceanology, Russia (P.P. Shirshov) | 37 |  | 37 |
| **TOTAL** | **32,637** | **148,181** | **180,818** |

**Data provider:** Fisheries and Oceans (DFO), Canada

Fisheries and Oceans (DFO), Canada, conducts annual research vessel bottom trawl surveys on the Atlantic coast of Canada and in the Eastern Canadian Arctic. These surveys share the basic stratified random sampling design, but use different vessels, trawl gears, and differ on the details of their sampling protocols (Chadwick et al., 2007). Fish and cold-water coral data used in this study was obtained from these surveys, and from optical surveys using ROVs and drop cameras. Here we used data from 1995-2017 from the Newfoundland and Labrador region, 2005-2017 from the Eastern Arctic region and 1978-2016 from the Scotian Shelf and Gulf of Maine.

***Acknowledgments***: DFO thanks the Greenland Institute of Natural Resources, Government of Nunauvt, Nunavut Offshore Allocation Holders Association, Nunavut Wildlife Management Board, Nunavut Tunngavik, and Makivik for their roles in the collection of data from the eastern Canadian Arctic.

***References***:

Chadwick, E.M.P., W. Brodie, E. Colbourne, D. Clark, D. Gascon, & T. Hurlbut, 2007. History of annual multi-species trawl surveys on the Atlantic coast of Canada. AZMP Bulletin PMZA 6: 25-42.

DFO Scientific Research (Video) Survey (1999-2003, 2005-2013), Fisheries And Oceans Canada - Maritime Region, Contact: Ellen Kenchington, [Ellen.Kenchington@dfo-mpo.gc.ca](mailto:Ellen.Kenchington@dfo-mpo.gc.ca)

Northern Shrimp Trawl Surveys (2005-2017), Fisheries and Oceans Canada - Central and Arctic Region, Contact: Ellen Kenchington, [Ellen.Kenchington@dfo-mpo.gc.ca](mailto:Ellen.Kenchington@dfo-mpo.gc.ca)

Research Vessel Multispecies Bottom Trawl Surveys (1970-2018), Fisheries and Oceans Canada - Maritimes Region, Contact: Ellen Kenchington, [Ellen.Kenchington@dfo-mpo.gc.ca](mailto:Ellen.Kenchington@dfo-mpo.gc.ca)

Research Vessel Multispecies Bottom Trawl Surveys (2006, 2008-2016), Fisheries And Oceans Canada - Central and Arctic Region, Contact: Ellen Kenchington, [Ellen.Kenchington@dfo-mpo.gc.ca](mailto:Ellen.Kenchington@dfo-mpo.gc.ca)

**Data provider:** Marine and Freshwater Research Institute (MFRI)

Marine and Freshwater Research Institute (MFRI) shared data on the occurrence of *Helicolenus dactylopterus* in Icelandic waters originate from the annual MFRI groundfish surveys carried out in spring and autumn. The spring survey takes place in March (1989 onwards) with around 500 bottom trawl tows obtained annually, mostly in shelf areas. The autumn survey (1996 onwards) takes place in October, with about 300 bottom trawls obtained annually, both on the shelf and in deeper waters (down to 1400 m).

***References***:

Marine Research Institute (2010) Manuals for the Icelandic bottom trawl surveys in spring and autumn. Hafrannsóknir 156.

**Data provider:** Temple University (TU)

Temple University shared presence data of two live scleractinian species – *Lophelia pertusa* and *Madrepora oculata –* on the mid-Atlantic continental margin of the United States. Presence points were generated from DEEP Sea Exploration to Advance Research on Corals/Canyons/Cold seeps (DEEP SEARCH) and NOAA ship *Okeanos Explorer* expeditions in 2018. Video imagery from two dives with the HOV *Alvin* aboard the R/V *Atlantis* (AL4962 & AL4963), and one dive off the *Okeanos Explorer* with the ROV *Deep Discoverer* (EX1806-7) were annotated and coral presence documented at a s^-1^ temporal resolution; all coral presence data were matched by timestamp to submersible position data.

***Acknowledgments***: TU acknowledges the captains and crews of the R/V *Atlantis* and the NOAA ship *Okeanos Explorer*. Funding for DEEP SEARCH comes from BOEM (contract #M17PC00009).

**Data provider**: Instituto do Mar, University of the Azores (IMAR-UAz)

IMAR-UAz, shared occurrence data of the six cold-water coral species in the Azores EEZ. These records were extracted from the Azores’ Marine Biological Reference Collection and database (COLETA). Records used in this study were originally collected by the CORAZON, HERMIONE and CoralFISH harbour sampling programs, CoralFISH and Discardless/MERCES/SPONGES fisheries observer program, ARDAÇO, CoralFISH, CONDOR, PESCPROF, DEECON and BIOMETORE research longline surveys, FISHOR experimental bottom trawl surveys, and multiple ROV video surveys such as those conducted within CoralFISH, CONDOR, BIOMETORE, MEDWAVES, and Blue Azores 2018. Occurrence records for *Helicolenus dactylopterus* from the ARDAÇO, OASIS, CoralFISH, CONDOR, PESCPROF, DEECON and BIOMETORE research longline surveys were also provided.

**Acknowledgments**: IMAR-UAz acknowledges all the fishermen that collaborated with the harbour sampling programs and fisheries observer program. Data used in this study was produced with funding from the HERMIONE project (FP7 GA 226354), OASIS (FP7 GA EVK3-CT-2002-00073) CORAZON project (Portuguese National Science Foundation FCT GA PTDC/MAR/72169/2006), DEECON project (FCT EURODEEP/0002/2007), PESCPROF (Interreg IIIB/MAC/4.2/M12), CoralFISH project (FP7 GA 213144), CONDOR project (EEA grants GA PT0040/2008), BIOMETORE project (EEA grants GA PT02), DiscardLess project (H2020 GA 633680), MERCES project (H2020 GA 689518), SPONGES project (H2020 GA 679849), MEDWAVES cruise through ATLAS project (H2020, GA 678760), and the Blue Azores expedition (National Geographic Pristine Seas program, Oceano Azul Foundation, and Waitt Institute). The ARQDAÇO annual demersal fish monitoring research cruises funded by the Regional Government of the Azores. We also thank all scientists and crew of the RV Arquipélago.

**References:**

Catarino, D., A. Rosa, E. Giacomello, A.F. Sousa, Í. Sampaio, G. Menezes (2009) Observatory for Long-term Study and Monitoring of Azorean Seamount Ecosystem: Relatório do Cruzeiro - CONDOR-31-V09. Universidade dos Açores, Departamento de Oceanografia e Pescas. Arquivos do DOP: Série Cruzeiros, Nº 8/2009, DOP/UAç AD-154, 25pp.

Catarino, D., A. Rosa, E. Giacomello, G. Graça, S. Bonanomi, S. Gomes, G. Menezes (2010) Relatório do cruzeiro - CONDOR-34-O10. Universidade dos Açores, Departamento de Oceanografia e Pescas. Arquivos do DOP: Série Cruzeiros, Nº 3/2010, DOP/UAç AD-159, 25pp.

Catarino, D., G. Menezes, A. Rosa, M.R. Pinho (2010) Relatório do cruzeiro de Monitorização de Demersais realizado durante a Primavera de 2010 nos Açores (ARQDAÇO-32-P10). Universidade dos Açores, Departamento de Oceanografia e Pescas. Arquivos do DOP: Série Cruzeiros, 1/2010, DOP/UAç AD-157, 22pp.

Catarino, D., G. Menezes, M.R. Pinho, A. Rosa, O. Melo (2007) Relatório do cruzeiro de Monitorização de Demersais realizado durante a Primavera de 2007 nos Açores (ARQDAÇO-27-P07). Universidade dos Açores, Departamento de Oceanografia e Pescas. Arquivos do DOP: Série Cruzeiros, 1/2007, DOP/UAç AD-87, 19pp.

Catarino, D., G. Menezes, M.R. Pinho, A. Rosa, O. Melo (2008) Relatório do cruzeiro de Monitorização de Demersais realizado durante a Primavera de 2008 nos Açores (ARQDAÇO-29-P08). Universidade dos Açores, Departamento de Oceanografia e Pescas. Arquivos do DOP: Série Cruzeiros, 1/2008, DOP/UAç AD-94, 23pp.

Catarino, D., S. Stefanni, H. Parra, C. Noever, V. Carmo, C. Louteiro, G. Menezes (2018). Relatório do Cruzeiro - DEECON-30-V08. Universidade dos Açores, Departamento de Oceanografia e Pescas, 2008. Arquivos do DOP: Série Cruzeiros, 2/2008, DOP/UAç AD-95, 23pp.

Catarino, D., T. Morato, A. Rosa, V. Matos, C. Pham, E. Giacomerllo, G. Graça, G. Menezes (2011). Relatório de Cruzeiro - CORALFISHD-37-011. Universidade dos Açores, Departamento de Oceanografia e Pescas. Arquivos do DOP: Série Cruzeiros, Nº3/2011, DOP/UAç AD-168.

Catarino, D., T. Morato, V. Matos, G. Menezes (2010). Relatório de cruzeiro - CORALFISHD-33-V10. Universidade dos Açores, Departamento de Oceanografia e Pescas. Arquivos do DOP: Série Cruzeiros, Nº 2/2010, DOP/UAç AD-158, 24pp.

dos Santos A., G. Menezes, M. Biscoito, E. Giacomello, A. Campos, A. Teixeira, J. Delgado, M. Carreiro-Silva, M. Tuaty Guerra, M. Silva, R. Caldeira, T. Morato, A. Cartaxana, A.D. Silva, A. Peliz, A.M. Martins, A. Moreno, C. Dâmaso, C. Bartilotti, I. Sousa-Pinto, I. Figueiredo, P. Afonso, T. Moura (2017). BIOMETORE -Biodiversity in seamounts: the Madeira-Tore and Great Meteor (PT02_Aviso2_001) -Final report - Scientific component (9 June 2015 - 30 April 2017).311 pp.

Marine and Freshwater Research Institute (2019) MFRI benthic trawl surey database, unpublished data

Melo, O., G. Menezes (2002). Projecto de Acompanhamento da Experiência de Pesca Dirigida ao Peixe-Relógio (*Hoplostethus atlanticus*) - FISHOR. Relatório final. Universidade dos Açores, Departamento de Oceanografia e Pescas. Arquivos do DOP, Série Estudos, 4/2002, DOP/UAç AD-29, 38 pp.

Melo, O., G.M. Menezes, M.R. Pinho, A. Rosa, E. Isidro (2005) Relatório do Cruzeiro de Monitorização de Demersais realizado durante a Primavera de 2004 nos Açores (ARQDAÇO-21-P04). Universidade dos Açores, Departamento de Oceanografia e Pescas. Arquivos do DOP: Série Relatórios Internos, 1/2005, DOP/UAç AD-134, 18pp.

Morato, T., M. Carreiro-Silva, G.H. Taranto, C. Dominguez-Carrió, M. Ramos, N. Ríos, L. Fauconnet, O.O. Vicente, A. Calado, A. Afonso, B. Ramos, M. Souto, R. Bettencourt, E. Gonçalves (2018). Blue Azores program: Expedition 2018 on board the NRP Gago Coutinho. 1st Leg, 3rd to 9th June 2018; 2nd leg, 14th to 24th June 2018. Cruise Report. 40pp.

Orejas, C., A. Addamo, M. Alvarez, A. Aparicio, D. Alcoverro, S. Arnaud-Haond, M. Bilan, J. Boavida, V. Cainzos, R. Calderon, P. Cambeiro, M. Castano, A. Fox, M. Gallardo, A. Gori, C. Guituerrez, H. Lea-Anne, M. Hermida, J.A. Jimenez, J.L. Lopez-Jurado, P. Lozano, A. Mateo-Ramirez, M. Guillem, J.L. Matoso, C. Mendez, A. Morillas, J. Movilla, A. Olariaga, M. Paredes, V. Pelayo, S. Pineiro, M. Rakka, T. Ramirez, M. Ramos, J. Reis, J. Rivera, A. Romero, J.L. Rueda, T. Salvador, Í. Sampaio, H. Sanchez, R. Santiago, A. Serrano, G. Taranto, J. Urra, P. Velez-Belchi, N. Viladrich, M. Zein (2017). Cruise Summary Report – MEDWAVES survey (MEDiterranean out flow WAter and Vulnerable EcosystemS). Zenodo. http://doi.org/10.5281/zenodo.556516

Rosa, A., D. Catarino, M. De Girolamo, S. Gomes, M.R. Pinho, G. Menezes, E. Giacomello (2017) Relatório do Cruzeiro de Monitorização de Demersais realizado durante a Primavera de 2016 nos Açores (ARQDAÇO-46-P16) (2017). Universidade dos Açores, Departamento de Oceanografia e Pescas. Arquivos do DOP: Série Cruzeiros Nº1/2017, DOP/UAç AD-194, 19pp.

Rosa, A., D. Catarino, S. Gomes, A. Pablon, C. Viegas, M. Pastor, H. Parra, C. Loureiro, M.R. Pinho, E. Giacomello (2018) Relatório do Cruzeiro de Monitorização de Demersais realizado durante a Primavera de 2017 nos Açores (ARQDAÇO-48-P17) (2017). Universidade dos Açores, Departamento de Oceanografia e Pescas. Arquivos do DOP: Série Cruzeiros Nº1/2018, DOP/UAç AD-196, 19pp.

Rosa, A., G.M. Menezes, M.R. Pinho, O. Melo, E. Isidro (2005) Relatório de Cruzeiro de Pesca de Profundidade realizado durante o Verão de 2004 nos Açores (PESCPROF-23-V04). Universidade dos Açores, Departamento de Oceanografia e Pescas, 2005. Arquivos do DOP: Série Cruzeiros, 2/2005, DOP/UAç AD-76, 17pp.

Santos, R.S., F. Porteiro, M. Carreiro-Silva, A. Braga-Henriques, E. Giacomello, A. Branco, P. Ferreira, T. Morato, P. Ribeiro, F. Tempera (2010) Cruise report: Condor, Voador and Banco Açores seamounts, Azores (5-14 August 2010). Projects: CoralFISH (IMAR, FP7), CONDOR (UAc, EEA), CORAZON (IMAR, FCT), Hermione (UAc, FP7). 12pp.

**Data provider:** L'Institut Français de Recherche pour l'Exploitation de la Mer (IFREMER)

Ifremer shared occurrence data on coral taxa that are indicators of VME in the Bay of Biscay. These records were obtained in 24 submarine canyons, from video annotations of 46 dives of the ROV *Victor 6000* and the towed camera *Scampi*, during six cruises carried out between 2009 and 2012 (BobGeo, BobGeo 2, and Evhoe 2009, 2010, 2011 and 2012). In addition, Ifremer shared occurrence data on four species of fish (*Helicolenus dactylopterus*, *Gadus morhu*a, *Hippoglossoides platessoides*, *Coryphaenoides rupestris*) from the Bay of Biscay. Those records were obtained using a 36/47 GOV bottom trawl during the multiannual International Bottom Trawl Survey EVHOE. The data provided were extracted from the ICES database DATRAS. The geographic coverage spans the latitude 44°N to 52°N. The bathymetric coverage spans the depth 20 to 800 m.

**Acknowledgments**: Ifremer wishes to thank the scientific party and crew members involved in the EVHOE cruises as well as the BobGeo and BobEco cruises. The data on VME indicator taxa were produced with funding from the CoralFISH project (FP7 GA 213144).

***References***:

Marine Research Institute (2010) Manuals for the Icelandic bottom trawl surveys in spring and autumn. Hafrannsóknir 156.

Arnaud-Haond S., Grehan A. (2011) BOBECO LEG1 cruise, RV Pourquoi pas?, <https://doi.org/10.17600/11030090>

Arnaud-Haond S., Grehan A. (2011) BOBECO LEG2 cruise, RV Pourquoi pas?, <https://doi.org/10.17600/11030180>

Bourillet J.-F. (2009) BOBGEO cruise, RV Pourquoi pas?, <https://doi.org/10.17600/9030060>

Bourillet J.-F. (2010) BOBGEO2 cruise, RV Le Suroît, https://doi.org/10.17600/10020020

ICES Database of Trawl Surveys (DATRAS),http://datras.ices.dk, 2019. ICES, Copenhagen

Mahe J.-C., Laffargue P. (1987) EVHOE EVALUATION HALIEUTIQUE OUEST DE L'EUROPE, <https://doi.org/10.18142/8>.

van den Beld, I.M.J., Bourillet, J.-F., Arnaud-Haond, S., de Chambure, L., Davies, J.S., Guillaumont, B., Olu, K., Menot, L., 2017. Cold-Water Coral Habitats in Submarine Canyons of the Bay of Biscay. Frontiers in Marine Science 4 (118), 10.3389/fmars.2017.00118.

van den Beld et al. (2018) The distribution of Cold Water Corals along the continental margins of the Bay of Biscay. SEANOE. https://doi.org/10.17882/53313.

**Data provider:** University of Edinburgh (UEDIN)

UEDIN data provided occurrence of the reef framework-forming coral *Lophelia pertusa* in the North Atlantic from Scottish waters, particularly the inshore Mingulay Reef Complex off Scotland. Data include ROV transects and Van Veen/videograb/boxcore stations.

***Acknowledgments:*** UEDIN acknowledges the UK Natural Environment Research Council (NERC) and UK Research and Innovation, Scottish Natural Heritage (SNH) and the Joint Nature Conservation Committee (JNCC).

***References***:

Achterburg E, 2011. Cruise Report D366/367 – June 6th – July 10th 2011, UK Ocean Acidification Research Program. RRS Discovery 366/367, 6th June to 12th July 2011.

Inall M. 2009. RRS Discovery Cruise D340b Dunstaffnage to Govan via Barra Head and the Surrounding Shelf, 26th June to 4th July 2009.

Last K, Roberts JM, Wicks L, Hogg M. 2010. Report of the RV Calanus cold-water coral research cruise to the Mingulay reef complex, 22-24th February 2010. SAMS and Heriot-Watt University.

Last K, Wicks L, de Franciso B, Douarin M. 2010. Report of the RV Calanus cold-water coral research cruise to the Mingulay reef complex, 3rd- 6th May 2010. SAMS, Heriot-Watt University, University of Edinburgh.

Roberts JM and shipboard party (2013) Changing Oceans Expedition 2012. RRS James Cook 073 Cruise Report. Heriot-Watt University. 224 pp.

Roberts JM, Brown CJ, Long D, Wilkinson CJ, Bates CR, A Mitchell, Service M. 2003. Final Report. Mapping INshore Coral Habitats the MINCH project

**Data provider:** Marine Scotland Science, Scotland, UK (MSS)

MSS shared occurrence fish data that arose from MSS's Rockall Haddock demersal trawl (1985-present) and Deep Water demersal trawl surveys (1999 – present). While MSS undertake other surveys, for example in the North Sea, these are routinely submitted to DATRAS as part of the IBTS. The data given represent raw numbers based on trawl duration and haven't been standardised on effort. Benthic invertebrate data is routinely submitted by MSS to the ICES VME database. Most, but not all, are bycatch from trawling.

***Acknowledgments***: MSS data was generated under work funded by the Scottish Government and contain information from the Scottish Government (Marine Scotland) licensed under the Open Government License v3.0. We would like to thank the master and crew of the MRV Scotia for help in sample collection.

***References***:

ICES Database of Trawl Surveys (DATRAS). International Bottom Trawl Survey (IBTS). http://datras.ices.dk. ICES, Copenhagen

ICES Vulnerable Marine Ecosystem (VME) Database. http://vme.ices.dk. ICES, Copenhagen

**Data provider:** InstitutoEspañol de Oceanografía (IEO) - Mallorca

IEO-Mallorca shared occurrence data collected during the MEDWAVES (MEDiterranean out flow WAter and Vulnerable EcosystemS) cruise. MEDWAVES was conducted on board the Spanish Research Vessel Sarmiento de Gamboa, took place from the 21^st^ September to the 26^th^ October 2016 and targeted Mediterranean and Atlantic geomorphological features under the potential influence of the Mediterranean Outflow Water (MOW). The main goals of the cruise were: (1) to characterize physically and bio-geochemically the MOW path and understand its interaction with the general Atlantic Meridional Overturning Circulation (AMOC) stream, from the Alborán Sea to the Azores, through the Gulf of Cádiz, and the Ormonde Seamount, exploring the relationship between the oceanographic settings of these target areas and the benthic ecosystems therein and (2) to characterize communities associated with the transition area, and sample for population genetic analysis aiming at understanding the way the populations located in the target areas contribute or have contributed to the connectivity between the Mediterranean Sea and the Atlantic Ocean. Data extracted from ROV video transects conducted with the Liropus ROV, during the MEDWAVES cruise for the following species: *Madrepora oculata*, *Lophelia pertusa*, *Desmophyllum dianthus*, *Solenosmilia variabilis*, *Acanthogorgia* cf. *armata*, *Acanella arbuscula*, *Funiculina quadrangularis*, *Pennatula* cf *aculeata*, *Helicolenus dactylopterus*, and *Coryphaenoides rupestris*.

***Acknowledgments***: IEO Mallorca thanks Ricardo Aguilar, Álvaro Altuna and Olga Reñones for help with taxonomical identification. The MEDWAVES cruise was supported by the ATLAS project and the Spanish Ministry of Economy, Industry and Competitivity. IEO Mallorca is also grateful to all the crew of the RV Sarmiento de Gamboa, the Marine Technology Unit (UTM – CSIC) and the Liropus ROV team from ACSM.

***References***:

Gori, A., Orejas, C., Madurell, T., Bramanti, L., Martins, M., Quintanilla, E., ... & Greenacre, M. (2013). Bathymetrical distribution and size structure of cold-water coral populations in the Cap de Creus and Lacaze-Duthiers canyons (northwestern Mediterranean). Biogeosciences (BG), 10(3), 2049-2060.

Orejas, C., A. Addamo, M. Alvarez, A. Aparicio, D. Alcoverro, S. Arnaud-Haond, M. Bilan, J. Boavida, V. Cainzos, R. Calderon, P. Cambeiro, M. Castano, A. Fox, M. Gallardo, A. Gori, C. Guituerrez, H. Lea-Anne, M. Hermida, J.A. Jimenez, J.L. Lopez-Jurado, P.Lozano, A. Mateo-Ramirez, M. Guillem, J.L. Matoso, C. Mendez, A. Morillas, J. Movilla, A. Olariaga, M. Paredes, V. Pelayo, S. Pineiro, M. Rakka, T. Ramirez, M. Ramos, J. Reis, J. Rivera, A. Romero, J.L. Rueda, T. Salvador, Í. Sampaio, H. Sanchez, R. Santiago, A. Serrano, G. Taranto, J. Urra, P. Velez-Belchi, N. Viladrich, M. Zein(2017). Cruise Summary Report – MEDWAVES survey (MEDiterranean out flow WAter and Vulnerable EcosystemS). Zenodo. <http://doi.org/10.5281/zenodo.556516>)

Orejas, C., Gori, A., Iacono, C. L., Puig, P., Gili, J. M., & Dale, M. R. (2009). Cold-water corals in the Cap de Creus canyon, northwestern Mediterranean: spatial distribution, density and anthropogenic impact. Marine Ecology Progress Series, 397, 37-51.

**Data provider:** InstitutoEspañol de Oceanografía (IEO) – Vigo

IEO–Vigo shared occurrence data for *Funiculina quadrangularis* and *Pennatula aculeata* collected during 2007-2017 bottom-trawl groundfish surveys carried out by the IEO jointly with the European Union (EU). Data was originated from three different surveys: i) the EU-Spain & Portugal Flemish Cap survey sampled all the Flemish Cap (NAFO Division 3M); ii) the Spanish 3L survey sampled the “Nose” of the Grand Banks of Newfoundland and the Flemish Pass (NAFO Division 3L) and iii) the EU-Spain 3NO sampling the Grand Banks of Newfoundland (NAFO Division 3NO).

***Acknowledgments***: IEO–Vigo are especially grateful to the scientific staff involved in the surveys and to the research vessel crew (RV “Vizconde de Eza”) for assistance at sea. EU NAFO groundfish surveys has been co-funded by the European Union through the European Maritime and Fisheries Fund (EMFF) within the National Program of collection, management and use of data in the fisheries sector and support for scientific advice regarding the Common Fisheries Policy.

**References**:

Reports of bottom trawl surveys (2007-2017) on board R/V Vizconde de Eza

Durán Muñoz, P. D., Sacau, M., García-Alegre, A., & Román, E. (2019). Cold-water corals and deep-sea sponges by-catch mitigation: Dealing with groundfish survey data in the management of the northwest Atlantic Ocean high seas fisheries. Marine Policy, 103712.

**Data provider:** Instituto Español de Oceanografía (IEO) – Málaga

IEO-Málaga shared occurrence data on *Madrepora oculata*, *Lophelia pertusa*, *Funiculina quadrangularis*, *Pennatula aculeata* and *Helicolenus dactylopterus* for the Gulf of Cádiz and the Alborán Sea. These data were obtained using samples and underwater images of different expeditions (INDEMARES-CHICA, ISUNEPCA, MEDWAVES). Samples of some of those species were obtained with a beam-trawl (2 m width, 60 cm height, 1cm mesh size) in the INDEMARES-CHICA expeditions in 2010 and 2011. In following years, underwater images were obtained with a Remotely Operated Vehicle (ROV) LIROPUS 2000 (INDEMARES-CHICA 0412, MEDWAVES) and one Underwater Camera Sled HORUS (UCSH) (ISUNEPCA expeditions from 2014 to 2017). Seafloor observations were generally captured between 0.5 and 2.5 m from the seafloor, for 1-3 hours (ROV), 0.6-1 hour (UCSA) and 10-15 minutes (UCSH) per transect. Underwater images were georeferenced with a transponder that allowed the determination of their position in relation to the research vessel along each transect.

***Acknowledgments***: IEO-Málaga thanks all colleagues, captains and crews who have participated in the data acquisition during the expeditions performed on board RV Emma Bardán, RV Cornide de Saavedra, RV Ramón Margalef, RV Ángeles Alvariño and RV Sarmiento de Gamboa between 2010 and 2017. This work was supported by INDEMARES/CHICA (LIFE 07/NAT/E/000732), ISUNEPCA (FBFEP/AC120123118_2014; IEO/2015-2017), INTEMARES (LIFE IP PAF) and ATLAS (grant agreement No 678760).

***References***:

Díaz-del-Río, V,. Bruque, G., Fernández-Salas, L.M., Rueda, J.L., González, E., López, N., Palomino, D., López, F.J., Farias, C., Sánchez, R., J.T. Vázquez JT, Rittierott, C., Fernández, A., Marina, P., Luque, V., Oporto, T., Sánchez, O., García, M., Urra, J., Bárcenas, P., Jiménez, M.P., Sagarminaga, R., Arcos, J.M., 2014. Volcanes de fango del golfo de Cádiz, Proyecto LIFE + INDEMARES. Ed. Fundación Biodiversidad del Ministerio de Agricultura, Alimentación y Medio Ambiente. 130 pp.

Orejas, C., A. Addamo, M. Alvarez, A. Aparicio, D. Alcoverro, S. Arnaud-Haond, M. Bilan, J. Boavida, V. Cainzos, R. Calderon, P. Cambeiro, M. Castano, A. Fox, M. Gallardo, A. Gori, C. Guituerrez, H. Lea-Anne, M. Hermida, J.A. Jimenez, J.L. Lopez-Jurado, P.Lozano, A. Mateo-Ramirez, M. Guillem, J.L. Matoso, C. Mendez, A. Morillas, J. Movilla, A. Olariaga, M. Paredes, V. Pelayo, S. Pineiro, M. Rakka, T. Ramirez, M. Ramos, J. Reis, J. Rivera, A. Romero, J.L. Rueda, T. Salvador, Í. Sampaio, H. Sanchez, R. Santiago, A. Serrano, G. Taranto, J. Urra, P. Velez-Belchi, N. Viladrich, M. Zein(2017). Cruise Summary Report – MEDWAVES survey (MEDiterranean out flow WAter and Vulnerable EcosystemS). Zenodo. <http://doi.org/10.5281/zenodo.556516>

Vila, Y. and ISUNEPCA Team (2015). Estimación de la abundancia de cigala *Nephrops norvegicus* en el golfo de Cádiz a través de imágenes submarinas. Memoria Técnica final. Proyecto financiado por la fundación Biodiversidad y FEP Acción gratuita cofinanciada por el Fondo Europeo de Pesca. 90 pp.

**Data provider:** University of Bari Aldo Moro (UOB)

University of Bari Aldo Moro (UOB) shared Mediterranean occurrence and distribution data of the colonial cold-water coral species *Madrepora oculata* and *Lophelia pertusa*, as well as the solitary coral *Desmophyllum dianthus* (Chimienti et al. 2018). Data was gathered by a multisource approach including research papers, conference proceedings, project reports and unpublished data (Chimienti et al., 2019 and references therein).

***Acknowledgments***: Part of my work has been funded by National Geographic (Grant EC-176R-18).

***References***:

Angeletti L, Taviani M, Canese S et al (2014) New deep-water cnidarian sites in the southern Adriatic Sea . Mediterr Mar Sci 15(2):225–238

Chimienti G, Bo M, Mastrototaro F (2018) Know the distribution to assess the changes: Mediterranean cold-water coral bioconstructions. Rend Lincei Sci Fis Nat 29:583–588. https://doi.org/10.1007/s12210-018-0718-3

Chimienti G, Bo M, Taviani M, Mastrototaro F (2019) Occurrence and biogeography of Mediterranean cold-water corals. In: Orejas C, Jiménez C (eds) Mediterranean Cold-Water Corals: Past, Present and Future. Coral Reefs of the World 9. AG Cham, Switzerland: Springer International Publishing 19:213–243. https://doi.org/10.1007/978-3-319-91608-8_19

Freiwald A, Boetius A, Bohrmann G (2011) Deep water ecosystems of the Eastern Mediterranean, Cruise No. 70, Leg 1 - 3. METEOR-Berichte, p 312

**Data provider:** National University of Ireland, Galway (NUIG)

NUIG shared occurrence (presence-only) data of living *Lophelia pertusa* reefs in the Irish continental margin, which were assembled from databases, cruise reports and publications. A total of 4423 records were inspected and quality assessed to ensure that they (1) represented confirmed living *L. pertusa* reefs (so excluding 2900 records of dead and isolated coral colony records); (2) were derived from sampling equipment that allows for accurate (<200 m) geo-referencing (so excluding 620 records derived mainly from trawling and dredging activities); and (3) were not duplicated. The remaining coral observations were highly clustered in regions targeted by research expeditions (which might lead to falsely inflated model evaluation measures), so the data were therefore coarsened by deleting all but one record within grid cells of 0.02 resolution. The final dataset comprised 53 records of living *Lophelia pertusa* reefs.

***Acknowledgments***: NUIG acknowledges the EU-FP7 CoralFISH project (grant no. 213144) and the H2020 ATLAS project (Grant agreement 678760).

***References***:

Rengstorf et al. (2013) Occurrence (presence-only) of living Lophelia pertusa reefs in the Irish continental margin. PANGAEA, <https://doi.org/10.1594/PANGAEA.809726>

Rengstorf, A.M., Yesson, C., Brown, C., Grehan, A.J., 2013. High-resolution habitat suitability modelling can improve conservation of vulnerable marine ecosystems in the deep sea. J. Biogeogr. 40, 1702–1714.

**Data provider:** MARUM, University of Bremen, Germany (UNI-HB)

MARUM UNI-HB data provided are based on four expeditions to various regions at the Irish margin (Ratmeyer et al., 2006; Wienberg et al., 2008), the Gulf of Cadiz (Hebbeln et al., 2008; Wienberg et al., 2013), the Alboran Sea (Hebbeln et al., 2009), and the southeastern Gulf of Mexico (Hebbeln et al., 2012; 2014). During all these expeditions, an ROV has been deployed to allow for detailed in-situ observations of cold-water coral settings at the sea floor.

***Acknowledgments***: UNI-HB greatly acknowledges the German Science Foundation (DFG) for providing ship time.

***References***:

Hebbeln et al. 2008, Report and preliminary results of RV PELAGIA cruise 64PE284: Cold-Water Corals in the Gulf of Cádiz and on Coral Patch Seamount (NE Atlantic), Portimão - Portimão, February 18 – March 09 2008. Berichte, Fachbereich Geowissenschaften, Universität Bremen. 265, 1-90 https://elib.suub.uni-bremen.de/ip/docs/00010373.pdf

Hebbeln et al. 2009, Report and preliminary results of RV POSEIDON Cruise POS385: Cold-Water Corals of the Alboran Sea (western Mediterranean Sea), Faro - Toulon, May 29 – June 16 2009. Berichte, Fachbereich Geowissenschaften, Universität Bremen. 273, 1-79 http://www.marum.de/Binaries/Binary45362/No_273_Hebbeln_et_al_2009.pdf

Hebbeln et al. 2012, West Atlantic Cold-Water Coral Ecosystems: The West Side Story (Report and preliminary results of RV Maria S. Merian Cruise MSM20-4), Bridgetown (Barbados) – Freetown (Bahamas), March 14 – April 7 2012. Berichte, Fachbereich Geowissenschaften, Universität Bremen. 273, 1-79, https://www.marum.de/Binaries/Binary2982/CruiseReport-MSM-20-4-final.pdf

Hebbeln et al. 2014, Environmental forcing of the Campeche cold-water coral province, southern Gulf of Mexico. Biogeosciences 11: 1799-1815 doi: 10.5194/bg-11-1799-2014.

Ratmeyer et al. 2006, Report and preliminary results of RV Meteor Cruise M61/3: development of carbonate mounds on the Celtic Continental Margin, Northeast Atlantic, Cork (Ireland) - Ponta Delgada (Portugal), 04. - 21.06.2004 Berichte, Fachbereich Geowissenschaften, Universität Bremen. 247, 1-64 , http://nbn-resolving.de/ urn:nbn:de:gbv:46-ep000106793

Wienberg et al. 2008, Franken Mound – facies and biocoenoses on a newly-discovered 'carbonate mound' on the western Rockall Bank, NE Atlantic. Facies, 54, 1-24.

Wienberg et al. 2013, Coral Patch seamount (NE Atlantic) – a sedimentological and megafaunal reconnaissance based on video and hydroacoustic surveys. Biogeosciences, 10, 3421-3443.

**Data provider:** University of North Carolina, Wilmington, USA (UNC-W)

UNC-W provided occurrence data for selected coral and fish species occurring in the area roughly from off Cape Lookout, NC to Baltimore Canyon (100-1600 m) from numerous research cruises conducted by S.W. Ross from 1991 through 2013. The most intense study sites were the Cape Lookout coral banks (Ross and Nizinski 2007), The Hatteras Middle Slope (Sulak and Ross 1996), and Norfolk and Baltimore canyons (CSA et al. 2017). These cruises collected species occurrence data using HOVs, ROVs (video plus collections) and various nets. Basic oceanographic data (e.g., CTD data) were also collected. The cruises generally targeted complex habitats, including coral bioherms, canyons, seeps, and rocky reefs, but surrounding soft bottoms were also surveyed. Benthic terrain of all study areas had been mapped with multibeam sonars.

***Acknowledgments***: Funds and ship support were provided to S.W. Ross (UNC-W) for the cruise data used in this study by the NC Legislature, NOAA Office of Exploration and Research, US Geological Survey, Bureau of Ocean Energy Management, NOAA National Undersea Research Center at UNCW, and the Duke-UNC Oceanographic Consortium.

***References***:

S.W. Ross Lab unpublished data as needed

Brooke, S., S.W. Ross, J.M. Bane, H.E. Seim and C.M. Young. 2013. Temperature tolerance of the deep-sea coral *Lophelia pertusa* from the southeastern United States. Deep-Sea Res. II 92: 240-248.

Brooke, S.D., M.W. Watts, A.D. Heil, M. Rhode, F. Mienis, G.C.A. Duineveld, A.J. Davies and S.W. Ross. 2017. Distributions and habitat associations of deep water corals in Norfolk and Baltimore canyons, Mid-Atlantic Bight, USA. Deep-Sea Res. II. 137: 131-147.

CSA Ocean Sciences Inc., S. Ross, S. Brooke, E. Baird, E. Coykendall, A. Davies, A. Demopoulos, S. France, C. Kellogg, R. Mather, F. Mienis, C. Morrison, N. Prouty, B. Roark and C. Robertson. 2017. Exploration and Research of Mid-Atlantic Deepwater hard Bottom Habitats and Shipwrecks with Emphasis on Canyons and Coral Communities: Atlantic Deepwater Canyons Study. Vol I. Final Technical Rept., Vol. II: Final Appendices. U.S. Dept. of the Interior, Bureau of Ocean Energy Management, Atlantic OCS Region. OCS Study BOEM 2017-060 (Vol. I) & 061 (Vol. II). 1000 p + appendices.

CSA Ocean Sciences Inc., F.C. De Leo and S.W. Ross. 2019. Large submarine canyons of the United States outer continental shelf atlas. Sterling (VA): US Dept. of the Interior, Bureau of Ocean Energy Management. OCS Study BOEM 2019-066. 51 p.

Hourigan, T.F., J. Reed, S. Pomponi, S.W. Ross, A.W. David, S. Harter. 2017. State of Deep‐Sea Coral and Sponge Ecosystems of the Southeast United States. In: Hourigan, T.F., P.J. Etnoyer, S.D. Cairns (eds.). The State of Deep‐Sea Coral and Sponge Ecosystems of the United States. NOAA Technical Memo. NMFS‐OHC‐4, Silver Spring, MD. 60 p.

Mienis, F., G. Duineveld, A.J. Davies, M. Lavaleye, S.W. Ross, H. Seim, J. Bane, H. van Haren, M. Bergman, H. de Haas, S. Brooke, T. van Weering. 2014. Cold-water coral growth under extreme environmental conditions, the Cape Lookout area, NW Atlantic. Biogeosciences 11: 2543-2560.

Obelcz, J., D. Brothers, J. Chaytor, U. ten Brink, S.W. Ross and S. Brooke. 2014. Geomorphic characterization of four shelf-sourced submarine canyons along the U.S Mid-Atlantic continental margin. Deep-Sea Res. II 104: 106-119.

Partyka, M.L., S.W. Ross, A.M. Quattrini, G.R. Sedberry, T.W. Birdsong and J. Potter. 2007. Southeastern United States deep sea corals (SEADESC) initiative: a collaborative effort to characterize areas of habitat-forming deep-sea corals. NOAA Tech. Memo. OAR OER 1. Silver Spring, MD. 176 pp.

Ross, S.W. and A.M. Quattrini. 2007. The Fish Fauna Associated with Deep Coral Banks off the Southeastern United States. Deep-Sea Res. Part I 54(6): 975-1007.

Ross, S.W. and M.S. Nizinski. 2007. State of Deep Coral Ecosystems in the U.S. Southeast Region: Cape Hatteras to southeastern Florida. p. 233-270, Ch 6 In: Lumsden, S.E., T.F. Hourigan, A.W. Bruckner and G. Dorr (eds.). The State of Deep Coral Ecosystems of the United States. NOAA Tech. Memo. CRCP-3. Silver Spring, MD. 365 pp.

Ross, S.W., M.C.T. Carlson and A.M. Quattrini. 2012. The utility of museum records for documenting distributions of deep-sea corals off the southeastern United States. Mar. Biol. Res. 8(2): 101-114.

Ross, S.W., M. Rhode, A.M. Quattrini. 2015. Demersal fish distribution and habitat use within and near Baltimore and Norfolk Canyons, U.S. middle Atlantic slope. Deep-Sea Res. I 103: 137-154.

Ross, S.W., M. Rhode, S.T. Viada and R. Mather. 2016. Fish species associated with shipwreck and natural hard- bottom habitats from the middle to outer continental shelf of the Middle Atlantic Bight near Norfolk Canyon. Fish. Bull. 114:45-57.

Sulak, K.J. and S.W. Ross. 1996. Lilliputian bottom fish fauna of the Hatteras upper middle continental slope. J. Fish. Biol. 49 (Suppl. A): 91-113.

**Data provider:** P.P. Shirshov Institute of Oceanology RAS

P.P. Shirshov Institute of Oceanology RAS (IORAS) provided data on cold-water corals from the Mid-Atlantic Ridge and Reykjanes Ridge, and adjacent area based on long-term deep-sea biological collection of IORAS including trawls and HOVs samples (some of samples were also reported by Keller, 1984, 1985; Pasternak, 1985) and also collaborative MARECO cruise (reported by Molodtsova et al., 2008).

***Acknowledgments:*** IORAS data collection was funded by RF state Assignment 0149-2019-0009.

***References:***

Keller, N.B. 1984. The agermatypic corals of the Great Meteor seamount and their peculiarities. Trudy Instituta Okeanologii RAN SSSR,Moskva 119:60-64. Russian with English summary.

Keller NB. 1985. The madreporarian corals of the Reykjanes Ridge and the Platon submarine mountains (the north part of Atlantic Ocean). Trudy Instituta Okeanologii RAN SSSR Moskva 120:39-51. Russian with English summary.

Molodtsova, T. N., Sanamyan, N. P., & Keller, N. B. (2008). Anthozoa from the northern Mid-Atlantic Ridge and Charlie-Gibbs fracture zone. Marine Biology Research, 4(1-2), 112-130.

Pasternak FA. 1985. Speciﬁc composition and the ways of forming of the bottom fauna isolated underwater rises. Gorgonians and antipatharians of the seamounts Rockaway, Atlantis, Plato, Great Meteor and Josephine (Atlantic Ocean). Trudy Instituta Oceanologii AN SSSR, Moskva 120:2138. Russian with English summary.

**Supplementary Tables**

**Supplementary Table S2. Model performance statistics for the different modelling approaches.** The ability of the training data to predict the probability of presence was tested with different statistical metrics using the maximum sensitivity and specificity (MSS) threshold. The weighting factor (W_f_) used to produce the ensemble model outputs are also shown.

| **Species** | **Modelling**  **approach** | **Relative importance** | | |
| --- | --- | --- | --- | --- |
|  |  | **AUC** | **TSS** | **W_f_** |
| *Lophelia pertusa* | GAM | 0.86±0.10 | 0.64±0.18 | 0.32 |
|  | Maxent | 0.89±0.06 | 0.69±0.13 | 0.34 |
|  | RF | 0.90±0.07 | 0.66±0.14 | 0.33 |
| *Madrepora oculata* | GAM | 0.88±0.07 | 0.70±0.15 | 0.33 |
|  | Maxent | 0.90±0.06 | 0.71±0.13 | 0.34 |
|  | RF | 0.90±0.06 | 0.70±0.13 | 0.33 |
| *Desmophyllum dianthus* | GAM | 0.90±0.06 | 0.70±0.13 | 0.32 |
|  | Maxent | 0.93±0.04 | 0.76±0.09 | 0.34 |
|  | RF | 0.94±0.02 | 0.79±0.05 | 0.32 |
| *Acanthogorgia armata* | GAM | 0.92±0.06 | 0.77±0.10 | 0.34 |
|  | Maxent | 0.93±0.05 | 0.78±0.12 | 0.34 |
|  | RF | 0.91±0.06 | 0.73±0.15 | 0.34 |
| *Acanella arbuscula* | GAM | 0.86±0.05 | 0.63±0.11 | 0.32 |
|  | Maxent | 0.88±0.04 | 0.66±0.13 | 0.33 |
|  | RF | 0.90±0.05 | 0.69±0.12 | 0.33 |
| *Paragorgia arborea* | GAM | 0.89±0.10 | 0.68±0.22 | 0.32 |
|  | Maxent | 0.93±0.08 | 0.75±0.17 | 0.34 |
|  | RF | 0.90±0.12 | 0.73±0.19 | 0.33 |
| *Helicolenus dactylopterus* | GAM | 0.97±0.02 | 0.85±0.05 | 0.33 |
|  | Maxent | 0.98±0.01 | 0.87±0.04 | 0.34 |
|  | RF | 0.97±0.02 | 0.82±0.04 | 0.33 |
| *Sebastes mentella* | GAM | 0.98±0.01 | 0.85±0.03 | 0.34 |
|  | Maxent | 0.97±0.01 | 0.84±0.01 | 0.33 |
|  | RF | 0.97±0.01 | 0.84±0.01 | 0.33 |
| *Gadus morhua* | GAM | 0.95±0.02 | 0.81±0.03 | 0.34 |
|  | Maxent | 0.94±0.02 | 0.79±0.01 | 0.33 |
|  | RF | 0.95±0.03 | 0.78±0.14 | 0.34 |
| *Hippoglossoides platessoides* | GAM | 0.94±0.02 | 0.79±0.06 | 0.34 |
|  | Maxent | 0.93±0.01 | 0.80±0.03 | 0.34 |
|  | RF | 0.94±0.04 | 0.71±0.16 | 0.34 |
| *Reinhardtius hippoglossoides* | GAM | 0.87±0.01 | 0.61±0.02 | 0.33 |
|  | Maxent | 0.87±0.01 | 0.62±0.02 | 0.33 |
|  | RF | 0.87±0.01 | 0.62±0.02 | 0.33 |
| *Coryphaenoides rupestris* | GAM | 0.99±0.01 | 0.92±0.02 | 0.33 |
|  | Maxent | 0.99±0.00 | 0.92±0.02 | 0.33 |
|  | RF | 0.99±0.01 | 0.91±0.04 | 0.33 |

**Supplementary Table S3. Importance of each variable to the individual habitat suitability models predictions**, measured as 1-Pearson correlation, for six species of Vulnerable Marine Ecosystem indicators and six deep-sea fish in the North Atlantic Ocean. Predictor variable were depth, slope, Bathymetric Position Index (BPI), temperature at seafloor (Temp), particulate organic carbon flux to seafloor (POC), and Aragonite (Ωar) and Calcite (Ωcal) saturation state at seafloor. Modelling approaches used were: Generalized Additive Model (GAM), maximum entropy model (Maxent), and Random Forest (RF).

| **Species** | **Modelling**  **approach** | **Relative importance** | | | | | | |
| --- | --- | --- | --- | --- | --- | --- | --- | --- |
|  |  | ***Depth*** | ***Slope*** | ***BPI*** | ***Temp*** | ***POC*** | ***Ωar*** | ***Ωcal*** |
| *Lophelia pertusa* | GAM | - | 0.02±0.00 | 0.01±0.00 | 0.34±0.01 | 0.13±0.00 | 0.55±0.01 | - |
|  | Maxent | - | 0.06±0.00 | 0.02±0.00 | 0.59±0.01 | 0.35±0.01 | 0.17±0.00 | - |
|  | RF | - | 0.11±0.00 | 0.13±0.00 | 0.31±0.01 | 0.29±0.01 | 0.35±0.00 | - |
| *Madrepora oculata* | GAM | - | 0.04±0.00 | 0.01±0.00 | 0.44±0.01 | 0.49±0.01 | 0.09±0.00 | - |
|  | Maxent | - | 0.21±0.01 | 0.06±0.00 | 0.65±0.02 | 0.37±0.01 | 0.08±0.00 | - |
|  | RF | - | 0.22±0.01 | 0.13±0.01 | 0.31±0.01 | 0.35±0.01 | 0.24±0.01 | - |
| *Desmophyllum dianthus* | GAM | - | 0.09±0.00 | 0.01±0.00 | 0.26±0.00 | 0.76±0.01 | 0.07±0.00 | - |
|  | Maxent | - | 0.38±0.01 | 0.04±0.00 | 0.62±0.02 | 0.38±0.01 | 0.06±0.00 | - |
|  | RF | - | 0.35±0.01 | 0.09±0.00 | 0.44±0.02 | 0.30±0.01 | 0.20±0.01 | - |
| *Acanthogorgia armata* | GAM | - | 0.03±0.00 | 0.01±0.00 | 0.07±0.00 | 0.05±0.01 | - | 0.42±0.01 |
|  | Maxent | - | 0.30±0.01 | 0.02±0.00 | 0.19±0.01 | 0.54±0.02 | - | 0.47±0.02 |
|  | RF | - | 0.24±0.01 | 0.12±0.00 | 0.18±0.01 | 0.29±0.01 | - | 0.17±0.01 |
| *Acanella arbuscula* | GAM | - | 0.01±0.00 | 0±0 | 0.06±0.00 | 0.03±0.00 | - | 0.55±0.01 |
|  | Maxent | - | 0.11±0.00 | 0.01±0.00 | 0.21±0.01 | 0.38±0.01 | - | 0.44±0.01 |
|  | RF | - | 0.15±0.00 | 0.08±0.00 | 0.25±0.01 | 0.22±0.01 | - | 0.24±0.01 |
| *Paragorgia arborea* | GAM | - | 0±0 | 0.01±0.00 | 0.03±0.00 | 0.01±0.00 | - | 0.74±0.01 |
|  | Maxent | - | 0.05±0.00 | 0.08±0.00 | 0.19±0.01 | 0.65±0.01 | - | 0.72±0.02 |
|  | RF | - | 0.12±0.00 | 0.14±0.01 | 0.24±0.01 | 0.39±0.01 | - | 0.29±0.01 |
| *Helicolenus dactylopterus* | GAM | 0.95±0.01 | 0.47±0.00 | 0.48±0.00 | 0.48±0.00 | 0.47±0.00 | - | - |
|  | Maxent | 0.65±0.01 | 0.01±0.00 | 0.01±0.00 | 0.42±0.00 | 0.11±0.00 | - | - |
|  | RF | 0.32±0.00 | 0.02±0.00 | 0.07±0.00 | 0.46±0.01 | 0.12±0.00 | - | - |
| *Sebastes mentella* | GAM | 0.93±0.00 | 0.48±0.00 | 0.49±0.00 | 0.54±0.00 | 0.52±0.00 | - | - |
|  | Maxent | 0.62±0.01 | 0±0 | 0±0 | 0.32±0.00 | 0.57±0.00 | - | - |
|  | RF | 0.41±0.00 | 0.01±0.00 | 0.04±0.00 | 0.29±0.00 | 0.19±0.00 | - | - |
| *Gadus morhua* | GAM | 0.89±0.00 | 0.37±0.00 | 0.37±0.00 | 0.45±0.00 | 0.40±0.00 | - | - |
|  | Maxent | 0.71±0.00 | 0±0 | 0±0 | 0.18±0.00 | 0.51±0.00 | - | - |
|  | RF | 0.52±0.00 | 0.07±0.00 | 0.06±0.00 | 0.17±0.00 | 0.30±0.00 | - | - |
| *Hippoglossoides platessoides* | GAM | 0.75±0.00 | 0.31±0.00 | 0.29±0.00 | 0.46±0.00 | 0.33±0.00 | - | - |
|  | Maxent | 0.67±0.00 | 0±0 | 0±0 | 0.21±0.00 | 0.57±0.00 | - | - |
|  | RF | 0.50±0.00 | 0.05±0.00 | 0.05±0.00 | 0.20±0.00 | 0.33±0.00 | - | - |
| *Reinhardtius hippoglossoides* | GAM | 0.97±0.00 | 0.64±0.00 | 0.64±0.00 | 0.67±0.00 | 0.66±0.00 | - | - |
|  | Maxent | 0.59±0.00 | 0.01±0.00 | 0±0 | 0.39±0.00 | 0.55±0.00 | - | - |
|  | RF | 0.35±0.00 | 0.01±0.00 | 0.04±0.00 | 0.38±0.00 | 0.29±0.00 | - | - |
| *Coryphaenoides rupestris* | GAM | 0.88±0.01 | 0.48±0.00 | 0.47±0.00 | 0.48±0.00 | 0.49±0.00 | - | - |
|  | Maxent | 0.61±0.01 | 0.07±0.00 | 0±0 | 0.04±0.00 | 0.49±0.01 | - | - |
|  | RF | 0.51±0.00 | 0.08±0.00 | 0.02±0.00 | 0.09±0.00 | 0.27±0.00 | - | - |

**Supplementary Figures**


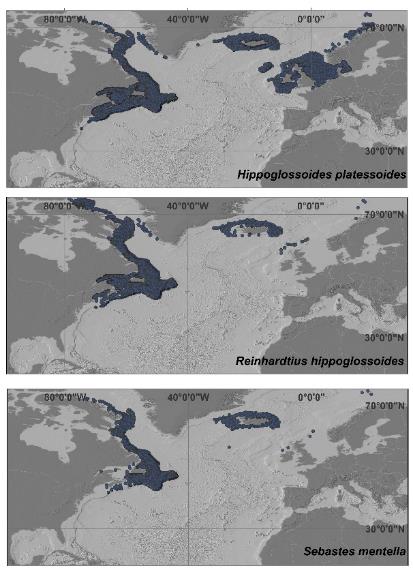


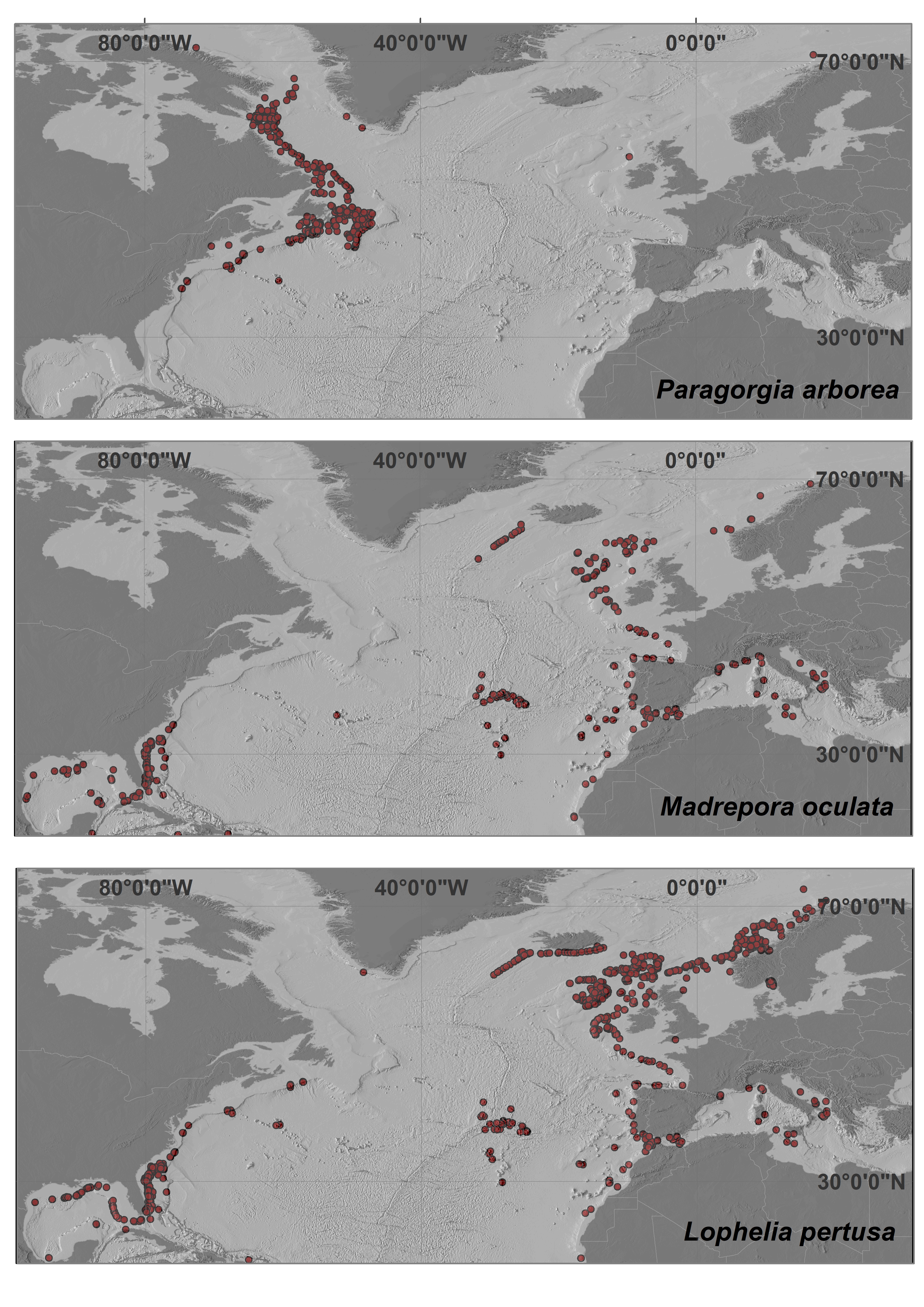

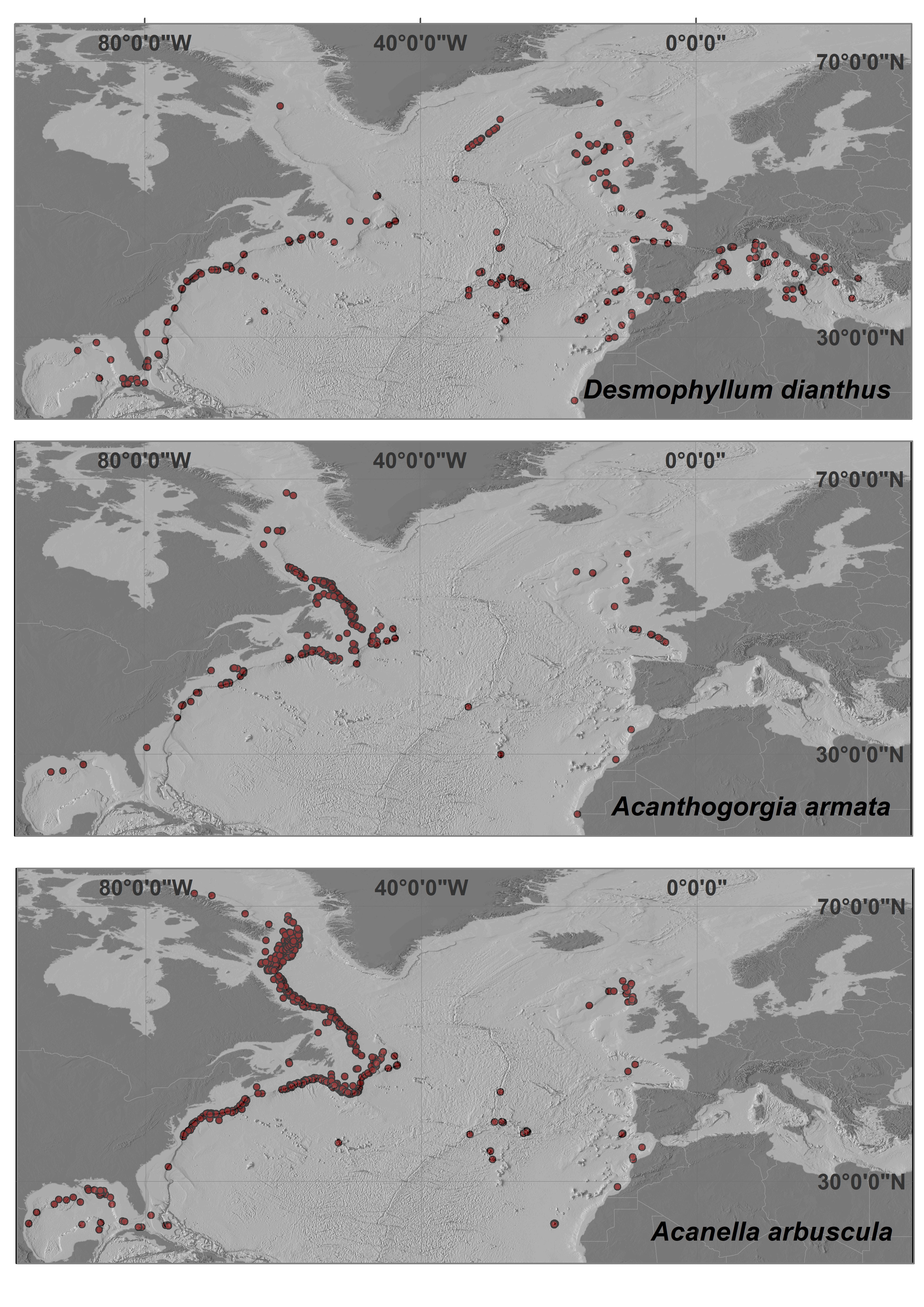

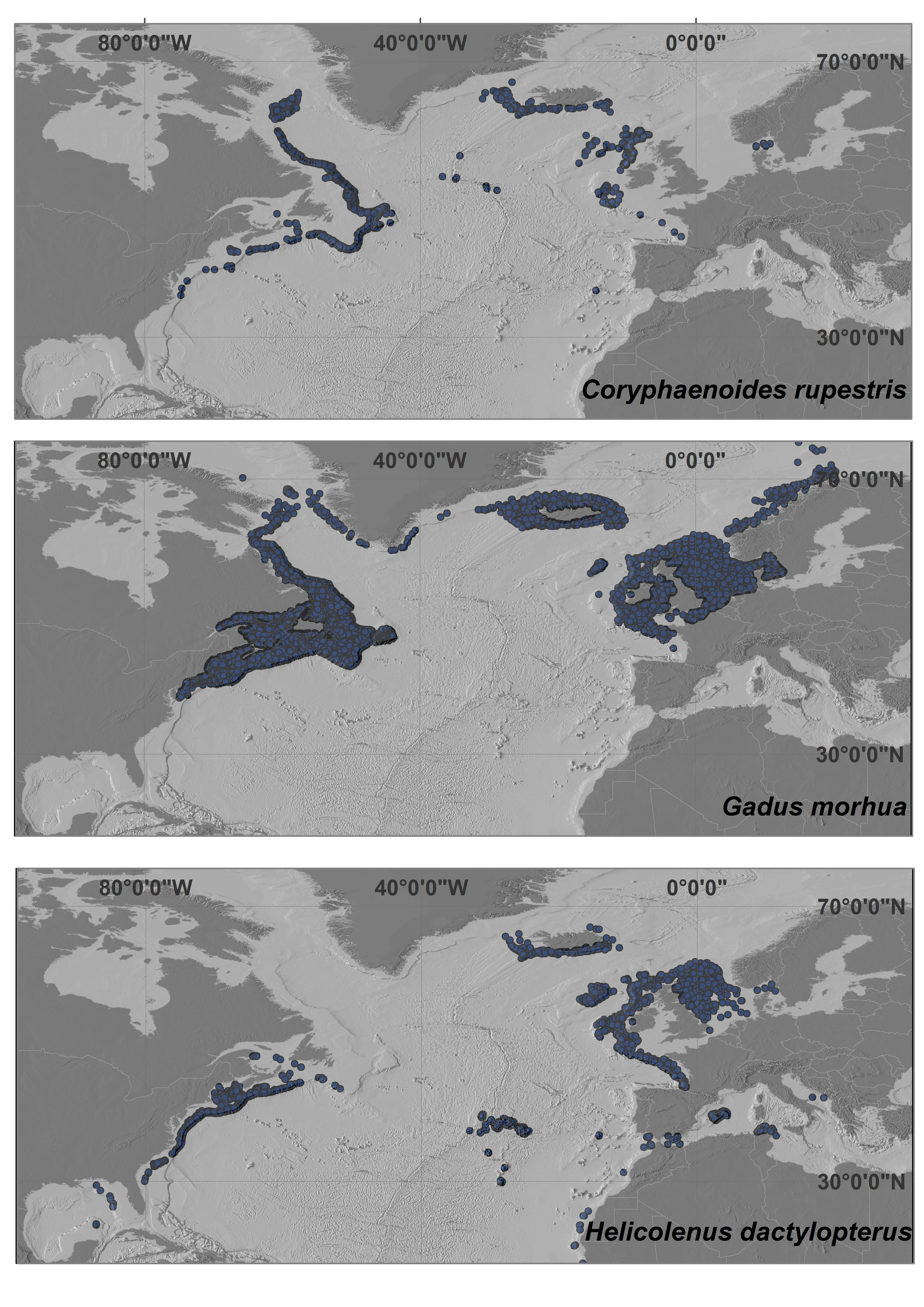


**Supplementary Figure S1.** Georeferenced presence-only records obtained for the twelve species of cold-water corals and commercially important deep-sea fish in the North Atlantic Ocean from institutional and public databases.

**Supplementary Figure S2.** Evaluation of the historical modelled (1951-2000) at seabed environmental layers’ against field observations. The correlation between the modelled and observed climatological mean were evaluated with the root-mean-square error (RMSE) and the Adjusted R-squared. The simulated values of POC-flux were compared against Lutz et al. (2007) estimates based sediment trap, and remotely sensed estimates of net primary production and sea surface temperature. Dissolved oxygen, pH and temperature were compared against the World Ocean Atlas 2013 (WOA13; Garcia et al., 2013), GLODAP (Olsen et al., 2016; 2019), and WOA13 (Locarnini et al., 2013), respectively. Errors in accuracy and precision have been described to be insufficient to offset the projected changes (e.g. Mora et al., 2013). Blue lines show 1:1 relationship.

**References:**

Garcia, H. E., Boyer, T. P., Locarnini, R. A., Antonov, J. I., Mishonov, A. V., Baranova, O. K., ... & Levitus, S. (2013). World ocean atlas 2013. Volume 3, Dissolved oxygen, apparent oxygen utilization, and oxygen saturation. In: S. Levitus & A. Mishonov (Eds.), NOAA Atlas NESDIS 75 (25 pp). Silver Spring: NOAA.

Locarnini, R. A., Mishonov, A. V., Antonov, J. I., Boyer, T. P., Garcia, H. E., Baranova, O. K., ... & Hamilton, M. (2013). World ocean atlas 2013. Volume 1, Temperature. In: S. Levitus & A. Mishonov (Eds.), NOAA Atlas NESDIS 73 (40 pp). Silver Spring: NOAA.

Lutz, M. J., Caldeira, K., Dunbar, R. B., & Behrenfeld, M. J. (2007). Seasonal rhythms of net primary production and particulate organic carbon flux to depth describe the efficiency of biological pump in the global ocean. Journal of Geophysical Research: Oceans, 112(C10011).

Olsen, A., Key, R. M., van Heuven, S., Lauvset, S. K., Velo, A., Lin, X., ... & Suzuki, T. (2016). The Global Ocean Data Analysis Project version 2 (GLODAPv2) – an internally consistent data product for the world ocean, Earth System Science Data 8, 297–323.

Olsen, A., Lange, N., Key, R. M., Tanhua, T., Álvarez, M., Becker, S., ... & Wanninkhof, R. (2019). GLODAPv2.2019 – an update of GLODAPv2. Earth System Science Data, 11, 1437-1461.

**
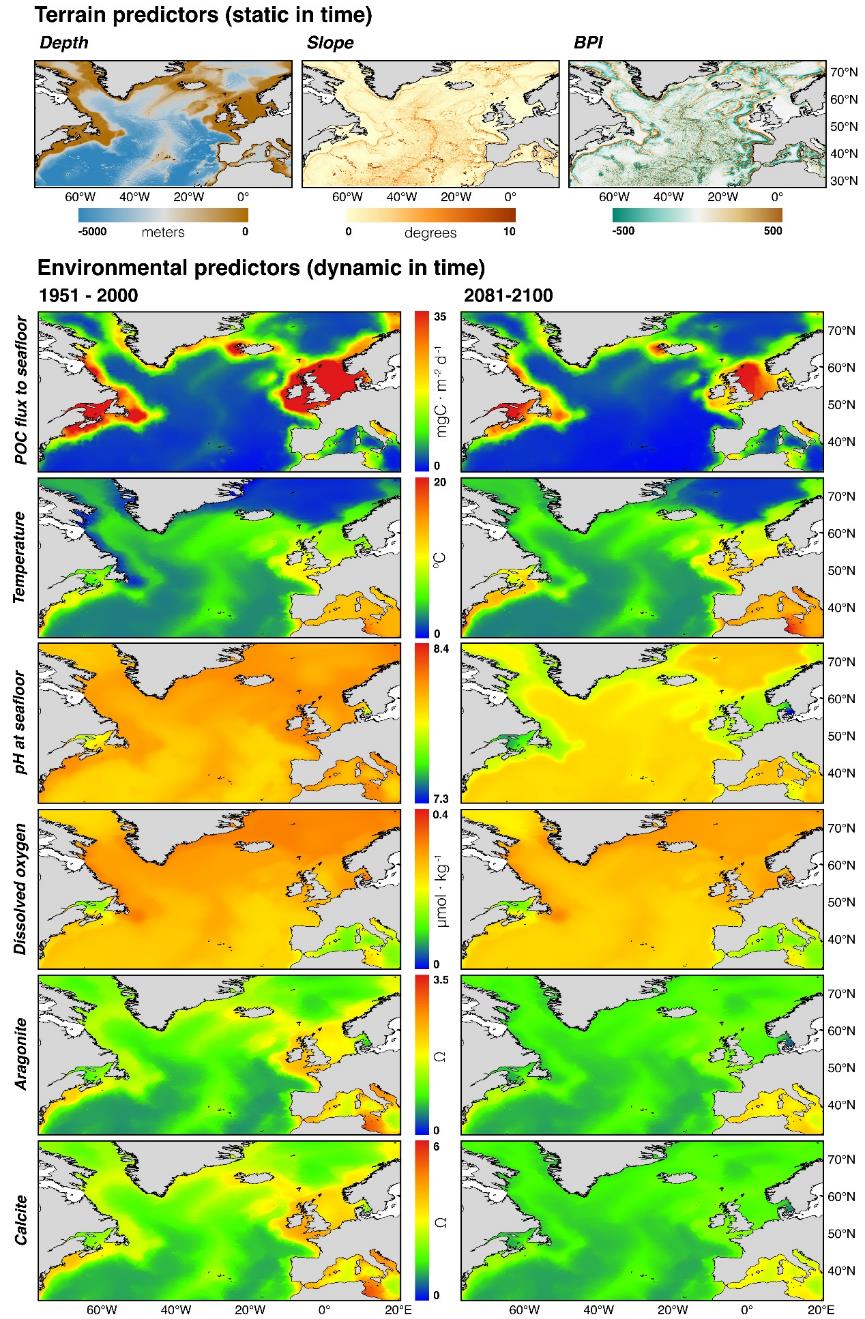
**

**Supplementary Figure S3.** Environmental predictors used to develop environmental niche models for present-day conditions and forecast habitat suitability under future environmental conditions in the North Atlantic Ocean.

**
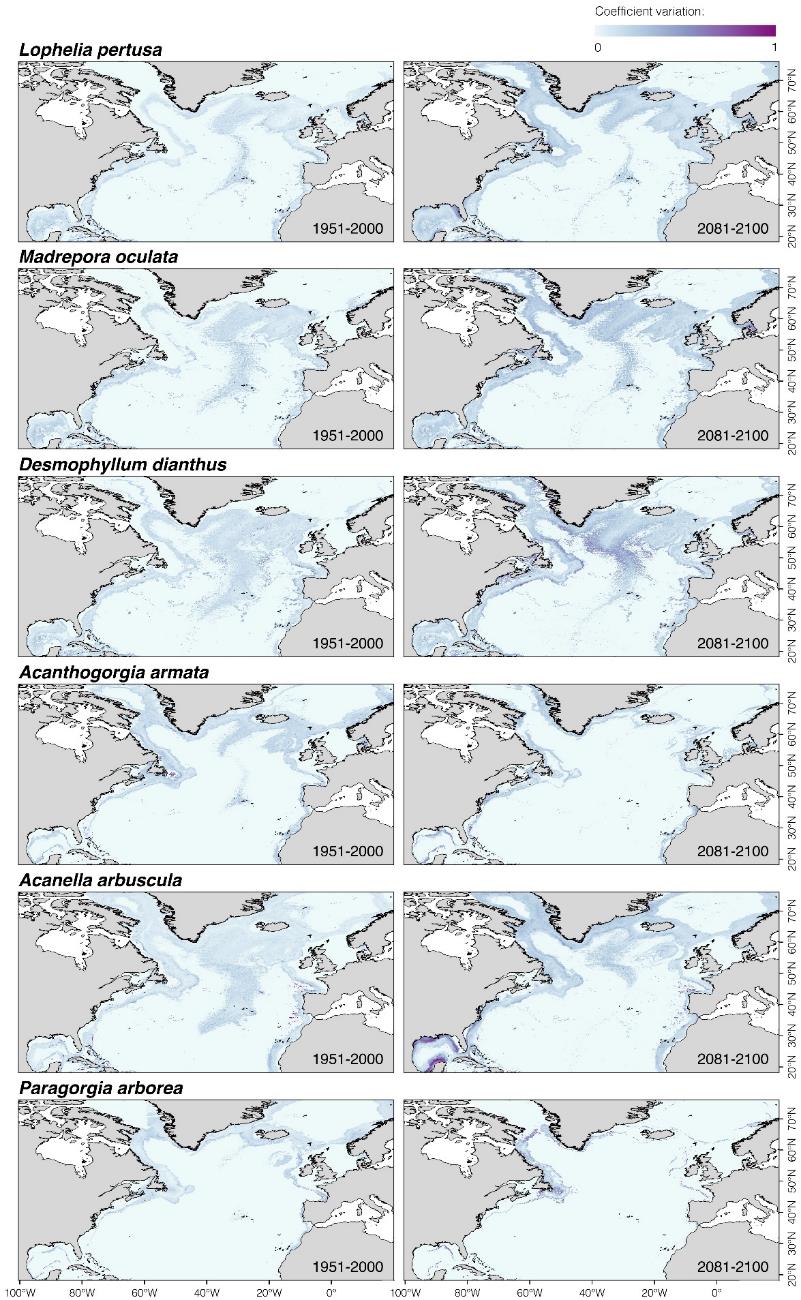
**

**Supplementary Figure S4a.** Uncertainty associated to the habitat suitability model predictions under present-day (1951-2000) and future (2081-2100; RCP8.5 or business-as-usual scenario) environmental conditions for cold-water corals in the North Atlantic Ocean using an ensemble modelling approach.

**
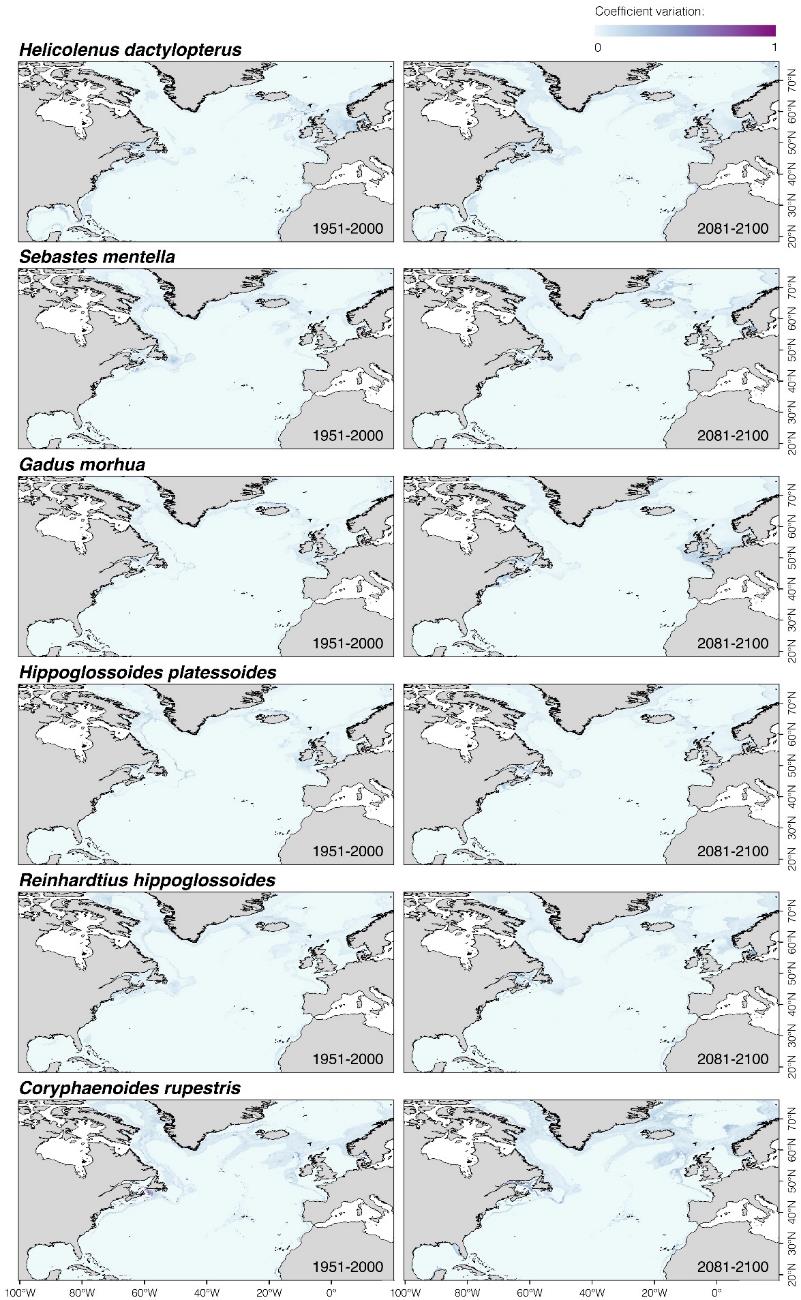
**

**Supplementary Figure S4b.** Uncertainty associated to the habitat suitability model predictions under present-day (1951-2000) and future (2081-2100; RCP8.5 or business-as-usual scenario) environmental conditions for cold-water corals in the North Atlantic Ocean using an ensemble modelling approach.

**
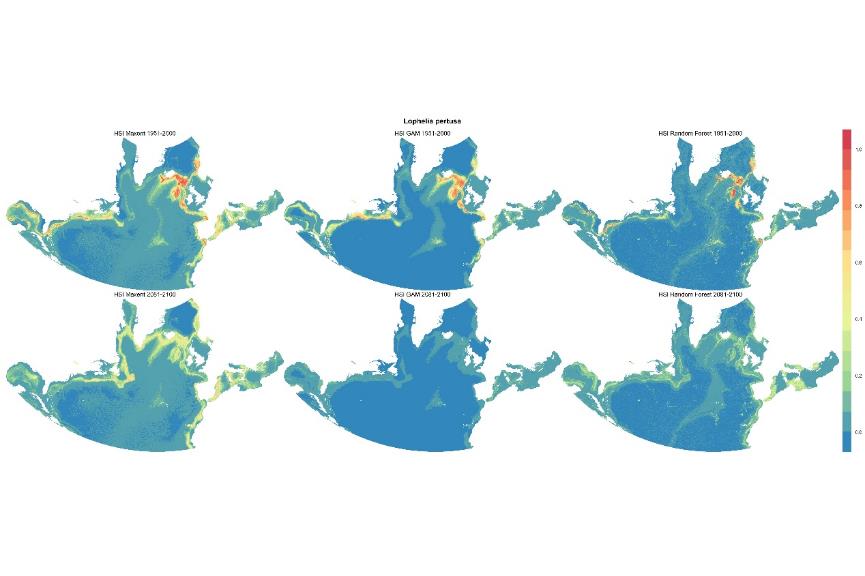
**

**
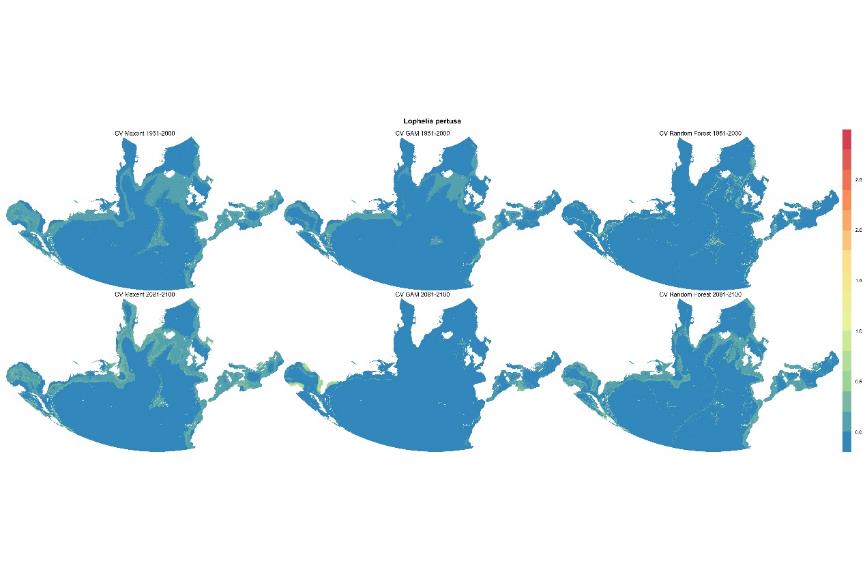
**

**Supplementary Figure S5a.** Predicted habitat suitability index (top) and Coefficient of Variance (CV; bottom) under present-day (1951-2000) and future (2081-2100; RCP8.5 or business-as-usual scenario) environmental conditions for the scleractinian coral *Lophelia pertusa* in the North Atlantic Ocean using three different modelling approaches: Generalized Additive Model (GAM), maximum entropy model (Maxent), and Random Forest (RF).

**
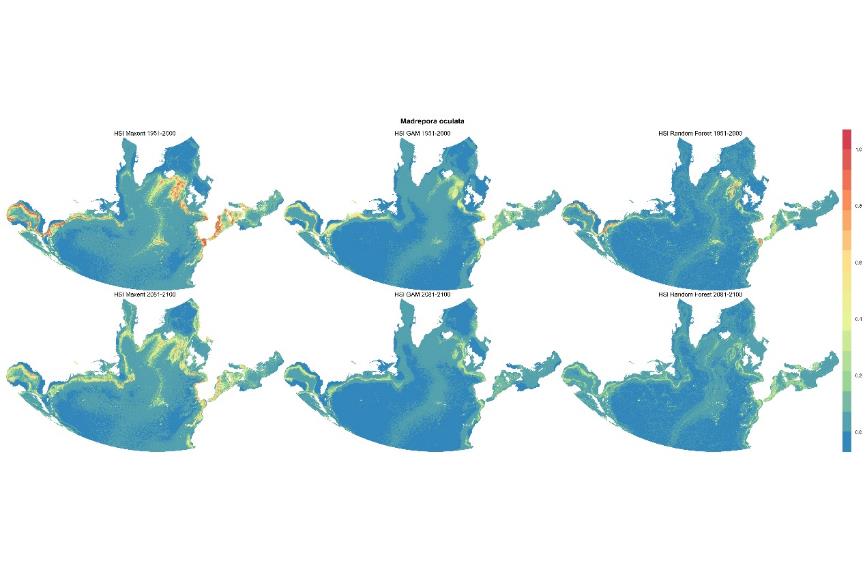

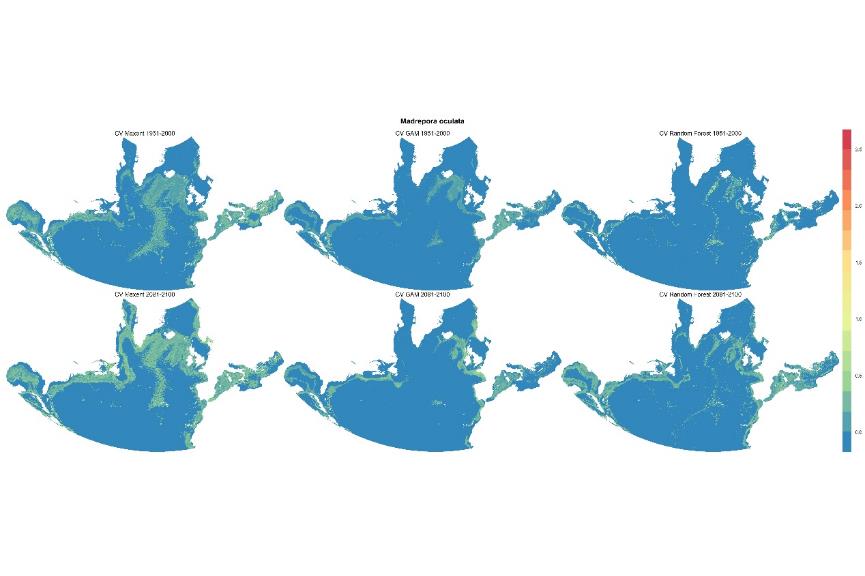
**

**Supplementary Figure S5b.** Predicted habitat suitability index (top) and Coefficient of Variance (CV; bottom) under present-day (1951-2000) and future (2081-2100; RCP8.5 or business-as-usual scenario) environmental conditions for the scleractinian coral *Madrepora oculata* in the North Atlantic Ocean using three different modelling approaches: Generalized Additive Model (GAM), maximum entropy model (Maxent), and Random Forest (RF).

**
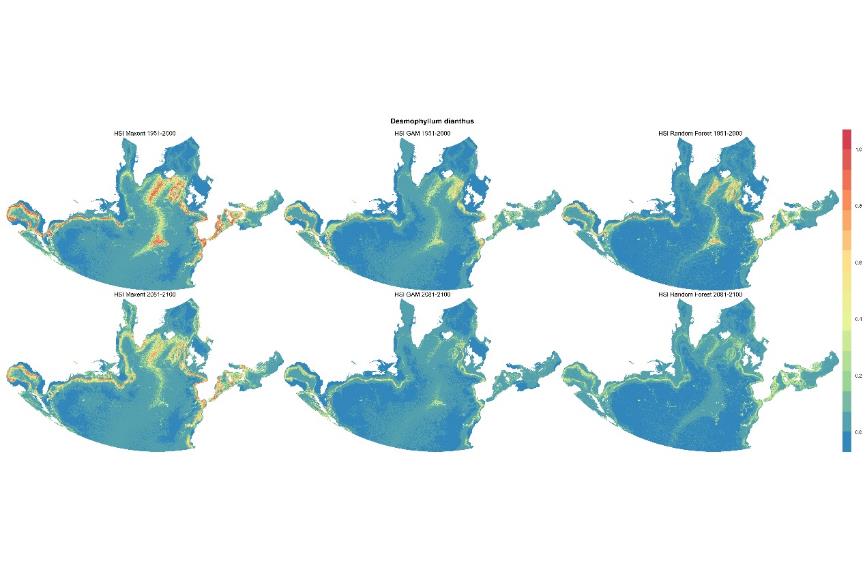

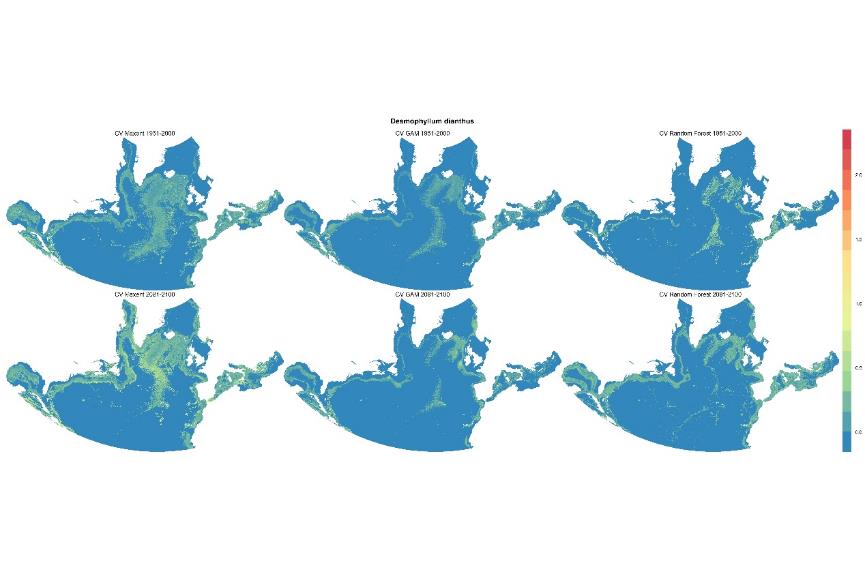
**

**Supplementary Figure S5c.** Predicted habitat suitability index (top) and Coefficient of Variance (CV; bottom) under present-day (1951-2000) and future (2081-2100; RCP8.5 or business-as-usual scenario) environmental conditions for scleractinian coral *Desmophyllum dianthus* in the North Atlantic Ocean using three different modelling approaches: Generalized Additive Model (GAM), maximum entropy model (Maxent), and Random Forest (RF).

**
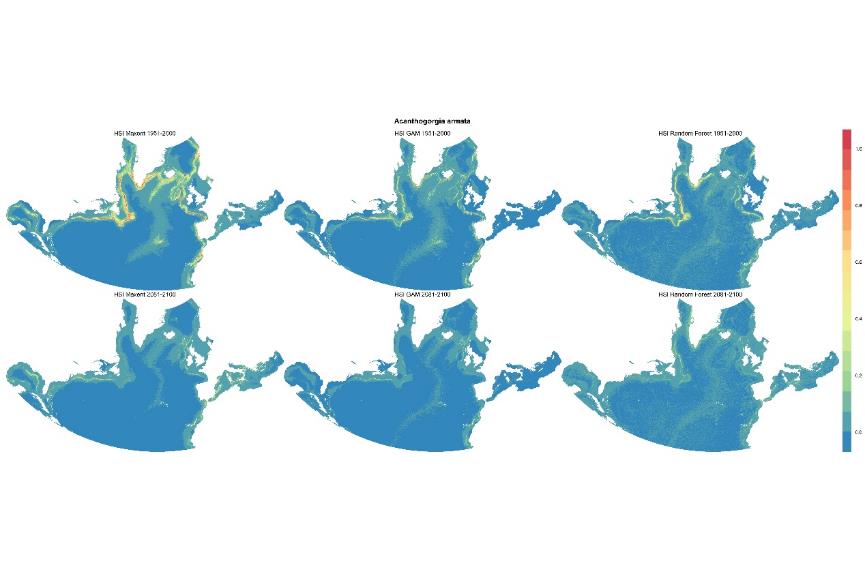

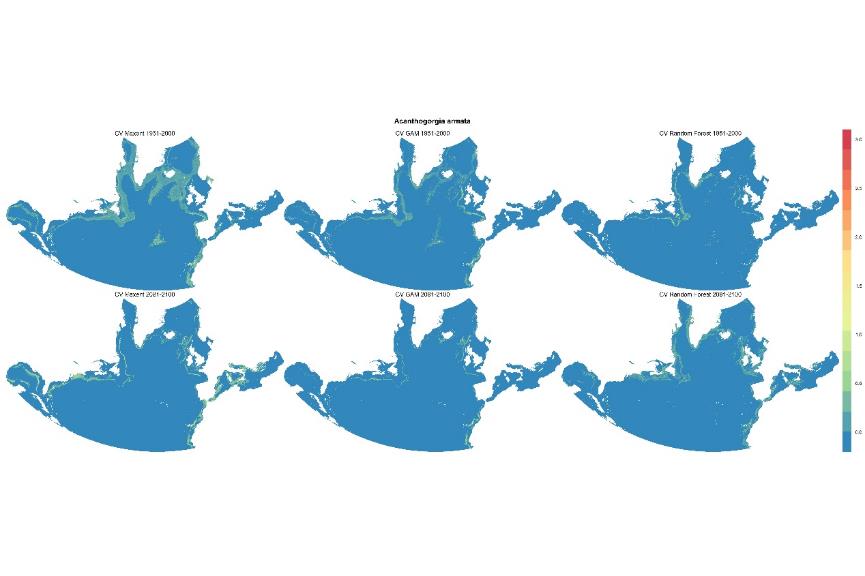
**

**Supplementary Figure S5d.** Predicted habitat suitability index (top) and Coefficient of Variance (CV; bottom) under present-day (1951-2000) and future (2081-2100; RCP8.5 or business-as-usual scenario) environmental conditions for the octocoral *Acanthogorgia armata* in the North Atlantic Ocean using three different modelling approaches: Generalized Additive Model (GAM), maximum entropy model (Maxent), and Random Forest (RF).

**
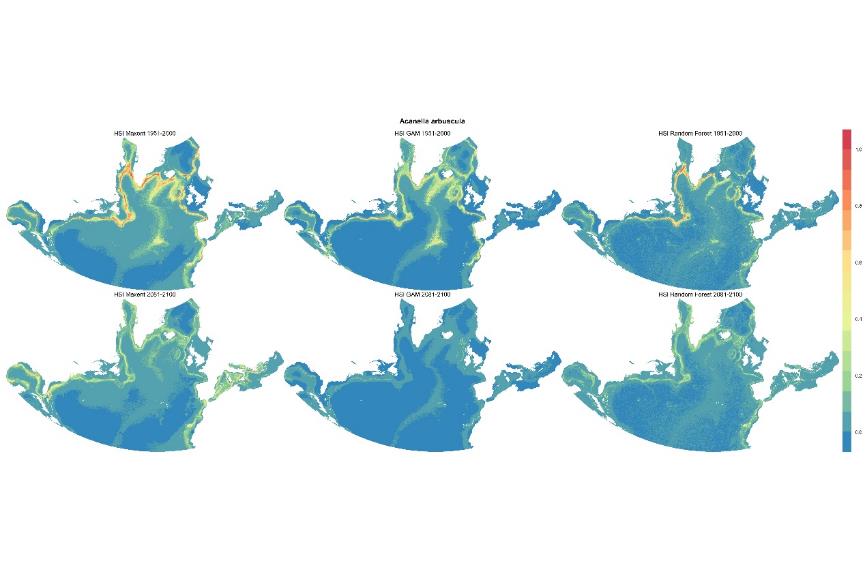

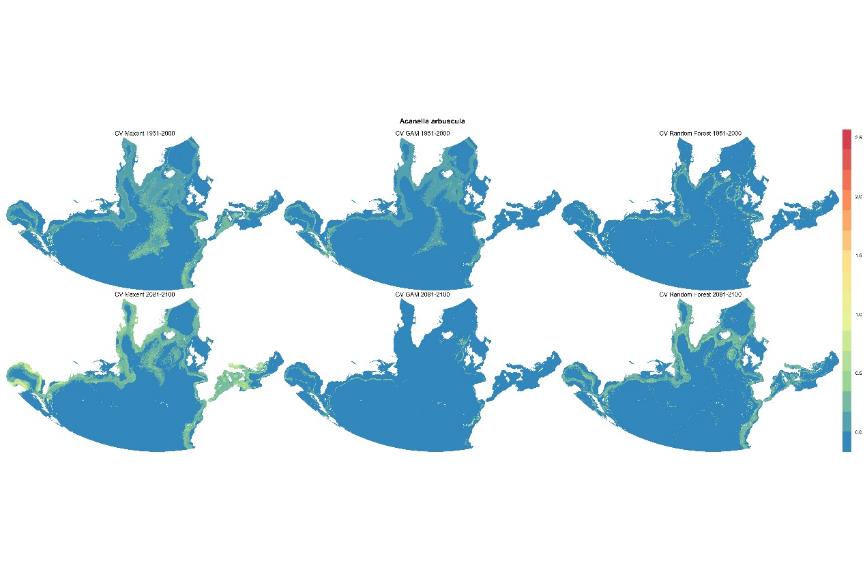
**

**Supplementary Figure S5e.** Predicted habitat suitability index (top) and Coefficient of Variance (CV; bottom) under present-day (1951-2000) and future (2081-2100; RCP8.5 or business-as-usual scenario) environmental conditions for the octocoral *Acanella arbuscula* in the North Atlantic Ocean using three different modelling approaches: Generalized Additive Model (GAM), maximum entropy model (Maxent), and Random Forest (RF).

**
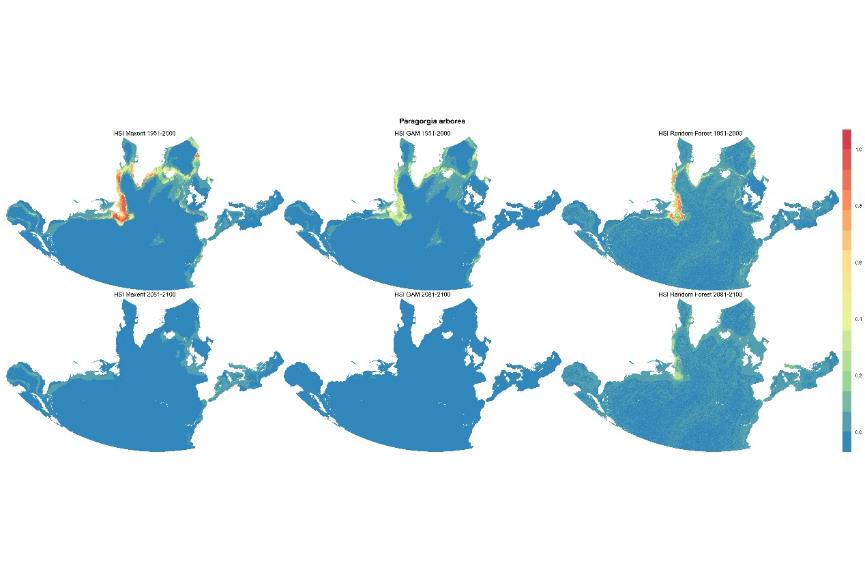

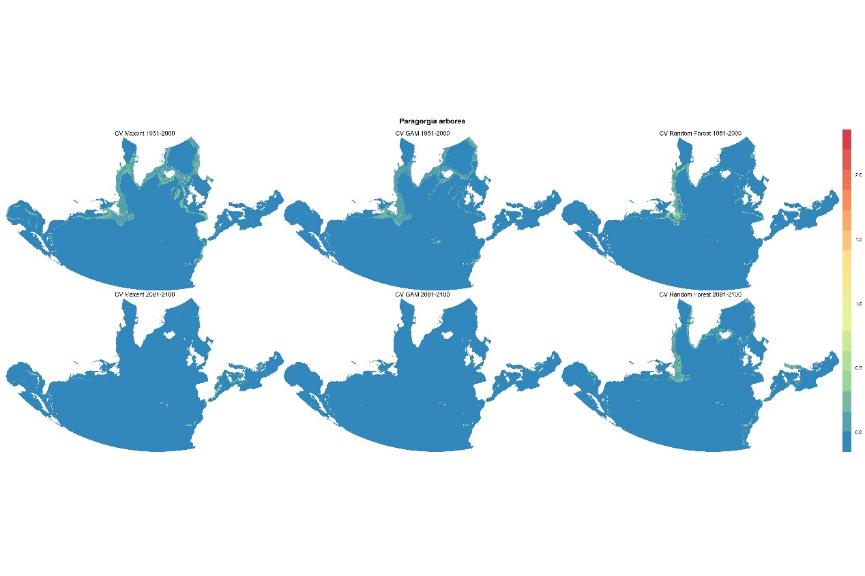
**

**Supplementary Figure S5f.** Predicted habitat suitability index (top) and Coefficient of Variance (CV; bottom) under present-day (1951-2000) and future (2081-2100; RCP8.5 or business-as-usual scenario) environmental conditions for the octocoral *Paragorgia arborea* in the North Atlantic Ocean using three different modelling approaches: Generalized Additive Model (GAM), maximum entropy model (Maxent), and Random Forest (RF).

**
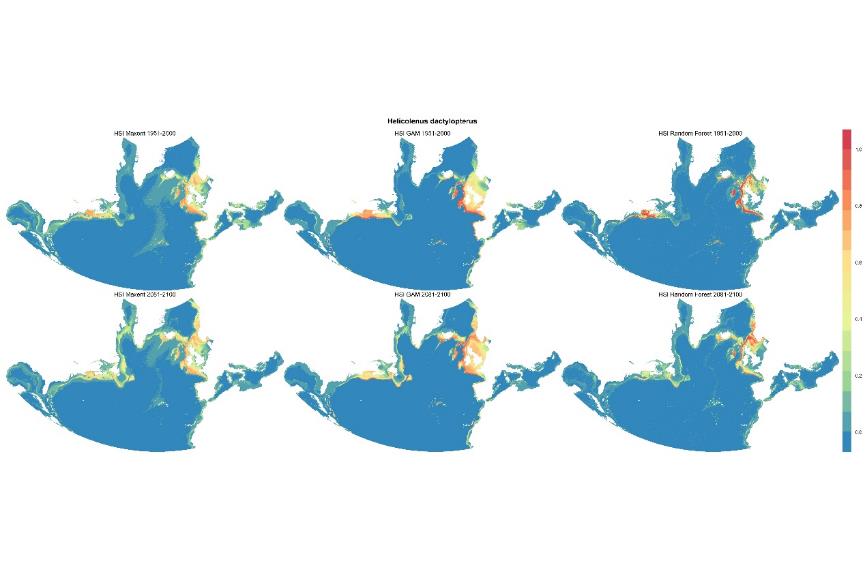

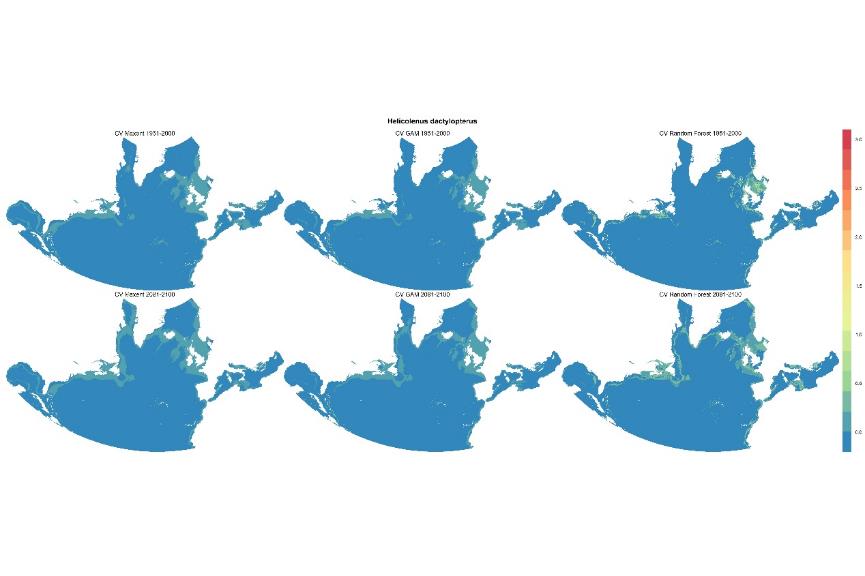
**

**Supplementary Figure S5g.** Predicted habitat suitability index (top) and Coefficient of Variance (CV; bottom) under present-day (1951-2000) and future (2081-2100; RCP8.5 or business-as-usual scenario) environmental conditions for the commercially important deep-sea fish *Helicolenus dactylopterus* in the North Atlantic Ocean using three different modelling approaches: Generalized Additive Model (GAM), maximum entropy model (Maxent), and Random Forest (RF).

**
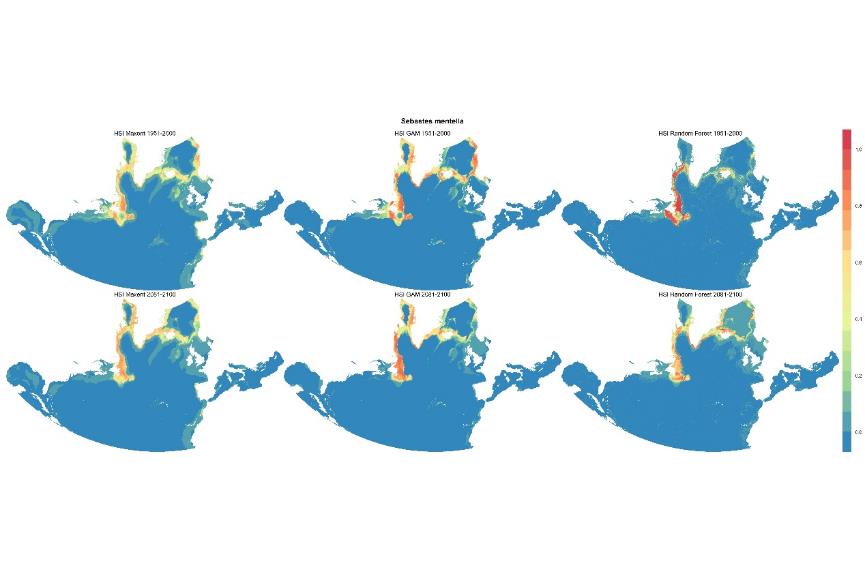

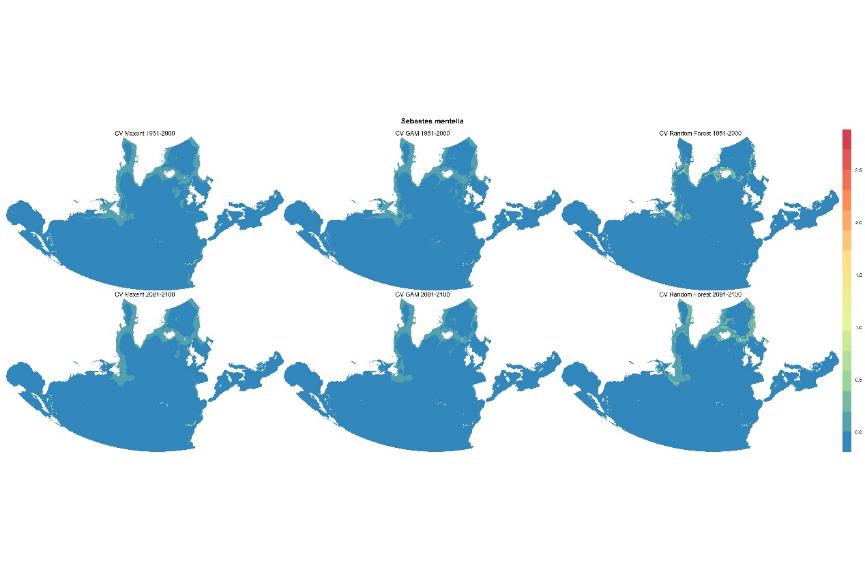
**

**Supplementary Figure S5h.** Predicted habitat suitability index (top) and Coefficient of Variance (CV; bottom) under present-day (1951-2000) and future (2081-2100; RCP8.5 or business-as-usual scenario) environmental conditions for the commercially important deep-sea fish *Sebastes mentella* in the North Atlantic Ocean using three different modelling approaches: Generalized Additive Model (GAM), maximum entropy model (Maxent), and Random Forest (RF).

**
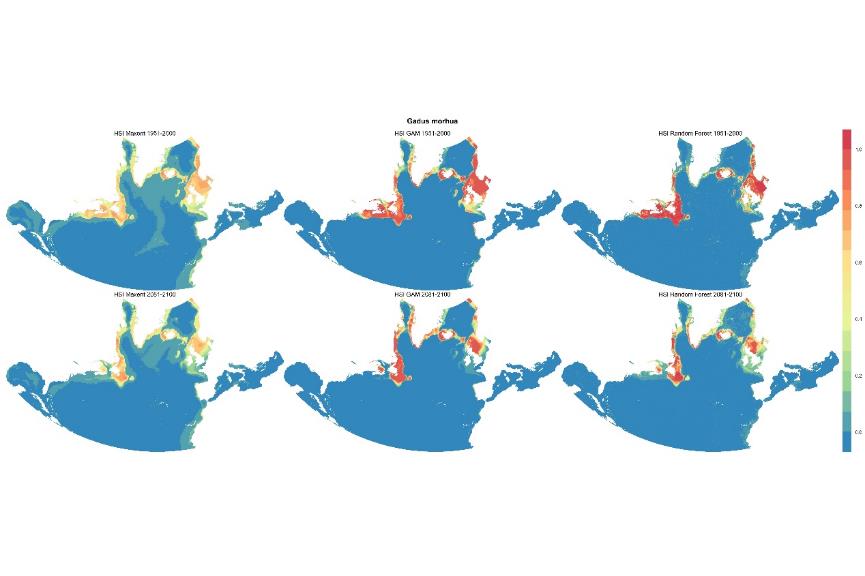

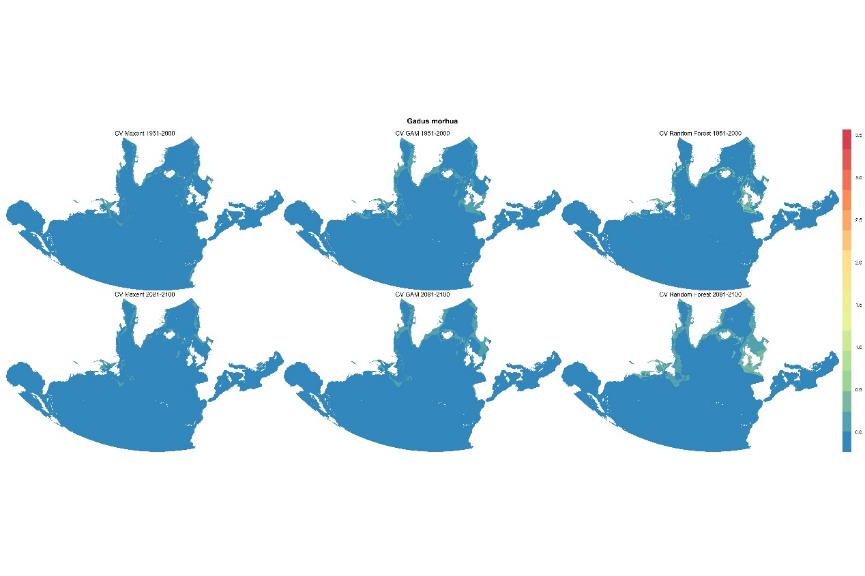
**

**Supplementary Figure S5i.** Predicted habitat suitability index (top) and Coefficient of Variance (CV; bottom) under present-day (1951-2000) and future (2081-2100; RCP8.5 or business-as-usual scenario) environmental conditions for the commercially important deep-sea fish *Gadus morhua* in the North Atlantic Ocean using three different modelling approaches: Generalized Additive Model (GAM), maximum entropy model (Maxent), and Random Forest (RF).

**
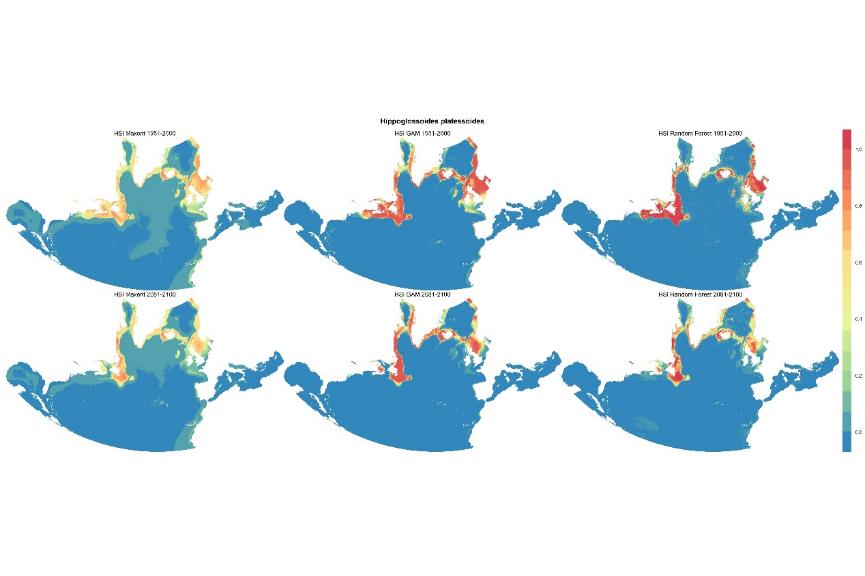

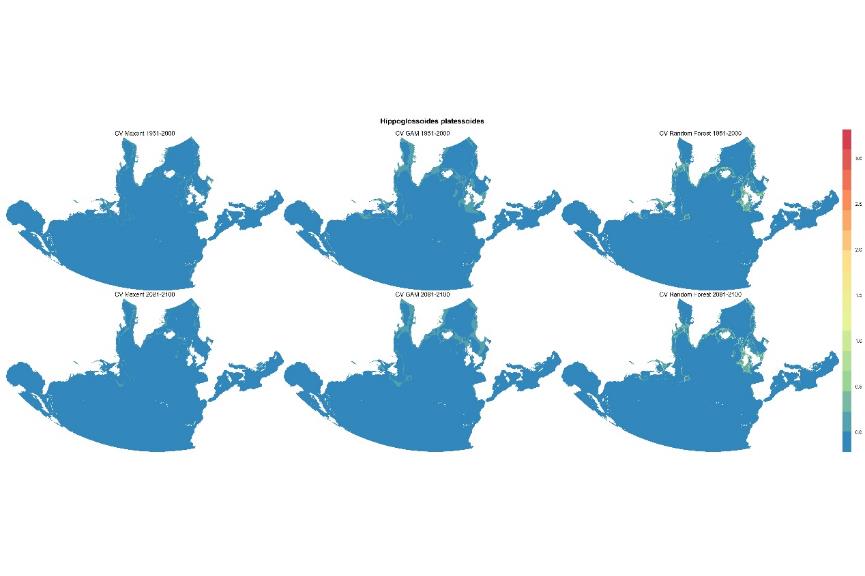
**

**Supplementary Figure S5j.** Predicted habitat suitability index (top) and Coefficient of Variance (CV; bottom) under present-day (1951-2000) and future (2081-2100; RCP8.5 or business-as-usual scenario) environmental conditions for the commercially important deep-sea fish *Hippoglossoides platessoides* in the North Atlantic Ocean using three different modelling approaches: Generalized Additive Model (GAM), maximum entropy model (Maxent), and Random Forest (RF).

**
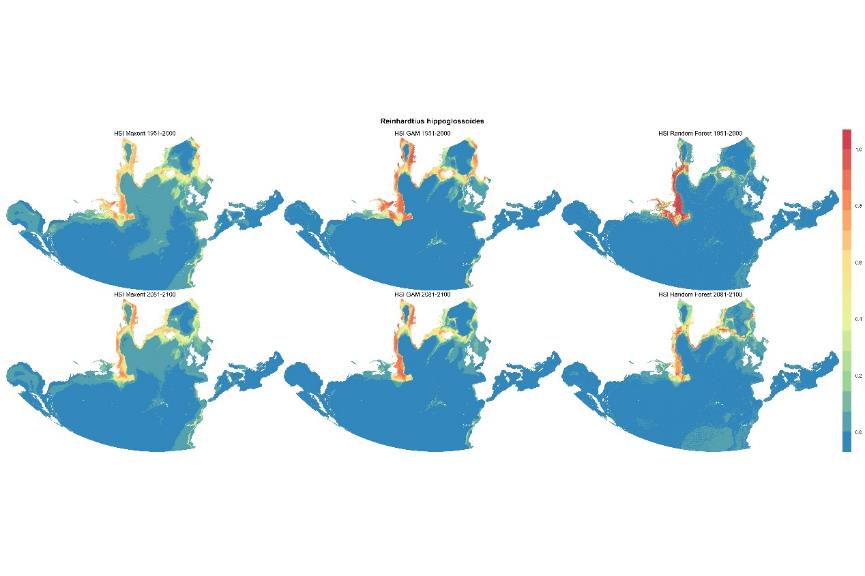

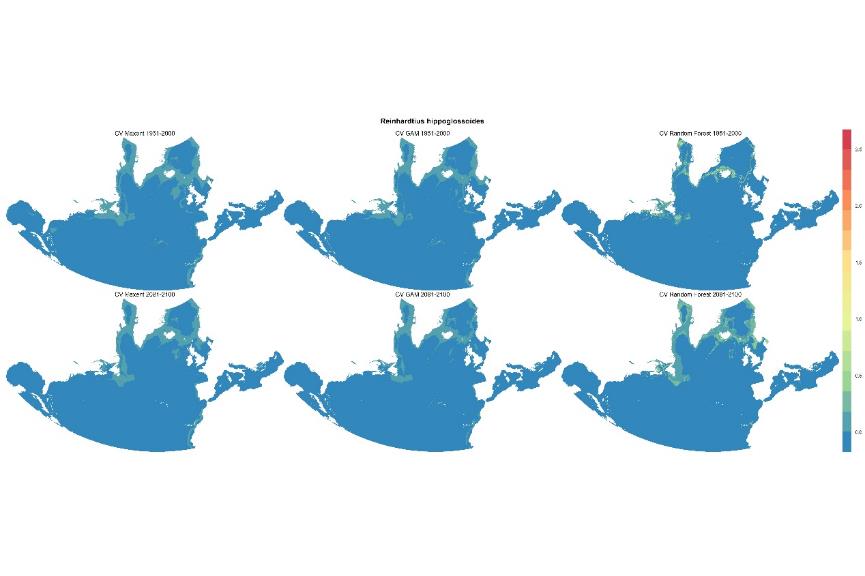
**

**Supplementary Figure S5k.** Predicted habitat suitability index (top) and Coefficient of Variance (CV; bottom) under present-day (1951-2000) and future (2081-2100; RCP8.5 or business-as-usual scenario) environmental conditions for the commercially important deep-sea fish *Reinhardtius hippoglossoides* in the North Atlantic Ocean using three different modelling approaches: Generalized Additive Model (GAM), maximum entropy model (Maxent), and Random Forest (RF).

**
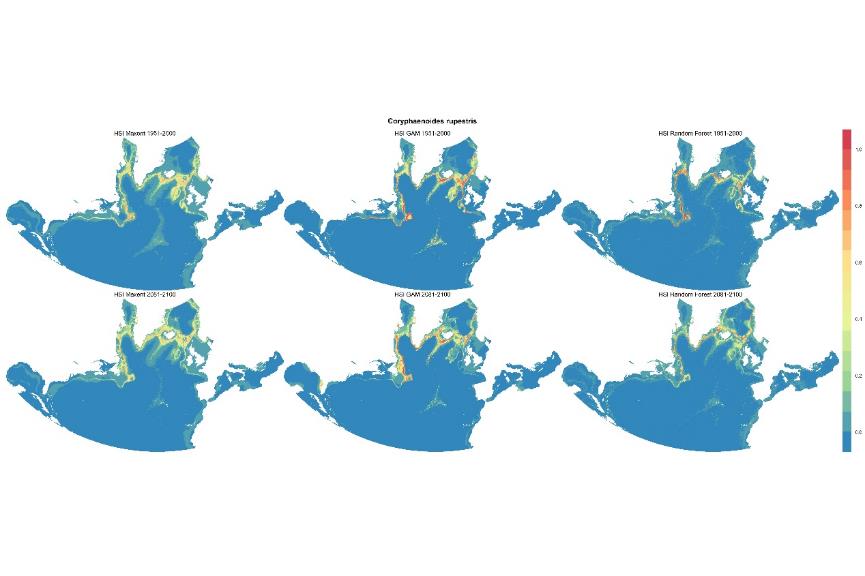

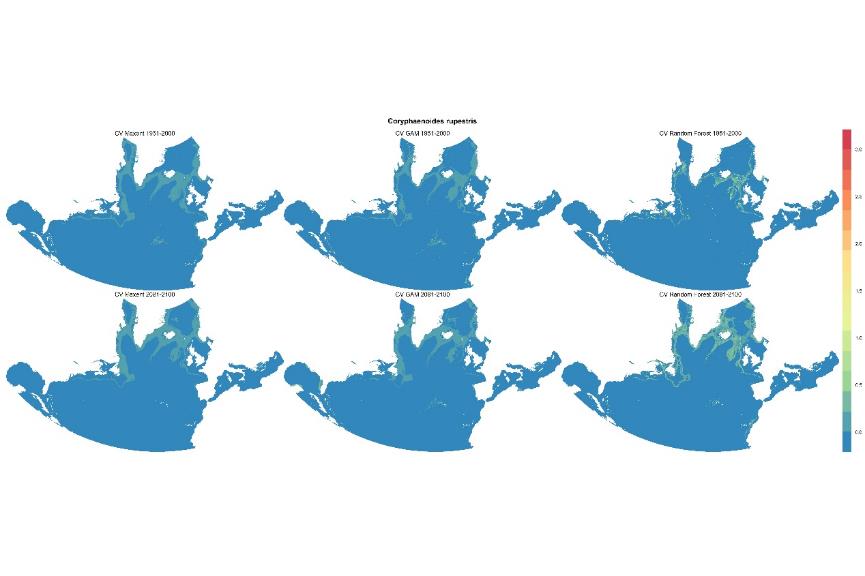
**

**Supplementary Figure S5l.** Predicted habitat suitability index (top) and Coefficient of Variance (CV; bottom) under present-day (1951-2000) and future (2081-2100; RCP8.5 or business-as-usual scenario) environmental conditions for the commercially important deep-sea fish *Coryphaenoides rupestris* in the North Atlantic Ocean using three different modelling approaches: Generalized Additive Model (GAM), maximum entropy model (Maxent), and Random Forest (RF).


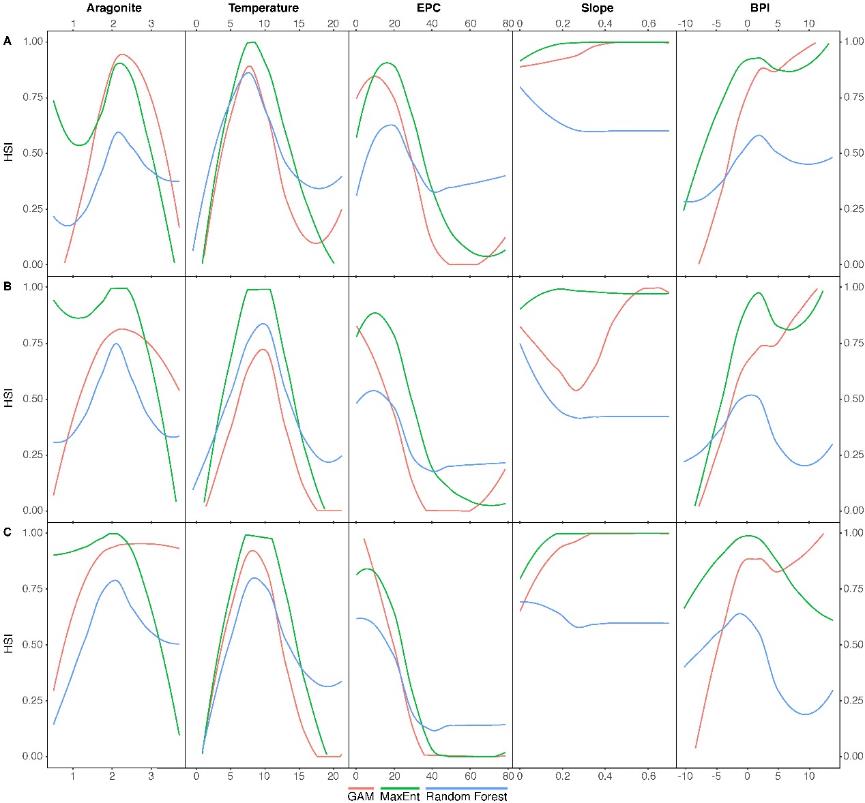


**Supplementary Figure S6a.** Response curves for predictor variables for scleractinian species as determined by the three different modelling approaches: Generalized Additive Model (GAM), maximum entropy model (Maxent), and Random Forest (RF). A) *Lophelia pertusa*; B) *Madrepora oculata*; C) *Desmophyllum dianthus*. Predictor variables included aragonite saturation state at seafloor, temperature at seafloor (°C), particulate organic carbon flux at the seafloor (EPC, mg C·m^-2^·d^-1^), slope (in radians) and standardized Bathymetric Position Index (BPI).


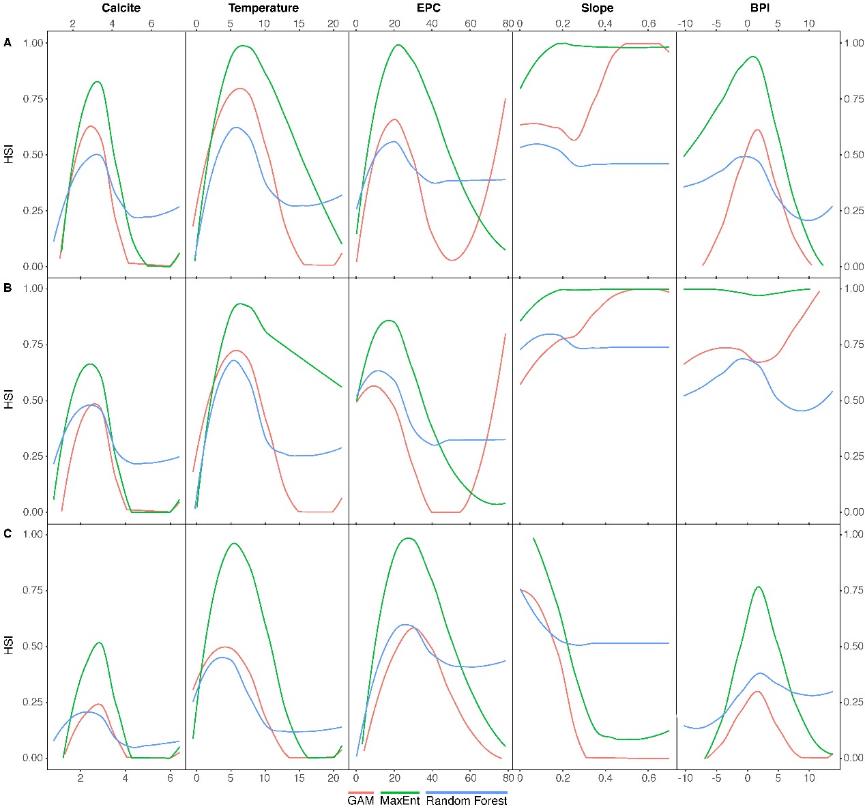


**Supplementary Figure S6b.** Response curves for predictor variables for octocoral species as determined by the three different modelling approaches: Generalized Additive Model (GAM), maximum entropy model (Maxent), and Random Forest (RF). A) *Acanthogorgia armata*; B) *Acanella arbuscula*; C) *Paragorgia arborea*. Predictor variables included calcite saturation state at seafloor, temperature at seafloor (°C), particulate organic carbon flux at the seafloor (EPC, mg C·m^-2^·d^-1^), slope (radians) and standardized Bathymetric Position Index (BPI).


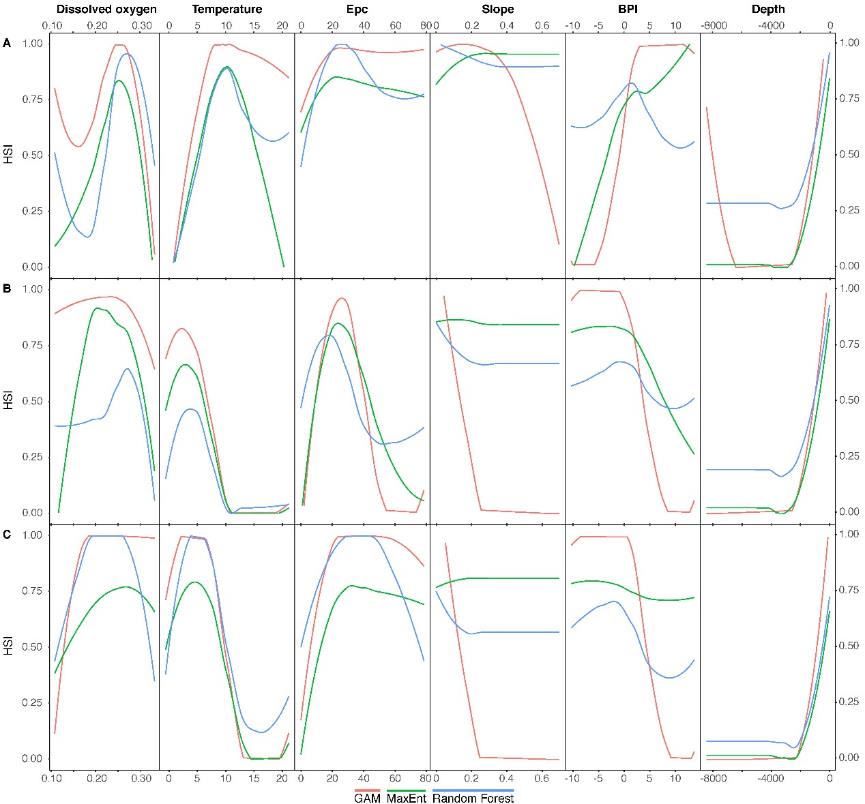


**Supplementary Figure S7a.** Response curves for predictor variables for commercially important deep-sea fish as determined by the three different modelling approaches: Generalized Additive Model (GAM), maximum entropy model (Maxent), and Random Forest (RF). A) *Helicolenus dactylopterus*; B) *Sebastes mentella*; C) *Gadus morhua*. Predictor variables included dissolved oxygen concentration at seafloor (mL·L^-1^), temperature at seafloor (°C), particulate organic carbon flux at the seafloor (EPC, mg C·m^-2^·d^-1^), slope (in radians), standardized Bathymetric Position Index (BPI), and depth (m).

**
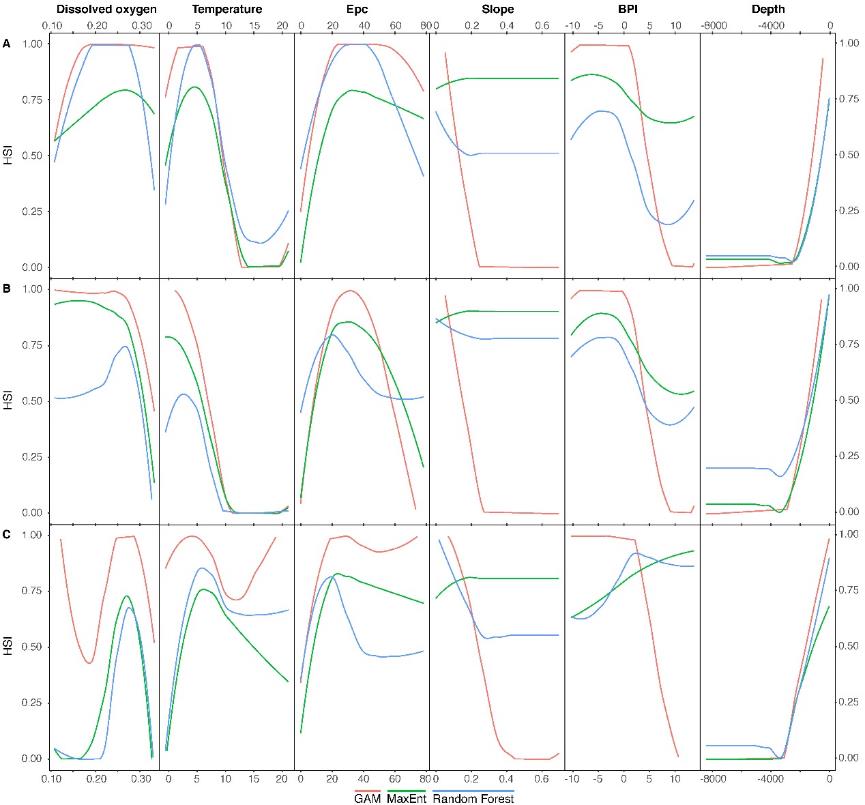
**

**Supplementary Figure S7b.** Response curves for predictor variables for commercially important deep-sea fish as determined by the three different modelling approaches: Generalized Additive Model (GAM), maximum entropy model (Maxent), and Random Forest (RF). A) *Hippoglossoides platessoides*; B) *Reinhardtius hippoglossoides*; C) *Coryphaenoides rupestris*. Predictor variables included dissolved oxygen concentration at seafloor (mL·L^-1^), temperature at seafloor (°C), particulate organic carbon flux at the seafloor (EPC, mg C·m^-2^·d^-1^), slope (in radians), standardized Bathymetric Position Index (BPI), and depth (m).


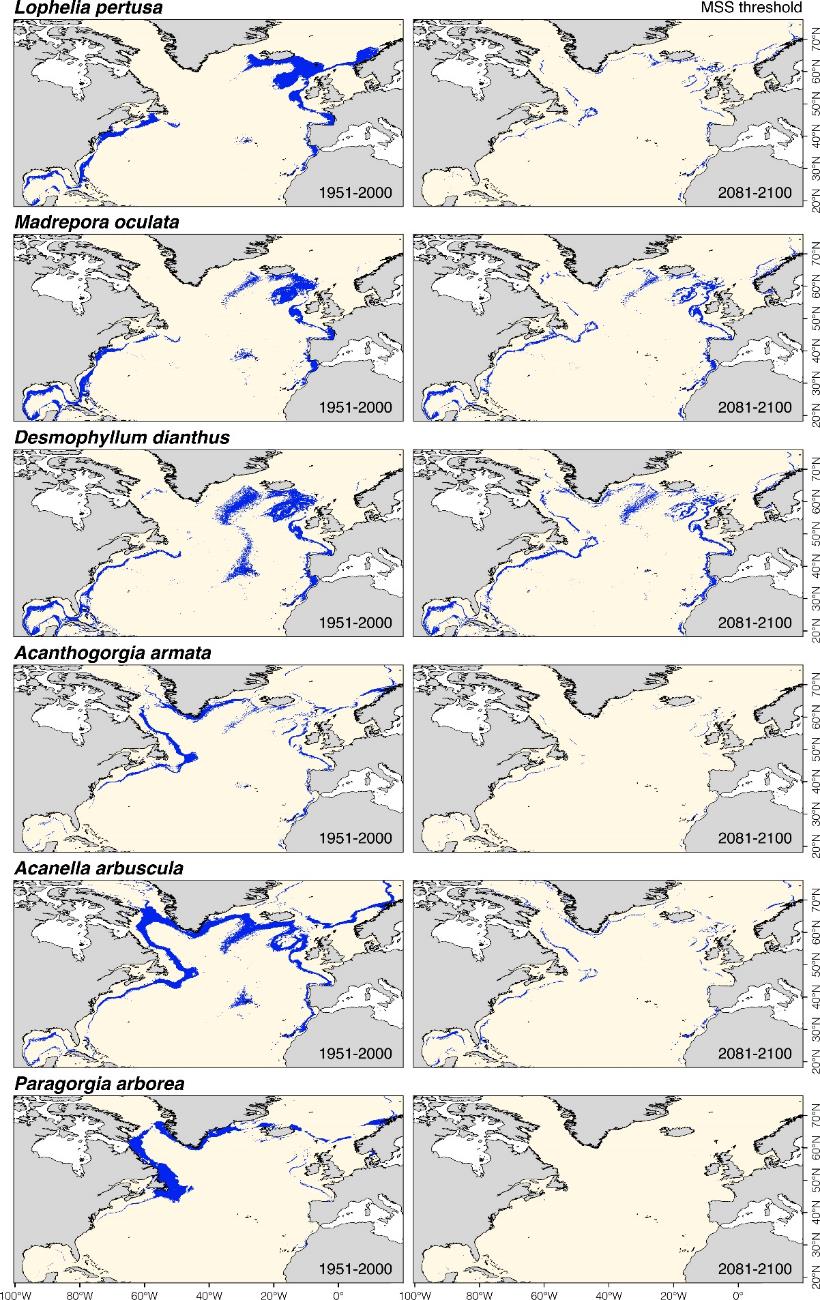


**Supplementary Figure S8a.** Climate induced changes in the suitable habitat of cold-water corals in the North Atlantic Ocean, as determined by binary maps built with an ensemble modelling approach and the maximum sensitivity and specificity (MSS) threshold.


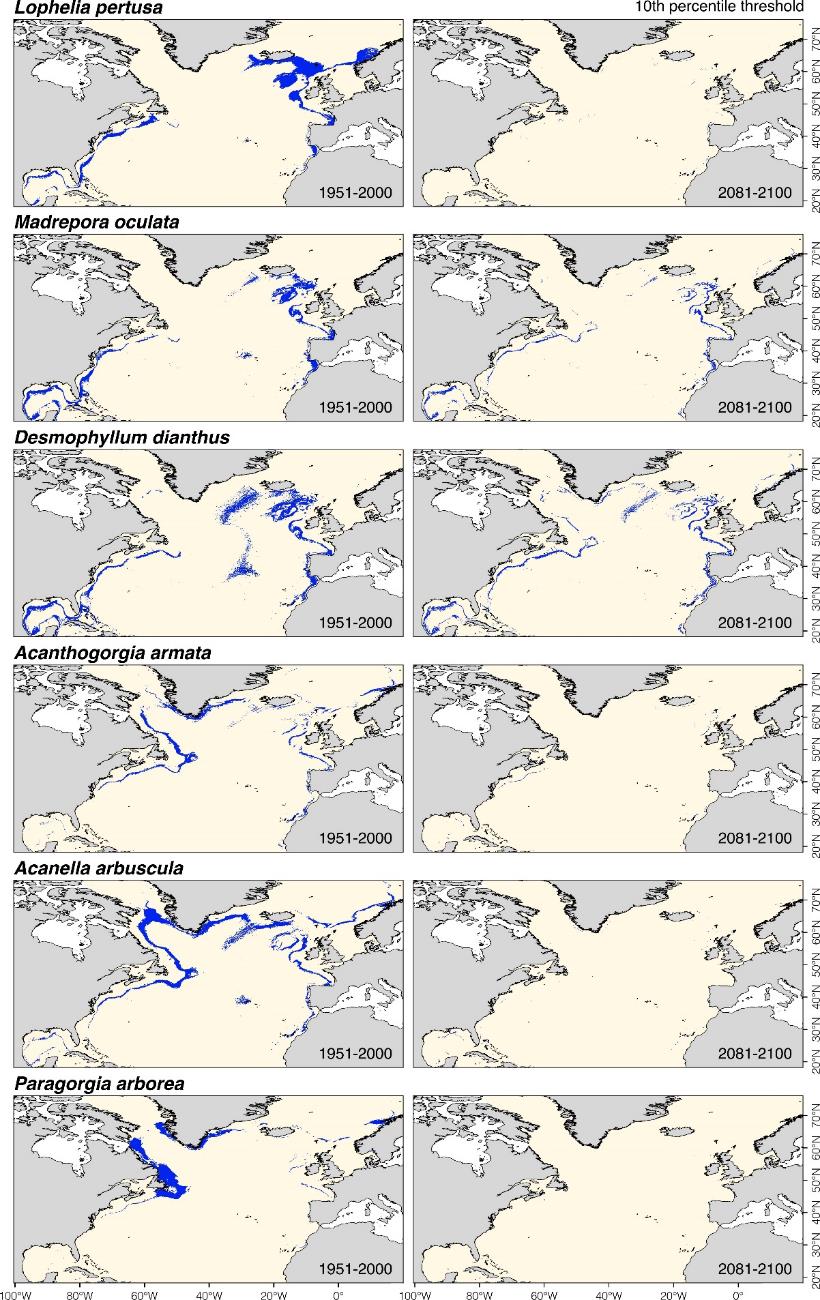


**Supplementary Figure S8b.** Climate induced changes in the suitable habitat of cold-water corals in the North Atlantic Ocean, as determined by binary maps built with an ensemble modelling approach and the 10^th^-percentile training presence logistic (10^th^ percentile) threshold.


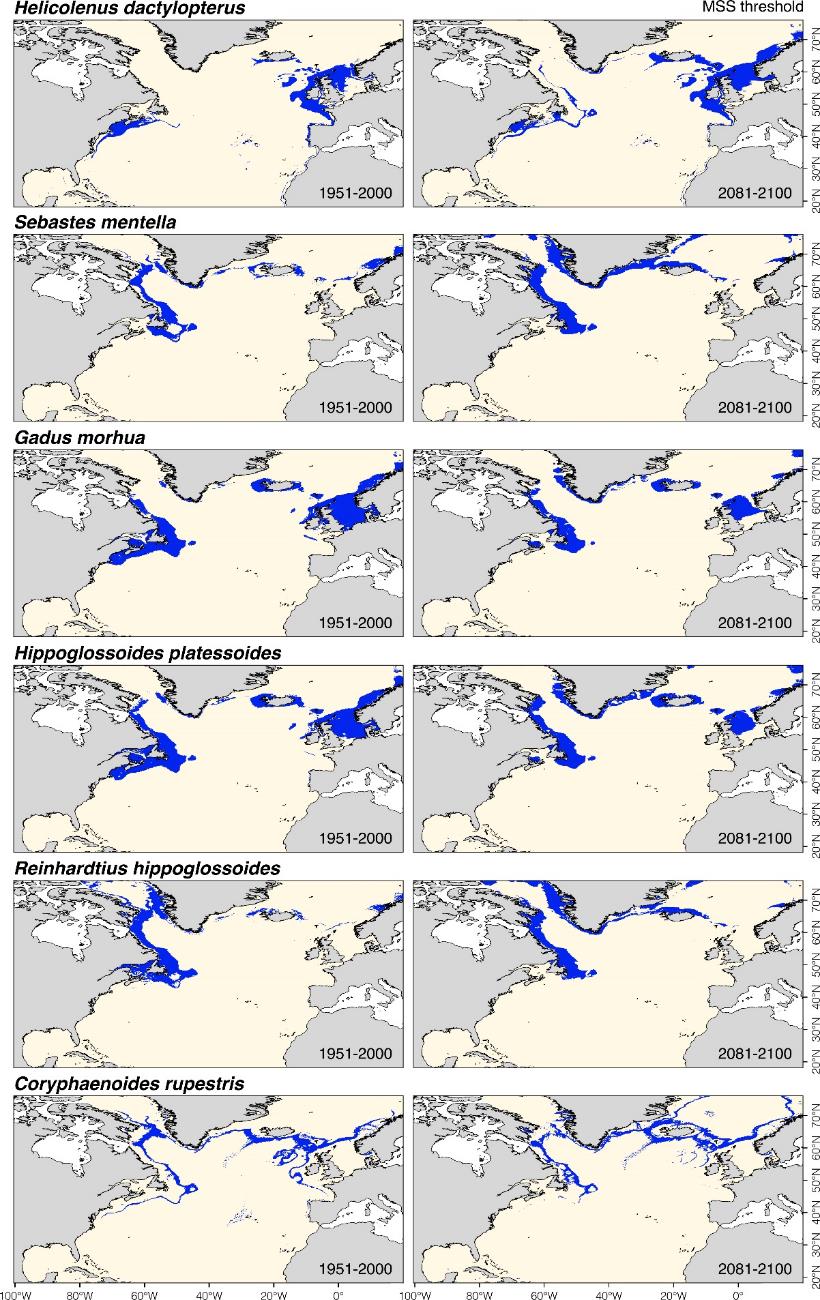


**Supplementary Figure S9a.** Climate induced changes in the suitable habitat of commercially important deep-sea fish in the North Atlantic Ocean, as determined by binary maps built with an ensemble modelling approach and the maximum sensitivity and specificity (MSS) threshold.

**
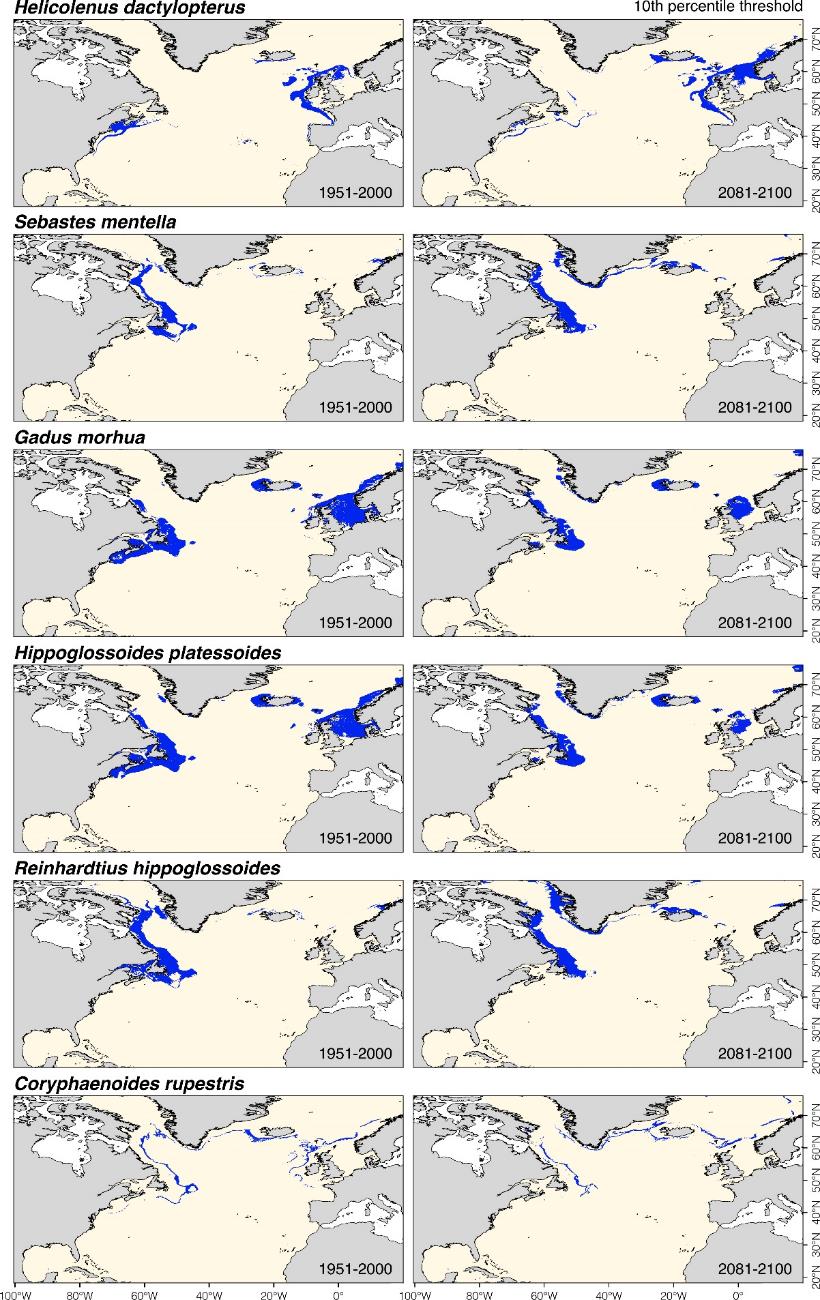
**

**Supplementary Figure S9b.** Climate induced changes in the suitable habitat of commercially important deep-sea fish in the North Atlantic Ocean, as determined by binary maps built with an ensemble modelling approach and the 10-percentile training presence logistic (10^th^ percentile) threshold.

1. recently synonymised to *Desmophyllum pertusum*, Addamo et al., 2016 [↑](#footnote-ref-1)
